# Counter-reporting sustainability from the bottom up: the case of the construction company WeBuild and dam-related conflicts

**DOI:** 10.1007/s10551-021-04946-6

**Published:** 2021-12-03

**Authors:** Antonio Bontempi, Daniela Del Bene, Louisa Jane Di Felice

**Affiliations:** 1 Geography Department, Autonomous University of Barcelona, Building B, Campus UAB, Bellaterra (Cerdanyola del Vallès), 08193 Barcelona, Spain; 2 Institute for Environmental Science and Technology, Autonomous University of Barcelona, Building ICTA-ICP (Z), Campus UAB, Bellaterra (Cerdanyola del Vallès), 08193 Barcelona, Spain

**Keywords:** Corporate social *ir*responsibility, Large dams, Hydropower schemes, Salini Impregilo, WeBuild, Counter-reporting, Post-normal science, Political ecology, Environmental justice, EJAtlas

## Abstract

Controversies around large-scale development projects offer many cases and insights which may be analyzed through the lenses of corporate social (*ir*)responsibility (CS*I*R) and business ethics studies. In this paper, we confront the CSR narratives and strategies of *WeBuild* (formerly known as *Salini Impregilo*), an Italian transnational construction company. Starting from the Global Atlas of Environmental Justice (EJAtlas), we collect evidence from NGOs, environmental justice organizations, journalists, scholars, and community leaders on socio-environmental injustices and controversies surrounding 38 large hydropower schemes built by the corporation throughout the last century. As a counter-reporting exercise, we code (un)sustainability discourses from a plurality of sources, looking at their discrepancy under the critical lenses of post-normal science and political ecology, with environmental justice as a normative framework. Our results show how the mismatch of narratives can be interpreted by considering the voluntary, self-reporting, non-binding nature of CSR accounting performed by a corporation wishing to grow in a global competitive market. Contributing to critical perspectives on political CS(I)R, we question the reliability of current CSR mechanisms and instruments, calling for the inclusion of complexity dimensions in and a re-politicization of CS(I)R accounting and ethics. We argue that the fields of post-normal science and political ecology can contribute to these goals.

**Supplementary Information:**

The online version contains supplementary material available at 10.1007/s10551-021-04946-6.

## Introduction

Everyone can say foolish things. You know what? There are five million people who believe that the Earth is flat. And they even vote – Pietro Salini, CEO of WeBuild.^1^

With these words, the CEO of the multinational construction company WeBuild, Pietro Salini, replied to an Italian journalist, addressing concerns raised by civil society organizations over the controversial impacts of the *Gibe III* hydropower scheme in Ethiopia (EJAtlas, 2020a). According to several scholars, media and environmental justice organizations, the dam has deprived hundreds of thousands of indigenous people living downstream of their livelihoods (ARWG, 2009; Carr, 2017; Franchi & Manes, 2016; Hodbod et al., 2019; Human Rights Watch, 2014a; OECD Watch, 2017; Survival International, 2021; The Oakland Institute, 2019). The Italian corporation, however, states in their 2016 Sustainability Report that “Gibe III has been designed and built with great care in terms of the effects on local communities, in order to mitigate its impacts and enhance its benefits” (Salini Impregilo, 2016b, p. 37).

Reality and truth are not the same for everyone, as philosopher of science Ravetz argues: “[…] any image of reality, being constructed within a particular system, simultaneously reveals, distorts and conceals” (Ravetz, 2006, p. 280). This is especially relevant in complex situations, when diverging beliefs and values are at play and forged by (strongly unequal) power relations. Under these circumstances, what claims, or grievances count as legitimate? What values reveal, distort, and conceal interests behind a particular system?

Controversies surrounding corporate social responsibility (CSR) disclosures are a case in point. CSR became popular both in the academy and in policymaking in the 1950s as an analytical tool to hold transnational corporations (TNCs) accountable for the ethics of their behavior (Carroll, 1999; Garriga & Melé, 2004; Taneja et al., 2011). Recently, engaged scholars have criticized CSR frameworks and indicators both theoretically and empirically for their weakness in including sustainability dimensions,^2^ or on the grounds that they may be used to hide irresponsible behavior (Cho & Patten, 2013; Kotchen & Moon, 2011; Lewis, 2016; Maher, 2019). The inconsistencies between CSR disclosures and from-the-ground facts or partial information about corporate behavior have been referred to as corporate hypocrisy (Antonetti et al., 2020; Delmas & Burbano, 2011). By unveiling corporate hypocrisy, viewed by many as an ethically unacceptable practice, the moral legitimacy behind the ‘social license to operate’ of TNCs can be questioned (Ehrnström-Fuentes, 2016; Ehrnström-Fuentes & Kröger, 2017; Gehman et al., 2017).

Building on this critical view, engaged academics introduced the concept of corporate social *ir*responsibility (CSIR), arguing that CSR narrowly points to practices, policies, controls, and procedures that are self-reported by corporations themselves (Maher, 2019; Riera & Iborra, 2017). From a political and post-colonial perspective, Banerjee (2003, 2008a, 2008b, 2010, 2011) has claimed that the very concept of CSR should be politicized, problematized, and deconstructed for the benefit of marginalized communities, by asking: “How can we create alternative structures of decision-making, conflict resolution and accountability?” (Banerjee, 2008a, p. 75).

In this paper, we address Banerjee’s call through a counter-reporting exercise,^3^ focusing on over six decades of civil works by WeBuild. We contrast the company’s CSR disclosures with alternative sources reporting from the ground. In doing so, we highlight high-level controversies tied to 38 large hydropower schemes linked with WeBuild. Our point of departure for the comparative analysis is the bulk of data on socio-environmental conflicts related to the Italian corporation retrieved from the Global Atlas of Environmental Justice—the EJAtlas (EJAtlas, 2021; Martinez-Alier, 2021; Temper et al., 2015, 2018). This is then further expanded through an exploration of the available sources of global evidence around the 38 contentious cases.

Empirically building on the scholarships of post-normal science, political ecology, and environmental justice, we aim to enrich critical thinking in the business ethics literature by problematizing the debate on CSR ethics, and by proposing the EJAtlas as a novel methodological tool for discussing CSR. While counter reports are recognized as powerful tools for the politicization of CSR (Gallhofer et al., 2006), they are rarely adopted from academia as they are considered costly (Macellari et al., 2021), or are relegated to campaigners and activists (Gallhofer et al., 2006). Adopting an activist-scholar spirit, we use our stance to amplify the voices and experiences that are left untold in the standard CSR framing, through the case study of a TNC operating in the global dam building market. Throughout our analysis, we ask: To what degree do CS(I)R reports account for environmental justice dimensions? Can the complexities of such dimensions be included in these forms of accounting?

Environmental justice struggles around large hydropower schemes provide many cases and insights which may be analyzed through the lenses of political CSR and business ethics. Supporters of such projects promise environmental and social benefits. However, evidence shows how the same projects disrupt local environments and displace populations. Despite extensive literature on the subject (Ansar et al., 2014; Bompan et al., 2017; Del Bene et al., 2018; Kirchherr & Charles, 2016; McCully, 2001), a growing number of dams is being planned and constructed as a strategy to cut down carbon emissions (Zarfl et al., 2014). As of 2018, approximately 70% of global renewable electricity supply was provided by hydropower (IEA, 2020). The sector has grown rapidly since the mid-2000s, due to the increased energy demand brought by the industrial sector, growth in urban consumption, and new funding and favorable policies for carbon–neutral energy sources (Steller, 2013). Private actors play an increasingly important role in this trend, as public–private partnerships are on the rise and new financing schemes with an important component of private capital are emerging (Braeckman et al., 2020). In this context, it becomes urgent to unpack the CSR discourses of dam building companies and to problematize their premises.

The article is structured as follows: after a theoretical and context background, we explain the methodology for data collection and analysis. We then present the results. Here, the dimensions of socio-environmental injustice that emerge from the review are codified into recurring categories and confronted with the CSR rhetoric of WeBuild’s sustainability reports. We provide first a general overview of the environmental injustices tied to 38 hydro-dams, and then focus more specifically on the unsustainability claims tied to emblematic cases. Finally, we discuss the results in the context of post-normal science, political ecology, environmental justice, and business ethics.

## Theoretical Framework

### Political CSR and Business Ethics

The politicization of CSR discourses and practices is a growing cue for discussion in business ethics and management, as well as organization studies (Scherer & Palazzo, 2011). Political CSR has been conceptualized with the aim of shifting from an instrumental view of CSR to a political one, that is morally informed and democratically driven (Palazzo & Scherer, 2006; Scherer & Palazzo, 2007, 2011). This perspective enables debates around governance, responsibility, democracy, and the legitimacy of CSR (Scherer & Palazzo, 2011). However, Scherer and Palazzo (2011) recognize the challenge for the business ethics literature in dealing with the complexities behind the post-national and post-modern constellation of actors and the related growing pluralism of values and norms: “the question remains of how the legitimacy of corporate activities can be normatively accessed when no universal criteria of ethical behavior are available” (ibid., p. 906). If the goal is to open the governance on CSR democratically to a highly diverse arena of actors, the dilemma can be solved by starting to give more weight to the voices that are left untold in standard CSR framing. Banerjee (2008a), Ehrnström-Fuentes (2016) and Hussain and Moriarty (2018) agree that there is a danger that marginalized or excluded stakeholders (who often coincide with the supposedly beneficiaries of corporate actions) may lack the organizational power needed to make their voices heard. Still, the question of how to involve non-corporate actors in the CSR deliberative process, and of who is included, needs further research (Banerjee et al., 2021; Hussain & Moriarty, 2018).

### Post-Normal Science

Post-normal science helps us tackle the irreducibility of perspectives and values in sustainability and CSR issues from the academia. Rooted in complexity science, post-normal science was conceived in the 1990s by Silvio Funtowicz and Jerome Ravetz in opposition to Thomas Kuhn’s “normal science” (Turnpenny et al., 2011). Post-normal science can be thought of as a new way of doing science, addressing issues where “facts are uncertain, values in dispute, stakes high and decisions urgent” (Ravetz, 1986, p. 422)—as in most, if not all, sustainability problems. Among the various directions that post-normal science research took (Turnpenny et al., 2011), the intention of providing a theoretical framework to improve the quality of the scientific process is particularly relevant for our scope. The idea is that science cannot hold “the truth” or be “true”, but it can have lower or higher quality depending on who was involved throughout the process, on the motivations behind it and on the frames used to describe a complex issue. For example, Ravetz critiqued Kuhn’s notion of scientific revolutions, arguing that science from the 1970s was increasingly corrupted as driven by military and industrial interests (Turnpenny et al., 2011). He called back then for the development of a critical science that would embrace a plurality of perspectives, singularities and unpredictabilities, generated outside of the military-industrial complex. Ravetz’s call is theoretically grounded in complexity science, where it is recognized that the same issue or situation may be viewed and described differently by different actors, and that these views are non-reducible to one another (Ahl et al., 1996; Mayumi & Giampietro, 2006; Rosen, 1991; Simon, 1991). This calls for approaches that embrace a plurality of voices (Mitchell, 2009). Embracing plurality does not mean that “anything goes” (Stirling, 2010), nor does it imply falling into epistemological anarchism (Feyerabend, 1993). Rather, plurality is intended here as a means to avoid the hegemonization of narratives, with the storylines constructed by powerful actors being the ones determining whether a process is sustainable or not. Thus, dominant narratives produced by those in power need to be confronted with alternative narratives built in decentralized ways outside of centers of power (Longhurst & Chilvers, 2019). In this context, traditional experts need to work with an extended peer community of those “affected by or with special knowledge of the issue” (Turnpenny et al., 2011, p. 292). The involvement of an extended peer community becomes a quality assurance measure, rather than an instrument to extend democracy (Yearley, 2000).

### Political Ecology & Environmental Justice

While it addresses the broad themes of complexity and quality, post-normal science does not unpack the power relations determining who gets to be involved in these extended peer communities and at what cost. Also, post-normal science scholars tend to be positioned within European academia and have seldom engaged with topics of environmental justice (for an exception of an analysis which combines post-normal science with environmental justice, see Porto (2012)). Political ecology provides a complementary framing to discuss the conflicts that emerge around environmental issues and the different power relations at play, with particular attention to marginalized groups (Bryant, 2015). As its ‘sister field’, environmental justice has traditionally focused on the unequal distribution of environmental costs and benefits across different geographies, issues of participation of affected communities, and the recognition of their claims, while acknowledging that vulnerable and discriminated communities and human groups are disproportionately subjected to higher risks of environmental threats than other (Schlosberg, 2007; Martínez-Alier, 2009). The environmental justice framework is very much relevant for business ethics, as corporate behavior is often a main driver for injustice, which in turn relates to the ethics and (ir)responsibility of the actions of TNCs. However, despite few exceptions (Benton, 2002; Hoffman, 1991; Maher, 2019; Nadeem, 2020; Oyewole, 2001; Ramirez, 2021), environmental justice is still under-used in business ethic studies.

### Counter-Reporting and the EJAtlas

While leveraging on post-normal science, political ecology, and environmental justice as theories, we draw on the concept of counter-reporting to develop our methodology. Among a wide range of deliberative practices in CSR studies that could be described as “accounting for the other, by the other” (Shearer, 2002; Tregidga, 2017, p. 511), Gallhofer et al. (2006, p. 681) define the concept of counter-accounting as “information and reporting systems employed by groups such as campaigners and activists with a view to promoting their causes or countering or challenging the prevailing official and hegemonic position”. Despite the emancipatory potential of these kinds of practices (Gallhofer et al., 2006; Gray et al, 2014), the approach is under-explored in academia, partially because of the high labor costs associated with it (Macellari et al., 2021). Building on the afore-mentioned literature, and opting for the concept of counter-reporting, we aim to contribute to the counter-accounting literature by relying on qualitative data from a diverse range of sources.

To this aim, we promote the use of the EJAtlas for the performance of counter-reporting in the field. The EJAtlas is currently the largest dataset of socio-environmental conflicts at the global level. As of March 2021, it catalogs evidence of more than 3300 conflicts. These are retrieved from different kinds of sources, ranging from NGO reports, media records and governmental sources to project-related documents (such as Environmental Impact Assessments (EIAs)). Recent academic publications on CSIR have drawn on the EJAtlas and contribute to establishing this tool for novel research in business ethics (Maher et al., 2021; Martinez-Alier, 2021; Saes et al. 2021).

## Context Background: WeBuild and its Business Branch of Dam Construction

WeBuild (webuildgroup.com) is today the major Italian industrial group specialized in construction and civil engineering works. It is the rebrand (from May 2020) of Salini Impregilo following its acquisition of Astaldi, another giant of the construction sector (WeBuild, 2020a). In turn, Salini Impregilo, founded in 2014, is the result of the merge of ten companies (Girola, Lodigiani, Torno, FIAT Impresit, Cogefar, Todini, Impregilo, S.A. Healy, Lane Industries, and Salini Costruttori) over more than one century of history. Table 1 resumes the main events that marked the history of the Italian industrial group.

**Table 1:** Main events that marked the history and development of today’s WeBuild SpA (Salini Impregilo, 2016a)

Year	Event
1906	Vincenzo Lodigiani and Umberto Girola decided to enter the construction market with their respective companies
1929	The main Italian automotive firm FIAT enters the construction sector, under the name of Impresit
1936	Pietro Salini (grandfather of the current CEO) starts his own construction company
1956	Impresit, Girola and Lodigiani start a joint venture (Impre.Gi.Lo) for the construction of the *Kariba* dam (Zambia-Zimbabwe)
1956	The re-established Salini Costruttori starts the construction of the *Legadadi* dam in Ethiopia
1959	Cogefar Costruzioni Generali is established
1960	Impregilo SpA is created from the merge of Impresit, Girola and Lodigiani
1984	The US company S.A. Healy is bought by the group
1989	Cogefar and Impresit merge into Cogefar-Impresit SpA
1994	Cogefar-Impresit, Girola, Lodigiani and Impresit-Girola-Lodigiani merge and become Impregilo SpA
2009	Salini Costruttori purchases Todini SpA
2014	Salini Impregilo SpA Group is born from the merge between Salini and Impregilo
2016	Salini Impregilo acquire 100% of the US company Lane Industries
2020	The group is renamed as WeBuild after the acquisition of Astaldi SpA

The company has been operating in more than 50 countries across the world. With a backlog of about 42 billion euros (WeBuild, 2020b), Salini Impregilo was listed by the American magazine Engineering News-Record as the worldwide unrivaled top international contractor in the water infrastructure sector for five years in a row (ENR, 2018). From the 1960s onwards, the group’s track record counts with more than 300 dams and hydroelectric plants for an installed power of 52,900 MW, including projects under construction (WeBuild, 2020c).

## Methodology

**Fig. 1:**
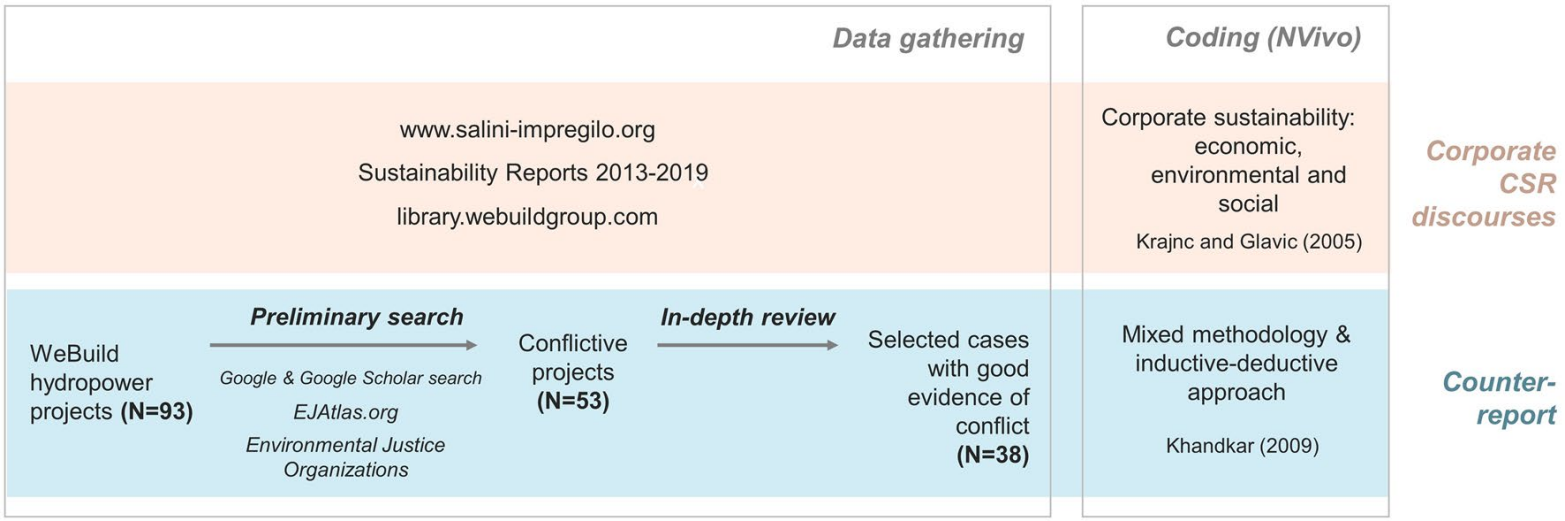
Methodological steps

This research was triggered by the extraordinary case of the Ethiopian *Gibe III* dam, which was shortly introduced at the beginning of the article. The extent of the impacts it caused and the magnitude of outrage it generated inspired and pushed us to investigate further. The Italian TNC was chosen then as the object of study after realizing that twenty high-intensity socio-environmental conflicts ascribable to dams that are acknowledged among WeBuild’s civil works were already registered in the EJAtlas database at the beginning of this study in 2019, proving the contentious presence of the company in various countries. The EJAtlas represents today the largest global database of socio-environmental conflicts. It was created in 2011 to give more visibility to conflicts, to collect data from the ground up, and to advance political ecology research toward large comparative and statistical analyses (Temper et al., 2018). It has involved hundreds of collaborators, both activists and academics. For further information on the EJAtlas rationale, see Temper et al. (2015). Most of these cases were already well known both by two of the authors of this article (as contributors of the EJAtlas) and in the international press, such as *El Quimbo* in Colombia (EJAtlas, 2019a), the *Lesotho Highland Water Project* in Lesotho (EJAtlas, 2020b), the *Grand Renaissance dam* in Ethiopia (EJAtlas, 2017a), the *Mosul dam* in Iraq (EJAtlas, 2017b), or *Chixoy* in Guatemala (EJAtlas, 2019b). Other cases are less known but just as dramatic, such as *Nathpa Jhakri* in Northern India (EJAtlas, 2015a) or the *Tokwe Mukorsi* dam in Zimbabwe (EJAtlas, 2017c). The methodology (schematized in in Fig. 1) was then conceived to explore the CSR frameworks and sustainability discourses of WeBuild and to build a counter-report based on alternative sources.

We first reviewed the company’s website (www.salini-impregilo.com, available until the rebrand into WeBuild), their sustainability reports published from 2013 to 2019 (for the years 2018 and 2019, these reports were included in the company’s consolidated financial statements) and publications (library.webuildgroup.com). The categories for the coding of Salini Impregilo-WeBuild reports were chosen based on the “triple bottom line” concept of sustainability, namely economic, environmental, and social, which is largely deployed in corporate reporting (Krajnc & Glavic, 2005).

To build our counter-reporting exercise, we conducted a review of existing sources of evidence for other potential controversial cases with the aim of broadening the information contained in the EJAtlas and of making the analyzed database as comprehensive as possible. We first identified all the dams featuring a hydropower component built across WeBuild’s history, retrieving the list from the company’s website and publications. For each of these projects, we conducted online research to characterize the following features: country, start and end date of the civil works, contractor, operating capacity (MW), and evidence (or not) of controversy, dispute, or conflict. We followed the conceptualization of socio-environmental conflicts proposed by Martínez-Alier and O’Connor (1996), who defined them as conflicts over the (unfair) distribution of environmental benefits and costs. This conceptualization was further developed in the political ecology and environmental justice literature, as those conflicts including mobilizations and protests around the lack of participation and recognition by local communities, and more broadly by environmental justice organizations, to refer to particular economic activities, infrastructure construction or waste disposal/pollution whereby environmental impacts are a key element of their grievances (Schlosberg, 2004). To collect the information about the dams, we associated the name of each dam to a set of keywords, as follows:

*[Name of the dam] AND (problem OR corruption OR liability OR conflict OR violence OR dispute OR impact OR police OR military OR protest OR strike OR controversy OR eviction OR resistance OR environmentalist OR opposition OR protest OR claim OR contested OR controversy OR resettlement OR victim OR survival OR threat OR demonstration OR affected OR homeless OR disruption OR abuse OR poverty OR development OR livelihood).*

The search was performed between January and February 2020 through the *Google* and *Google Scholar* search engines to capture both academic and non-academic types of sources; at first in English, and then repeated in Spanish, French or Italian, depending on the language spoken in the country where the dam was built. The same keywords were associated with the names of the companies that made up WeBuild (see Context Background and Table 1). This way, we retrieved cases that were associated to previous names of the company. This provided us with 53 cases that presented pieces of evidence of different forms of contention and conflicts, out of the 92 dams that were initially identified. We then excluded those cases with limited data (e.g., cases characterized by generic statements, statements not related to the specific case, those without proper references, or historic cases for which no online information could be found). Nevertheless, as even current conflicts remain underreported or completely invisible to the mainstream media, it was necessary to expand our information base. For those cases where little online information could be found, therefore, and to the extent of our capacity, we reached out to local organizations and front-line communities and activists to confirm data or provide additional sources. We relied on our own activist networks and on snowball methodology, as well as integrating the database with information from the social media profiles and webpages of environmental organizations, when possible. This process rests on the principles of the co-production of knowledge around socio-environmental conflicts, which is also at the core of the EJAtlas dataset (Temper & Del Bene, 2016). Evidence mainly comes from documentation produced from the ground up, i.e., from members of established organizations or collectives that have large social legitimacy amid environmental controversies. It includes press notes, declarations and statements released by the same organizations, reports, and other (non-academic) publications. This type of knowledge is often sidelined or dismissed in mainstream media or corporate reporting. When available, we complemented this information with academic and peer-reviewed publications. Our scientific methodology and political positionality aim therefore at abiding to both scientific and political rigor (Temper et al., 2019), as well as to the principles of quality in the scientific process suggested by post-normal science scholars.

The final dataset analyzed in this article includes 38 highly controversial dams. The sources of evidence related to these 38 cases were then coded. Codes were created starting from dimensions of environmental justice, such as the unequal distribution of goods and bads (e.g., environmental and socio-economic impacts), participation, and recognition (transparency and repression of dissent). We then complemented this with specific coding related to the construction of hydro-dams. Coding was both inductive and deductive (Khandkar, 2009), as we started from an environmental justice framework to capture claims and grievances but also included categories that strongly emerged from our sources and that did not fall in these categories. Codes were then refined throughout the analysis to best capture issues at stake.

NVivo was used to code both the reports of Salini Impregilo-WeBuild and the diverse sources found in relation to conflicts. The full codebook is included in the Supplementary Information.

## Results

### The Company’s CSR and Sustainability Discourses

The review of the Salini Impregilo-WeBuild website and publications reveals an image of an industrial group that is avant-gardist in its mission of CSR accomplishments. In 2013 Salini Impregilo subscribed to the United Nations (UN) Global Compact initiative and consequently adopted the UN Guiding Principles for Business and Human Rights (UNGC, 2021; Salini Impregilo, 2013, p. 3). Moreover, the company has included the UN Sustainable Development Goals within its sustainability policy since 2015 (Salini Impregilo, 2015a, 2016b, 2017a, 2018, 2019). Since 2013, the company has published annual sustainability reports (WeBuild, 2020d), where they self-account for their actions through an audit carried out by an independent third-party. Besides this, Salini Impregilo-WeBuild declares the adoption of an anti-corruption policy, a code of ethics (WeBuild, 2020e, 2020f), and an integrated management system in compliance with UNI EN ISO^4^ 9001 (quality management), UNI EN ISO 14001 (environmental management), UNI EN ISO 45001 (health and safety) international standards, all certified by an independent external body (Salini Impregilo, 2013, p. 12; WeBuild, 2020g). Moreover, WeBuild allegedly also operates in compliance with the OSCE Guidelines for multinational enterprises and with the principles of the ISO 26000 standard on ‘Social Responsibility’ (WeBuild, 2020g). In Table 2 we report a selection of CSR guidelines and standards that the company pledges to comply with or follow.

**Table 2:** References to a selection of CSR guidelines and standards that Salini Impregilo-WeBuild pledges to comply with

UN Business & Human Rights Guiding Principles
“The responsibility to respect human rights requires that business enterprises: (a) Avoid causing or contributing to adverse human rights impacts through their own activities, and address such impacts when they occur; (b) Seek to prevent or mitigate adverse human rights impacts that are directly linked to their operations, products or services by their business relationships, even if they have not contributed to those impacts.” (UNOHCHR, 2011, p 14)
UN Sustainable Development Goals (https://sdgs.un.org/goals)
“End poverty in all of its form everywhere” (#1)
“End hunger, achieve food security and improved nutrition and promote sustainable agriculture” (#2)
“Ensure healthy lives and promote well-being for all” (#3)
“Ensure availability and sustainable management of water and sanitation for all” (#6)
“Ensure access to affordable, reliable, sustainable energy for all” (#7)
“Protect labour rights and promote safe and secure working environments” (#8.8)
“Protect, restore and promote sustainable use of terrestrial ecosystems, sustainably manage forests, combat desertification, and halt and reverse land degradation and halt biodiversity loss” (#15)
“Promote peaceful and inclusive societies […], provide access to justice for all and build effective, accountable and inclusive institutions at all levels” (#15)
UNI EN ISO 9001 (quality management system standards)
“When planning for the quality management system, the organization shall […] determine the risks and opportunities that need to be addressed to […] prevent, or reduce, undesired effects” (ISO 9001:2015(E), 6.1.1, p. 4)
UNI EN ISO 14001 (environmental management system standards)
“requires an organization to identify the environmental aspects arising from the organization's past, existing or planned activities, products and services, in order to determine the environmental impacts of significance” (ISO 14001:2004(E), A.1, p 10)
ISO 26000 (social responsibility)
[an organization should] “be accountable for its impacts on society, the economy and the environment” (ISO 26000:2010(E), 4.2, p. 10) “[…] be transparent in its decisions and activities that impact on society and the environment (ibid., 4.3, p. 10)” […] respect, consider and respond to the interests of its stakeholders. (ibid., 4.5, p. 12) “[…] respect human rights”
Global reporting initiative standards
“The reporting organization shall report […] a description of its significant economic, environmental and social impacts, and associated challenges and opportunities. This includes the effects on stakeholders and their rights as defined by national laws and relevant internationally-recognized standards” (Disclosure 102–15 of GRI 102, 2016, p. 15)

The firm boasts several awards (WeBuild, 2020h). It appears in the Top 11 Open Corporation ranking, a project led by the Italian union Filcams-Cgil and co-financed by the European Commission (Open Corporation, 2021; Salini Impregilo, 2017b). In addition, they rank third in the Social Reporting Transparency Index for companies with the “best sustainability reports” (Salini Impregilo, 2017b), and eighth in the Total Transparency Index (ibid.), with their website winning the NC Digital Awards for “best storytelling” (Salini Impregilo, 2015a, p. 13). Salini Impregilo adopted the Global Reporting Initiative guidelines in preparing its reports (Salini Impregilo, 2013, p. 3), and is included in the Carbon Disclosure Project (CDP)’s Climate A List (Salini Impregilo, 2015a, p. 13; WeBuild, 2020h). With regards to Environmental, Social and Governance (ESG) ratings, WeBuild claims to be a “benchmark of excellence” (WeBuild, 2020i), scoring high in various assessments by main rating agencies (EcoVadis, Morgan Stanley Capital International, ISS, VigeoEiris).

In the vision of the industrial group, big infrastructure is a desirable key necessity for the well-being of future generations (Salini Impregilo, 2016a, p. 7). Dams and hydropower plants are framed as tools to reduce carbon emissions and regulate waterflows while simultaneously raising countries' economic potential, especially needed in those 'poor' (Southern) countries that lack large infrastructure development (Salini Impregilo, 2015b; Salini Impregilo, 2016a, p. 158). These corporate sustainability discourses have been coded and shown in Table 3, and mapped onto three sustainability dimensions: economic, environmental and social.

**Table 3:** Codification of Salini Impregilo’s sustainability narratives

Codes	Illustrative quotes
Economic sustainability	
Energy security	“Access to energy represents one of the major pillars for the development of society. Yet the current fossil fuel-based energy system leaves about 1.4 billion people around the world without access to electricity” (Salini Impregilo, 2013, p. 9)
GDP growth (at country and local levels)	“Salini Impregilo aims to develop infrastructure projects that act as catalysts for growth in the countries where we operate” (Salini Impregilo, 2013, p. 21) “The Group is committed to expanding opportunities for suppliers of goods and services in every host country by prioritising local sourcing, wherever possible” (Salini Impregilo, 2015a, p. 21)
Green economy	“Under the ‘green economy’ paradigm, economic growth and socio-environmental sustainability are viewed not as incompatible, but as mutually reinforcing” (Salini Impregilo, 2013, p. 9)
Infrastructure need	“Robust infrastructure is the lifeblood of strong economies and societies, playing a major role in industrial, agricultural, rural and urban development” (Salini Impregilo, 2015a, p. 15)
Job creation	“In addition to the direct workforce, the construction industry makes regular use of subcontractors for certain activities, as well as other providers of services (including technicians, consultants, catering staff, etc.), which contribute significantly to the number of jobs created at the local level. In 2014 around 16,700 people were employed by our subcontractors, and another 2900 by related service providers, 74% of whom were hired locally" (Salini Impregilo, 2014, p. 24) “We engaged nearly 15,000 people to participate in the projects at the end of 2015, 97% of whom come from rural communities nearby” (Salini Impregilo, 2015a, p. 32)
Poverty alleviation	“Infrastructures improvement is inextricably linked with poverty alleviation, particularly in low-income countries, where better infrastructure can provide a safety net against natural disasters and economic shocks” (Salini Impregilo, 2013, p. 9)
Revenues creation (for shareholders; from labor income)	“Our shareholders and investors benefited from the growth of our revenue (+ 13.6%) and backlog (+ 9.9% in the construction and plant sector) in terms of return on their investments and share value (+ 108.57%);” (Salini Impregilo, 2013, p. 3) “The Company has operating procedures and practices designed to ensure that its remuneration policies comply with the regulations applicable in all the countries where the Group operates and especially the minimum wage requirements, where these exist” (Salini Impregilo, 2017a, p. 154)
Environmental Sustainability	
Circular economy	“Improving performance throughout the infrastructure’s life cycle” (Salini Impregilo, 2016b, p. 52)
Clean, renewable energy	“Through our projects we play an important role in combatting climate change” (Salini Impregilo, 2013, p 46)
Impact assessment	“All potential environmental impacts deriving from our construction activities are assessed according to a standardised methodology, based on specific criteria (probability of occurrence, consequences for the environment, duration of the event, difficulty of restoration). Following the impact assessment, each project prepares an Environmental Management Plan, which describes the management and monitoring activities (Environmental Control Plans) for all environmental components involved” (Salini Impregilo, 2014, p. 41)
Impact mitigation	“We are committed to reclaiming all areas affected by our plants, facilities, quarries and landfills during or after the completion of a project. The aim is to leave these areas in a condition that facilitates natural re-vegetation, prevents soil erosion, improves slope stability, returning affected areas to their original state” (Salini Impregilo, 2013, p. 50)
Social sustainability	
Anti-corruption policy	“Salini Impregilo has a zero tolerance policy for all types of corruption and is committed to complying with the anti-corruption laws ruling in all the countries where it operates”. (Salini Impregilo, 2017a, p. 179)
Community support – capacity building for locals	“Local road improvements, electricity supply, sport facilities construction” (Salini Impregilo, 2013, p. 16) “We have realized dozens of projects in recent years, including schools, health centres, public offices, water networks, roads and bridges” (Salini Impregilo, 2013, p. 27) “A total of 207 social programmes were carried out over the period 2012–2014 (48 in 2014)” (Salini Impregilo, 2014, p. 27)
Ethics	“Code of Ethics, which defines for each corporate value the principles that guide our behaviours. These include honesty, fairness, integrity, impartiality, confidentiality, physical integrity protection, respect for human dignity, environmental protection, and the respect of local communities” (Salini Impregilo, 2015a, p. 38)
Human rights (of local populations and workers)	“The Group supports the rights enshrined in the International Bill of Human Rights and the International Labour Organization conventions” (Salini Impregilo, 2016b, p. 78)
Stakeholders' engagement	“For Salini Impregilo, building shared growth also means interacting with and supporting the communities that live near our sites. We have a longstanding commitment to understanding the cultures, needs, and expectations of those communities. For example, we seek to integrate our sites with the surrounding areas by deepening our knowledge of the country and local area, and regularly engaging with communities” (Salini Impregilo, 2015a, p. 22)
Work safety and health; training to employees	“We pay particular attention to the provision of good living conditions for personnel employed in remote areas and challenging socio-environmental contexts” (Salini Impregilo, 2013, p. 38)

We found that the reporting is mostly not case-specific, and data (on economic, environmental and social dimensions) are aggregated—that is, numbers that are reported, such as GDP growth or land restoration figures, tend to be macro-scale figures which are difficult to map onto specific projects or processes. In the few cases where the context of a particular project is mentioned, the reports celebrate the ways in which the company benefits local populations, or the environment. For example, in Uganda, when delivering the *Bujagali* plant, Salini Impregilo partnered with an NGO dealing with oncological treatments (Salini Impregilo, 2013, p. 29); in the frame of the *Tocoma* dam in Venezuela, a vocational training program was developed to teach sustainable farming to local communities (*ibid*., p. 29); in Malaysia, the company claims that the local communities impacted by the *Ulu Jelai* dam would benefit from road improvements, electricity supply and the construction of sports facilities (*ibid*., p. 16). The story that emerges from a book that celebrates their 110 years of history is one they “are proud to tell” (Salini Impregilo, 2016a, p. 7).

The reports never mention potential or actual negative impacts of the works they have been involved in. Yet, as the results of our research in the next section show, the version portrayed by the corporation substantially differs from alternative sources.

### Unsustainability Claims

In this section, we provide an overview of the results from the counter-reporting exercise that we described in the methodology. We start by providing an overview of the types of injustices linked to the 38 selected projects; then, we focus on discussing in more detail select emblematic cases. For this second part, we trace the unsustainability claims tied to the cases with a geographical and chronological approach.

Table 4 lists the 38 dams that were included in the analysis. For each dam, where retrievable, we report information on the name, country, year (start and end of civil works), capacity (MW) and references. The codebook that systematizes the information on the various sources is provided in Table 5, where for each (sub)code we report references to emblematic quotes and dams (the full source documents are provided in the Supplementary Information). This set of codes does not overlap with the one obtained from the analysis of company’s disclosures (Table 3): this is because the narratives deployed by claimants and by the corporation are inherently different and sometimes diverging.

**Table 4:** List of hydropower schemes included in the analysis

Name	Country	Civil works		Company	Capacity (MW)	Main references
		Start	End			
Kariba	Zambia-Zimbabwe	1956	1959	Impregilo	600	Darbourn (2015); EJAtlas (2017d); International Rivers (2009); Lang et al. (2000); Scudder (2005)
Dez	Iran	1959	1963	Impregilo	520	Lang et al. (2000);
Akosombo	Ghana	1961	1965	Impregilo	912	EJAtlas (2016a); Hilton (1966); Lang et al. (2000); Miescher (2014)
Kainji	Nigeria	1964	1999	Impregilo	760	EJAtlas (2014a); Lang et al. (2000)
Tarbela	Pakistan	1968	1974	Impregilo	3′478	Bennet and McDowell (2012); EJAtlas (2019c); Lang et al. (2000);
Kossou	Ivory Coast	1969	1972	Impregilo	174	Pittalunga et al. (2002); Prowizur (1976); Raphaël et al. (2019)
Chivor	Colombia	1970	1982	***	1′000	Semana (2019)
James Bay	Canada	1974	1981	Salini Costruttori	5′616	Curran (2012); EJAtlas (2020c); Wall (2017)
Itezhi-Tezhi	Zambia	1974	1978	***	120	Godet and Pfister (2007); Lang et al. (2000)
Chixoy (Pueblo Viejo)	Guatemala	1976	1983	Cogefar	281	Colajacomo (1999); EJAtlas (2019b); GHRC (2011); Johnston (2010); Lang et al. (2000); Manes (2012)
El Cajón	Honduras	1980	1985	Impregilo	300	Lang et al. (2000); McCully (2001)
Betania	Colombia	1981	1988	***	510	Galindo Vanegas (2018)
Mosul (Saddam)	Iraq	1981	1985	Impregilo	750	Al-Ansari et al. (2020); Bender (2014); Borger (2016); EJAtlas (2017b); Filkins (2016)
Bumbuna	Sierra Leone	1982	2009	Salini	143	D'Angelo (2014); EJAtlas (2020d); Mazzei and Scuppa (2006)
Daule Peripa	Ecuador	1982	1987	Impregilo	213	EJAtlas (2019d); Gerebizza (2009)
Yacyreta	Argentina—Paraguay	1983	1988	Impregilo	3′100	EJAtlas (2019e); Lang et al. (2000)
Piedra del Aguila	Argentina	1985	1993	Impregilo	1′400	Balazote and Radovich (2003)
Ertan	China	1987	1998	Impregilo	3′300	Lang et al. (2000)
Lesotho Highlands Water Project	Lesotho	1989	**	Impregilo	110	EJAtlas (2020b); Lang et al. (2000); Lenka Thamae and Pottinger (2006); Transparency International (2007)
Nathpa Jhakri	India	1993	2004	Salini Impregilo	1′530	EJAtlas (2015a); Himdhara (2015); Lang et al. (2000)
Xiaolangdi	China	1994	2000	Impregilo	1′836	Lang et al. (2000)
Lower Kihansi	Tanzania	1995	2000	Impregilo	300	EJAtlas (2017e); International Rivers (2001); Lang et al. (2000); Quinn et al. (2005)
Ghazi Barotha	Pakistan	1996	2003	Impregilo	1′450	ILO (2002); Lang et al. (2000)
Gibe I	Ethiopia	1997	2003	Salini Costruttori	184	Carr (2017)
Kali Gandaki	Nepal	1997	2002	Impregilo	144	EJAtlas (2014b); Khadka (2003); Thanju (2008)
Caruachi	Venezuela	1997	1998	Impregilo	2′076	Lang et al. (2000)
Tokwe Mukorsi	Zimbabwe	1998	2017	Salini Impregilo	12	EJAtlas (2017c); Human Rights Watch (2015)
Tocoma	Venezuela	2002	**	Impregilo	2300	El Pitazo (2019); Poliszuk et al. (2018); Transparencia Venezuela (2018)
Kárahnjúkar	Iceland	2003	2009	Impregilo	690	EJAtlas (2014c); Thorarins (2013); Zhang (2013)
Gibe III	Ethiopia	2006	2016	Salini Impregilo	1′870	ARWG (2009); Carr (2017); EJAtlas (2020a); Franchi and Manes (2016); Hodbod et al. (2019); Human Rights Watch (2014a); OECD Watch (2017); The Oakland Institute (2019); Survival International (2021)
Bujagali	Uganda	2007	2012	Salini	250	EJAtlas (2015b); NAPE Uganda (2014)
HidroSogamoso	Colombia	2009	2015	Impregilo	820	EJAtlas (2019f); Moreno Socha (2019); Rios Vivos Colombia (2021); Roa Avendaño and Duarte Abadía (2012)
El Quimbo	Colombia	2010	2015	Impregilo	400	EJAtlas (2019a); Dussán Calderón (2021); Galindo Vanegas (2018)
Angostura	Chile	2010	2014	***	316	Ecosistemas (2018); EJAtlas (2017f); ElDesconcierto (2014); Osses (2014)
Grand Ethiopian Renaissance	Ethiopia	2011	2020	Salini Impregilo	6′000	BBC (2020); EJAtlas (2017a); Hussein (2014); International Rivers (2013, 2014, 2017); Roussi (2020); Zane (2020); Zelalem (2020)
Neckartal	Namibia	2013	2018	Salini Impregilo	3	Namibian Sun (2015); New Era Live (2014); Tjihenuna (2014)
Nenskra	Georgia	2015	*,**	Salini Impregilo	280	BankWatch (2019); Chipashvili (2017); DFWatch (2020); EJAtlas (2018)
Rogun	Tajikistan	2016	**	Salini Impregilo	3′600	EJAtlas (2016b); Human Rights Watch (2014b); Skoba (2013)

*In 2019, Salini Impregilo withdraws

**The facility is still under completion

*** Missing data

**Table 5:** Codebook for unsustainability claims associated with the analyzed hydropower schemes

Code	Emblematic cases	Illustrative quote
Design-construction defects		
Geological vulnerability of the site	Mosul, Daule Peripa, Nenskra	“Mosul dam engineers warn it could fail at any time, killing 1 m people” (Borger, 2016)
High costs of maintenance	Mosul, Chixoy, Tarbela	“Constant grouting is necessary to keep the structure from collapsing in upon itself” (Bender, 2014)
Over/under-sizing	GERD, Ertan, El Cajón, Nathpa Jhakri	“The dam is 300% over-sized. More than half of the turbines will be rarely used” (International Rivers, 2013)
Poor or none feasibility and alternatives' study	Bujagali, LHWP, Gibe III, El Quimbo	“Options like solar, wind, biomass and geothermal have not been adequately studied to provide evidence that Bujagali dam project is the least-cost option” (NAPE Uganda, 2014, p.19)
Structural and components' defects	Akosombo, Kariba, Chixoy	“Three turbines had to be taken out of service in 1998 at a cost of $5 million in lost production when cracks appeared” (Lang et al., 2000, on *Yacyreta*)
Financial unsustainability		“The dam has turned out to be a financial disaster” (Colajacomo, 1999, p.14)
Electricity overproduction	Ertan, Yacyreta	“The general manager of Ertan Hydropower Development Corporation, has become increasingly anxious because he could sell only 60% of the dam’s output” (Lang et al., 2000, on *Ertan*)
Increase of energy tariffs and public debt	Yacyreta, Bujagali, Daule Peripa	“ANDE wants to increase electricity tariffs by 30% to overcome its critical financial state” (Lang et al., 2000, on *Yacyreta*)
Project Cost overrun	Yacyreta, Tarbela, Bujagali, Chixoy	“The dam’s costs soared from an original estimate of $2.7 billion to $11.5 billion” (Lang et al., 2000, on *Yacyreta*)
Time overrun	Bumbuna, Tocoma, Yacyreta	“‘When Bumbuna is completed’ became a popular phrase, which indicated, ‘never’” (Mazzei & Scuppa, 2006, p. 15)
Geopolitical and interstate conflicts	GERD, Mosul, LHWP, Bumbuna	“Ethiopia has started filling the GERD, as Egypt still calls it an ‘existential threat’” (Roussi, 2020)
Impacts—environmental disruption		
Emission of greenhouse gases	Hidrosogamoso, GERD	“Flooding 168,000 hectares will result in decomposition of vegetation, leading to emissions of carbon dioxide and methane gases” (Luna, 2020)
Biodiversity loss	Kihansi, LHWP, HidroSogamoso	“The loss of the spray from the waterfall […] has sent the critically endangered Kihansi Spray Toad and at least two endangered plant species to the brink of extinction” (International Rivers, 2001)
Deforestation	GERD, El Quimbo, Kali Gandaki	“The dam will flood 1,680 square kilometers of forest” (International Rivers, 2014)
Disruption of river ecology and water contamination	Gibe III, Karahnjukar, Kariba	“The reduction in river flow will cause the level of Lake Turkana to fall by about two thirds.” (Survival International, 2021)
Erosion and sedimentation	Akosombo, Chivor, Karahnjukar	“The trapping of silt behind the dam has also led to severe coastal erosion downstream, with beaches and sections of the highway along the West African coast being washed away” (Lang et al., 2000, on *Akosombo*)
Loss of protected, sacred or archeological sites	Xiaolangdi, Gibe III, El Quimbo	“The reservoir will also flood 100 archeological sites where 10,000 year-old relics, and objects from the Song Dynasty (900–1279 AD), have been found” (Lang et al., 2000, on *Xiaolangdi*)
Impacts—socio-economic disruption		
Accidents	Kainji, Kariba	“At least 39 people were killed […] after floodgates were opened to release rising floodwaters at the Kainji dam” (Lang et al., 2000, on *Kainji*)
Displacement & Resettlement		
Forced or violent displacement	Chixoy, Gibe III, Akosombo, Tokwe Mukorsi	“A massacre took place in Guatemala that left 400 people dead. Countless more were displaced, tortured, raped or left starving. And all to make way for a hydroelectric dam” (Dearden, 2012)
Inadequate or no compensation measures	Tarbela, Bujagali, Chixoy, Tokwe Mukorsi, Gibe III	“Some 96,000 people were displaced by the project and are still fighting in the courts for compensation” (Lang et al., 2000, on *Tarbela*)
Inadequate resettlement	Kariba, Bujagali, LHWP, Rogun	The involuntary resettlement of 57,000 people within the reservoir basin and immediately downstream from the dam was responsible for serious environmental degradation which was one of a number of factors that left a majority of those resettled impoverished (Scudder, 2005, p. 1)
Health related issues	Akosombo, Chivor, Daule Peripa, Lower Kihansi, Kpong	“Water-borne diseases such as schistosomiasis, onchocerciasis and malaria have increased dramatically since the filling of the reservoir” (Lang et al., 2000, on *Akosombo*)
Increase in violence and crime	El Quimbo, Gibe III, LHWP	“Now that the lake has reduced, the other tribes have moved closer and raids have intensified along with killings on both sides.” (Allibhai, 2015, p. 15)
Local poverty creation		
Damage or loss of properties and households	Bujagali, LHWP, Hidrosogamoso, Kariba, Rogun, Tokwe Mukorsi	“Households from both sides of the river banks […] raised concerns about damages to their houses due to blasting of rocks at the dam site.” (NAPE Uganda, 2014, p.15)
Energy Poverty	Yacyreta, Bujagali, Rogun	“The government provides electricity to resettled communities for only a few hours per day.” (Human Rights Watch, 2014b, p.32)
Loss of basic facilities and services	Katse, Chivor, Kossou	“Getting sick is practically forbidden in that sector during the summer. Without river transportation, transporting a patient to the municipality's health center becomes a difficult and very expensive odyssey.” (Semana, 2019)
Loss of livelihoods and employment	Gibe III, Tokwe Mukorsi, Kariba	“the dam is set to destroy the livelihoods of hundreds of thousands of tribal people” (OECD Watch, 2017)
Malnutrition and lack of safe water	Bujagali, Daule Peripa, Tokwe Mukorsi, Kariba	“Between 70 and 90% of inhabitants do not have access to drinkable water” (Gerebizza, 2009, p. 13)
Monetary poverty	Rogun, Towke Mukorsi, Chixoy, Hidrosogamoso	“People who had previously relied on their lands to provide food reported that, after resettlement, they had to purchase most or all of their food at markets, leaving less money for other household needs.” (Human Rights Watch, 2014b, p.3)
Loss or threat to cultural identity	Bujagali, Angostura, Tarbela, Gibe III	“We had our own culture and customs. We had a set way of life. All that has been disturbed” (Bennet & McDowell, 2012, p. 41)
Migration	Hidrosogamoso, Kossou	“As their fields produce too little, the young people are forced to look for jobs abroad” (Prowizur, 1976, p. 244)
Labor rights violation and safety issues		
Accidents & deaths at construction site	Mosul, Kariba, Bujagali, Neckartal	“The Iraqi-American hydrological engineer, told me that, in Iraq, when laborers fell into wet cement during large infrastructure projects, it was common for the work to carry on.” (Filkins, 2016)
Low or inadequate wages	Ghazi Barotha, Bujagali, GERD, Rogun, Neckartal	“Impregilo is accused by local and international trade unions of not respecting a joint agreement on wage” (Lang et al., 2000, on *Ghazi Barotha*)
Poor or unsafe working conditions	Neckartal, Ghazi Barotha, Mosul	“They work on empty stomachs with no safe drinking water at the construction site despite the long extended working hours” (Tijhenuna, 2014)
Threats and Violation of constitutional workers' rights	Neckartal, Nathpa Jhakri, Hidrosogamoso	“Workers expressed grievances over allegations of discrimination, human rights violations, victimization, racism, and unfair dismissal, among a host of other things.” (Tijhenuna, 2014)
Repression of dissent		
Assassinations of environment defenders	Hidrosogamoso, Chixoy	“Assassination of six members of the Social Movement in defense of Sogamoso river” (censat.org)
Criminalization of dissent	El Quimbo, Chixoy, Ghazi Barotha	“Union leaders’ relatives were detained and some even tortured.” (Lang et al., 2000, on *Ghazi Barotha*)
Violent repressive measures and militarization	Chixoy, Bumbuna, Gibe III, Kariba, Tokwe Mukorsi, Hidrosogamoso	“The construction company's directors therefore decided to hire mercenary troops to protect their staff and prevent further theft and destruction of equipment and machinery” (D'Angelo, 2014, p. 39)
Lack of transparency		
Lack or poor information or involvement of local communities in the decision-making	Bumbuna, Tarbela, Gibe III, Chixoy, Daule Peripa, El Quimbo, GERD	“Consultations took place only in 1976, after the dam construction had started.” (Colajacomo, 1999, p. 2)
Alleged corruption and pending cases	Yacyreta, Bumbuna, Nenskra, Tocoma, James Bay, El Quimbo	“The Yacyreta dam was famously described by Argentinian president Carlos Menem as a ‘monument to corruption’” (Lang et al., 2000, on *Yacyreta*)
Proven corruption	LHWP	“Impregilo […] pleaded guilty to ‘attempting to defeat the course of justice’” (Transparency International, 2007, p. 89)
Contract- and bidding related issues	Gibe I and III, El Quimbo, GERD, Mosul	“EEPCO repeated its turnkey contracting—disregard for bidding process and project oversight—in successive Gibe dam contracts with Salini.” (Carr, 2017, p.32)
Poor or no E(S)IA	Gibe I and III, Chixoy, El Quimbo, LHWP, Bujagali	“The Ethiopian EPA did not produce an environmental or socio-economic impact report (EIA) prior to the development” (Carr, 2017, p.33)

**Fig. 2:**
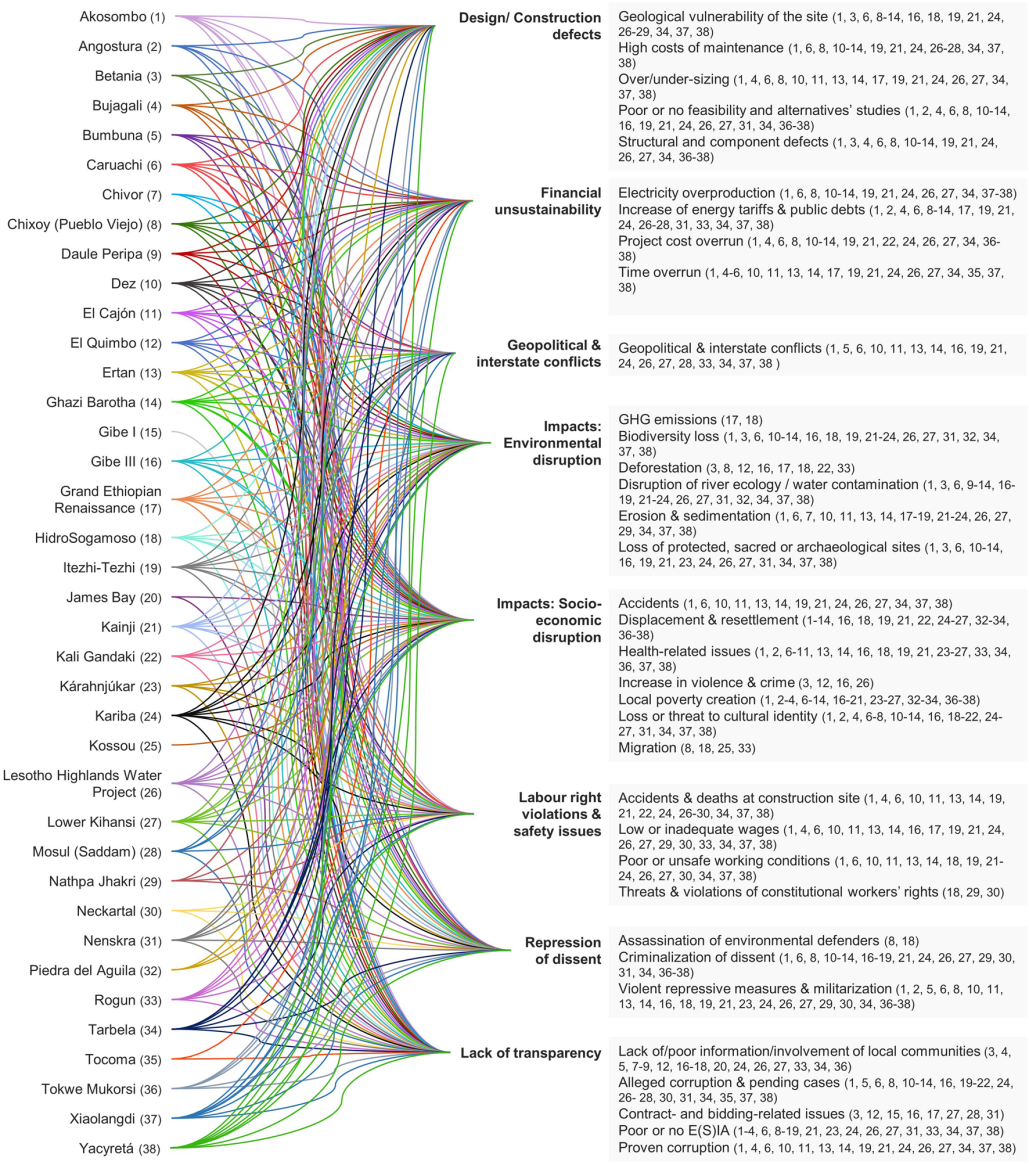
Matching cases with categories of unsustainability claims

**Fig. 3:**
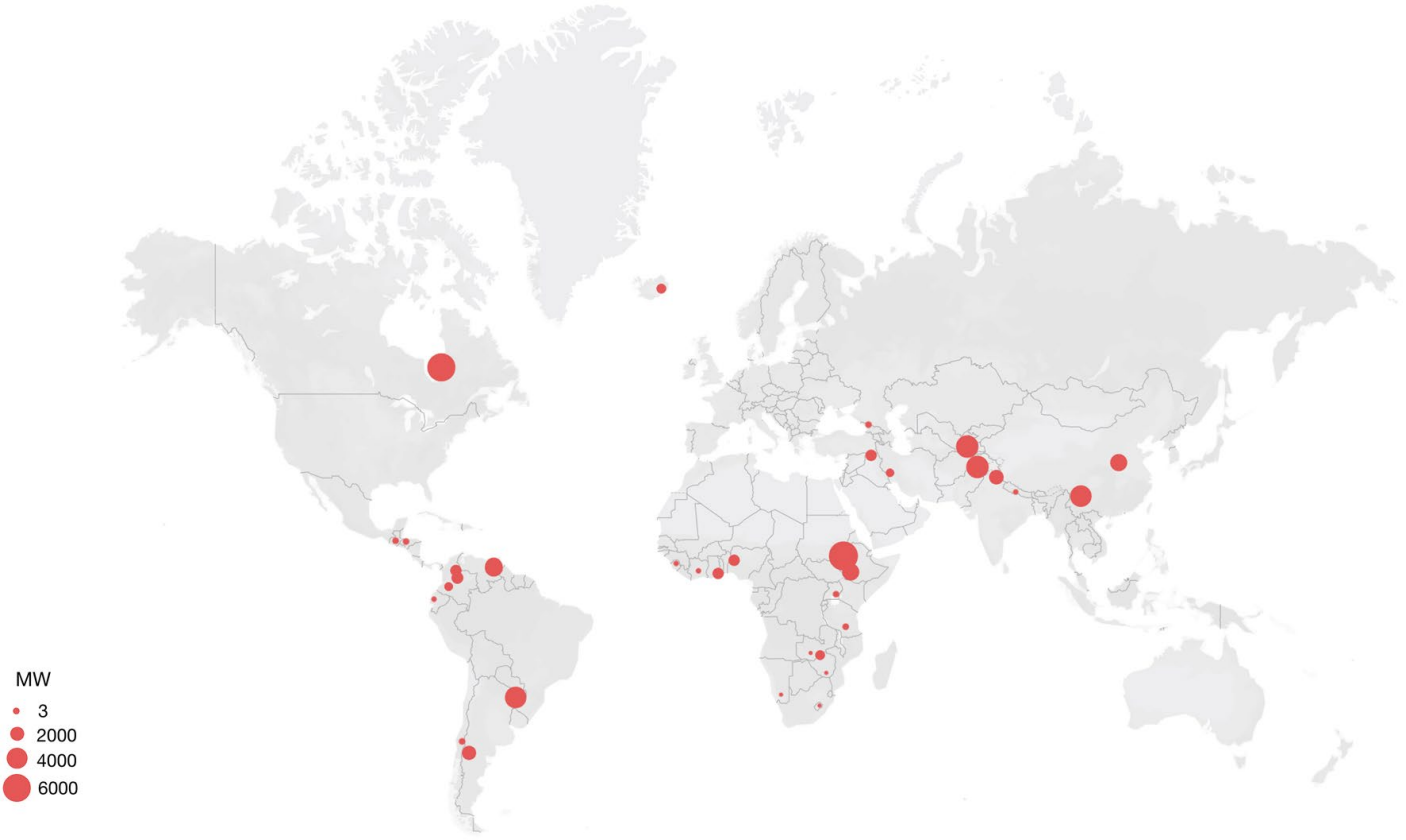
Geographical distribution of the analyzed cases. Each dot represents a hydropower scheme; the bigger the dot, the higher its generating capacity

Evidence of conflicts is retrievable across years, dam features, project phases and geographies. From the oldest to the most recent, the cases were ordered chronologically in Table 4 to show how disputes and conflicts are not bounded to a particular time, but rather recurring across decades. Controversies do not only emerge in the context of large hydropower development, as the power capacity ranges significantly across cases. The unsustainability claims tied to the dams encompass all stages of the civil works: from the project (technical-economical) design to its long term (environmental, social, economic) consequences, passing by the construction phase. The range of claims associated with multiple stages of each project make it hard to disentangle those specifically associated with civil works from broader claims which cannot be directly traced to WeBuild. This is particularly true for cases of geopolitical conflicts or allegations of corruption. Rather than excluding those broader and more ambiguous claims, we keep their evidence, as it points to the complex picture of which WeBuild is often a part of, and from which it cannot be divided. Figure 2 shows how different unsustainability claims are tied to the 38 dams. They are grouped under eight categories: design and construction defects; financial unsustainability; geopolitical and interstate conflicts; impacts: environmental disruption; impacts: socio-economic disruption; labor right violations and safety issues; repression of dissent; lack of transparency. Each category is then disaggregated into specific sub-nodes. As Fig. 2 shows, most dams map onto most broad categories: injustices are multi-faceted, so that one instance rarely occurs within an isolate category. When isolated cases do occur, it is important to note that this may also be due to lack of available evidence. On the right side of the figure, specific dams (identified by a number) are tied to unsustainability claims. Figure 3, on the other hand, shows the geographical distribution of the 38 dams. The figure shows a clear trend: except for Iceland’s *Kárahnjúkar* dam and Canada’s *James Bay* project, all the dams are in the Global South.

While the general overview of Fig. 2 provides a snapshot of the type of unsustainability claims tied to each dam, and Fig. 3 shows geographic trends, it is necessary to move to a case-by-case discussion to fully grasp the magnitude of the controversies as stake. Below, we touch upon some of the most emblematic cases from the database, moving through chronological and geographical order.

The *Kariba* dam on the border between Zambia and Zimbabwe, the oldest project in our database (with construction starting in 1956), is a historical example of forced displacement and of how dams can impoverish communities for decades. The descendants of the 57,000 Tonga people who were forcibly resettled for the construction of the dam still struggle today with hunger, alcoholism, prostitution and smuggling, among other problems (EJAtlas, 2017d; Scudder, 2005). A few years later in Ghana, the *Akosombo* dam (1961–1965) became notorious not only for its size (being one of the biggest reservoirs in the world), but also for systematic technical failures (e.g., several problems at the turbines), under-performance (e.g., 20 h per week blackouts during the 1994 drought) and downstream erosion (the coasts of neighboring Togo and Benin being lost at the rate of 10 m a year) (Lang et al., 2000). The dam, built to provide electricity to aluminum smelters and thus to boost the country’s industrialization, displaced 80,000 farmers and caused the spread of diseases such as malaria and river blindness (*Onchocerciasis*; *ibid*.; EJAtlas, 2016a).

In the 1980s, the company built some of the most controversial dams in the world, such as *Chixoy* (Guatemala), *Mosul* (Iraq), and *Yacyretá* (Argentina-Paraguay). In Guatemala, community members from Rio Negro opposed relocation and sought better compensation for the construction of the *Chixoy* dam, promoted by the Inter-American Development Bank, the World Bank and assigned to the then named Cogefar-Impresit company. Dam authorities, the army and the paramilitary labeled local Mayan indigenous communities as *guerrilleros* and perpetrated multiple massacres to curb the alleged anti-state threat. Estimates count about 400 people murdered, and many more tortured and violated, the majority being women and children (facts later known as the Rio Negro massacres; Colajacomo, 1999; EJAtlas, 2019b; GHRC, 2011; Lang et al., 2000; Manes, 2012). The few survivors had to escape to the hills and live for several years hiding in the woods. This way, the area was evacuated, and the building work could carry on. In 1999, the *Comisión para el Esclarecimiento Histórico* of Guatemala (promoted by the UN) acknowledged that genocide was perpetrated in Rio Negro. In 2005, the infamous case was brought to the Inter-American Commission of Human Rights. The same decade, the *Mosul* dam was built in Iraq under the rule of Saddam Hussein. The regime allegedly sought the multipurpose facility for downstream irrigation, flood control, and hydropower. However, in a country at war, the dam was mainly meant for the generation of electricity for war and to prevent the flooding of troops. Since its inception, the dam wall has been at risk of failure for a severe foundation defect, as it stands on a karstification-prone terrain. The dam has required constant maintenance to avoid a collapse that would affect more than six million people downstream (Al-Ansari et al., 2020; EJAtlas, 2017b; Filkins, 2016). The US Corps of Engineers called the Mosul dam “the most dangerous dam in the world” (Filkins, 2016). The transboundary *Yacyretá* dam between Argentina and Paraguay was called by Argentina's former president Carlos Menem “a monument to corruption” (EJAtlas, 2019e; Lang et al., 2000). Originally budgeted at $2.5 billion, the project's total cost has exceeded $15 billion, while it has operated at a maximum of 60% of capacity (*ibid*.). Its construction began in 1979, but the floodgates were closed, and the dam was filled for the first time only in 1994, before a detailed environmental and social mitigation plan was in place (*ibid.*). Meanwhile, thousands of the 50,000 people who were forced to move received no compensation (*ibid.*).

Researchers also raised concerns around projects built in the ‘90 s, a decade where big dam projects started to be heavily criticized by the public opinion. Those years in Asia, the company was contracted for the construction of the *Nathpa Jhakri* dam in India, *Xiaolangdi* in China, and *Ghazi Barotha* in Pakistan. In the Indian Himalayas, the *Nathpa Jhakri* dam is located in a flood prone zone. In August 2000, flash floods led to an extensive loss of time and money. To ensure continued support by the World Bank, employees worked round the clock to complete all restoration works to the pre-flood level. In 1999 workers at the plant denounced low wages and unfair working conditions. The mobilization received attention at the national level, also due to the repression measures taken by the police (EJAtlas, 2015a). The *Xiaolangdi* dam in China led to the eviction of 180,000 people and further impacts on at least 300,000. The reservoir also flooded 100 archeological sites with 10,000 years-old relics (Lang et al., 2000). In the case of the *Ghazi Barotha* dam in Pakistan, in the late ‘90 s, the Italian contractor was accused of not respecting an agreement on wage and working conditions with management and security forces physically abusing the workers, and arbitrary detentions of union leaders’ relatives. Trade unions denounced the suspension of their rights by the Government of Pakistan under the pressure of the project’s contractors (ILO, 2002). In the case of the *Lesotho Highland Water Project* and the related construction of the *Katse* and *Mohale* dams (1991–1997), the company was directly involved in a corruption case. According to the organization Transparency International, “Impregilo had restructured itself, arguably, in an attempt to avoid prosecution during an investigation. The company sought unsuccessfully to avoid trial by a number of artful arguments about the serving of the summons, the personal liability of employees for actions taken during the course of their employment and the jurisdiction of the court” (Transparency International, 2007: p. 89). Eventually, in 2008, the Lesotho High Court fined Impregilo US$ 2.04 million after it pleaded guilty.

Cases of highly intense socio-environmental conflicts are also reported at the dawn of the XXI century, despite the lull of the sector after the publication of the World Commission of Dams report (WCD, 2000). In Zimbabwe, fifty years after its first proposal, the *Tokwe Mukorsi* dam was completed in 2017 (EJAtlas, 2017c). According to Human Rights Watch, construction happened under a veil of corruption, stepping on the human rights of about 20,000 people whose home, land and livelihoods were taken. The Zimbabwe Government used the inexistence of compensatory mechanisms, inadequate food, shelter, sanitation, right to choose residence, misuse of humanitarian aid, coercion, force, harassment, and arrests to manage the development scheme behind the largest dam in the country (Human Rights Watch, 2015). In Ethiopia, besides the *Gibe III* project, it is worth mentioning the 300% over-sized *Grand Ethiopian Renaissance* dam, which echoes in the media as it is at the center of a heated dispute with downstream Sudan and Egypt for the control of the flooding of the Nile River (BBC, 2020; EJAtlas, 2017a; Hussein, 2014; International Rivers, 2013, 2014, 2017; Roussi, 2020; Zelalem, 2020; Zane, 2020). In Namibia, workers at the *Neckartal* dam construction site denounced extremely poor working conditions, abuse and victimization by their Italian supervisors (Namibian Sun, 2015; New Era Live, 2014; Tijhenuna, 2014). In Colombia, the *Hidrosogamoso* dam fueled organized protests by local communities. These protests have been systematically and violently repressed by military forces, with communities facing forced displacement, a grab of their sources of livelihood, militarization of the area and misrecognition of the status of affected people (EJAtlas, 2019f; Moreno Socha, 2019; Rios Vivos Colombia, 2021). The *El Quimbo* project is another highly controversial case in the country, where opponents have faced criminalization. One of the social leaders of the association* Asoquimbo* has been involved in five lawsuits (in all of which he was found innocent) for his activism against the project. He also denounced Impregilo for illegal practices in the extraction of construction materials (EJAtlas, 2019a; Dussán Calderón, 2021).

## Discussion

Our empirical exercise points to a mismatch of narratives. On one side, there is a corporation that has been internationally involved in the construction of large dams in the Global South for decades. The company claims it fully complies with international CSR standards, bringing prosperity to people and environments. On the other side, a radically different picture emerges when inspecting each case through multiple sources. Results show how some of the major dams tied to Salini Impregilo-WeBuild raise important environmental justice concerns, as they cause socio-ecological conflicts and produce negative social-economic-environmental impacts. The CSR guidelines and standards shown in Table 2 appear to be problematic when inspected through the lenses of third sources.

The inconsistency between what the company reports and what emerges from our data raises concerns around the voluntary and legally non-binding nature of CSR accounting. In fact, as the CSR mechanisms adopted by Salini Impregilo-WeBuild are based on self-reporting, there is no room nor any obligation for presenting third-party accounting. Given that the very nature of TNCs is to pursue the growth imperative in a competitive international market, every means becomes essential to meet such imperative, whether it implies looking away from high-level controversies tied to a firm’s operations, or systematically diverting the discussion toward win–win solutions in its corporate rhetoric. We argue therefore for the necessity of strengthening control over corporate activity beyond their own voluntary initiatives. In this perspective, our concerns also resonate with those of several social and environmental organizations regarding initiatives currently under debate at the European level, such as the Due Diligence Directive that should introduce EU-wide mandatory human rights due diligence requirements for businesses (see for example: DCP, 2021). This is an important step in the EU context. However, the lack of binding rules and control mechanisms might jeopardize the effective implementation of human rights and true social and environmental justice.

In this context, we question the reliability of existing CSR mechanisms and instruments as the main or only measure of a firm’s ethics. For example, WeBuild’s presence on the UN Global Compact website (Human Rights Watch, 2014b) is at odds with reports on violation of human rights and of unjust resettlement which appear throughout the company’s works (see Fig. 2). Similarly, the registrar and classification society DNV GL has accredited Salini Impregilo-WeBuild as complying with ISO 14001 standards (related to minimized environmental impacts). It is unclear whether the DNV GL is simply unaware of the multiple cases of environmental degradation caused by the corporation (as shown, again, in Fig. 2), or whether the lack of a standardized process to include these kinds of sources allows international societies to look away from such cases. As another example, the company Reconta Ernst & Young SpA, when auditing Salini Impregilo’s 2015a, 2015b sustainability report, concluded that the corporation was in compliance with Global Reporting Initiative standards. However, our results show how the under-reporting of all negative socio-environmental impacts of the corporation’s actions is severe, to say the least.

The stark differences between third-party reporting and apparent compliance with international standards shows how if the discussion on sustainability averts complexity, thus averting to challenge the broader political and economic systems in which any development project is promoted, sustainability accounting becomes a mere legitimization of business as usual. As such, it becomes an instrument of power through which the company tries to avoid conflict. In sustainability reports there is no space for acknowledging and responding to critical reporting, and the distinctive character of corporate publications is generally one of reduction of complexity (as in Boiral, 2013, 2016; Hahn & Lulfs, 2014; Talbot & Boiral, 2018). The absence of an independent third-party ensuring fair accountability allows corporations to construct their own version of facts (Laufer, 2003), while the difficulty in building and enforcing international monitoring instruments and mechanisms reinforces the limited liability of TNCs (De Jonge, 2011).

In this sense, the critical scholarships of post-normal science and political ecology help in both including complexity into the analysis and politicizing the debate around CS(I)R. By including multiple voices and sources of information, we aimed to respond to the call of post-normal science scholarship to embrace plurality and avoid the hegemonization of dominant narratives, while also improving the quality of the scientific research process in the context of socio-environmental conflicts. This provides a more nuanced picture of the complex socio-economic interests and impacts of conflictive and extractivist activities such as dam building, while problematizing the corporate one–way discourse on the sustainability of its own operations. We hold that the post-normal science philosophy becomes very relevant in the context of ecological conflicts globally, and in the analysis of CS(I)R.

As political ecologists, we argue for a re-politicization of the debate around CS(I)R that can lead governments and international institutions to act upon violations of human rights and environmental justice principles enshrined in international agreements and national constitutions. These include the unjust burden of socio-environmental impacts, the exclusion of local populations from participation in decision-making regarding their own needs and desires and the lack of due public hearings and accessible information about the projects. Eventually, they include the recognition of different and differing values, priorities, and languages of valuation of local people from those of a supposed development and progress for all touted by the corporation (Martinez-Alier, 2003, 2009).

This is particularly true for some regions. Scholars in post-development, post-colonialism, and post-extractivism studies (Acosta, 2011; Escobar, 1995; Machado Aráoz, 2007; Mbembe, 2019; Sachs, 1992; Shrivastava & Kothari, 2012; Zibechi, 2016) argue that the Global South has been long used either as a source of primary sources (including energy, food, and materials) or as a source of capital in the growth-oriented global economy, while communities and local value systems have been systematically dismissed. At the same time, real impacts on local communities and ecologies have been considered acceptable side effects in sacrifice zones. Business ethics and management studies could therefore importantly benefit from literature that discusses what ‘development’ really means in these contexts, whether large infrastructures are actually beneficial to local people, and how different values and world visions can truly be recognized as environmental justice principles.

In terms of methodology, we hope that the EJAtlas database becomes increasingly implemented as a repository of evidence that can further inform corporate analysis. The review process of conflictive cases presented in this article and in the EJAtlas can provide a toolbox for business ethics studies to inquire into environmental justice concerns beyond a single case study approach. In fact, the EJAtlas database contains information on the actors involved in the conflicts, including companies, for each conflictive case. As such, it can be used as a starting point to focus on injustices tied to specific companies. A comparative or statistical political ecology perspective could indeed offer valuable insights to investigate systematic patterns and evidence of irresponsibility and associated corporate discourses, how corporations operate around the globe, how they allocate their investments, and what local responses they face (Del Bene et al., 2018; Scheidel et al., 2020).

With respect to the lack of counter-reporting regarding corporate operations in business ethics studies, we suggest that scientists should use their own privileged role to unveil the power relations between different actors involved in CS(I)R accounting practices and of highlighting the incommensurable values people hold in connection to their territories and cultural systems. This shifts the role of scientists from one of truth-making to one of revealing the unavoidable complexity and plurality of the world. Our suggestion is that, when facing evidence of socio-environmental conflicts and injustices and while trying to quantify or measure CS(I)R, academia’s focus in discussing CS(I)R in the context of development projects should be placed in amplifying the voices of those who are on the ground, by mobilizing knowledge that is co-produced between academia, environmental activists and defenders (Temper & Del Bene, 2016). While we recognize that scientists themselves are also part of a complex web of power relations, a push in this direction could be given by spending time and resources to amplify the voices of those who have less power, engaging with those affected by environmental injustices on the ground and using diverse channels to problematize the way companies account for sustainability. The EJAtlas is one possible platform that can be concretely used to this aim.

## Conclusions

This paper collected evidence of unsustainability claims tied to the Italian construction company WeBuild, generating a counter-reporting exercise grounded in environmental justice, political ecology, and post-normal science.

Our hybrid and novel methodology came with some limitations. First, the large number of cases does not allow for an in-depth case study approach. Second, for the same reason it would not be feasible to check the legal responsibility of the company for all the claims and concerns that arose around the projects. The fact that the company under scrutiny here is a construction company rather than a commissioning body or a plant operator adds complexity to this endeavor, as their formal responsibility is supposedly limited to the building works. We have pointed at the conflictive issues that have arisen around dam projects either before, during, or after construction but that are not featured in any sustainability accounting and are therefore neglected or downplayed. Further research could aim to further discuss the role played by construction companies within global unsustainability dynamics.

Despite those limitations, we believe that the combination of post-normal science, political ecology and environmental justice has much to offer to the field of business ethics. Our recommendation is to bring these fields into a common dialog. In doing so, both the scientific process and the achievement of justice would benefit. Post-normal science helps problematize the debate on ethics through the provision of useful concepts from complexity science, while environmental justice can add case studies to the political CSR literature. While post-normal science tends to be developed and applied in more theoretical terms, political CS(I)R and environmental justice can give concreteness to Funtowicz and Ravetz’ scholarship. In turn, business ethics helps to put the focus on the ethics of corporations, who are key actors in the complex panorama of global socio-ecologies.

Future research in the field could be directed not only to further unpack the complex relationships and power dynamics between actors in the CS(I)R arena, but also to explore the reasons why inconsistencies between their dialectics exist in the first place. This can not only call into question (asymmetrical) human rights and environmental justice issues, but also helps in understanding how and why TNCs systematically avoid the critical discussion on responsibility in their accounts. More firm-focused case studies using counter-accounting as a methodology from fields other than hydropower development could add food for thought and enrich the business ethics literature by answering the question of who is behaving unethically and, importantly, how that behavior could be regulated. Lastly, the political economy of the uneven global development pattern is also an important aspect to consider in business ethics studies. In fact, almost the totality of the analyzed conflictive hydropower schemes is located in countries that are generally labeled as ‘developing’ or ‘underdeveloped’. The company’s narrative reported in this article is one of optimistic trust about the fact that megaprojects will bring prosperity, development, and alleviation of poverty in these countries. However, data show that promises such as job creation, health improvements and education facilities, or access to electricity, were not always met and were generally limited to a short time frame.

The ethics of CS(I)R is a complex issue, that needs to be politicized for building justice around socio-environmental conflictive contexts. Justice claims call therefore for a much more plural political arena of actors, particularly including those affected by large-scale hydropower projects.

## Supplementary Information

Below is the link to the electronic supplementary material.

Supplementary file 1 (PDF 30 kb)

## Footnotes

1 Turano (2011).

2 While we acknowledge the three ‘classical’ dimensions of sustainability, that are most used in business reporting (economic, environmental, social), we also understand them as inter-linkable and overlapping (Lonzano and Huisingh, 2011).

3 We acknowledge that the two terms ‘accounting’ and ‘reporting’ have been sometimes used interchangeably although the former is probably more commonly in use in Accounting research. However, we chose in the article to use 'reporting' in order to stress the fact that our practice deals with matters that are by no means commensurable, or countable.

4 UNI (Ente Italiano di Normazione), EN (European Committee for Standardization) and ISO (International Organization for Standardization) are respectively Italian, European and International organizations for (voluntary) standardization. They develop and publish Standards.
