# Supplementary material for: Counter-reporting sustainability from the bottom up: the case of the construction company WeBuild and dam-related conflicts
Source: J Bus Ethics. 2021 Dec 3;182(1):7–32. doi: 10.1007/s10551-021-04946-6 (PMC9768011; doi:10.1007/s10551-021-04946-6)
Supplement: Supplementary file 1 — (PDF 30 kb) [file 10551_2021_4946_MOESM1_ESM.pdf]

CODEBOOK

for the Original Paper titled “Counter-Accounting Sustainability from the Bottom-Up: The Case of a Dam Builder”

by Antonio Bontempi · Daniela Del Bene · Louisa Jane Di Felice

Journal of Business Ethics, 2021. https://doi.org/10.1007/s10551-021-04946-6

Contents

Codification of Salini Impregilo’s sustainability narratives 1

Codebook for unsustainability claims associated with the analyzed hydropower schemes 14

References 107

Codification of Salini Impregilo’s sustainability narratives

Codes	Coded Text	Source
Economic sustainability		
Energy security	Energy security – Access to energy represents one of the major pillars for the development of society. Yet the current fossil fuel-based energy system leaves about 1.4 billion people around the world without access to electricity.	Salini Impregilo (2013)
GDP Growth (at country and local levels)	Salini Impregilo aims to develop infrastructure projects that act as catalysts for growth in the countries where we operate	Salini Impregilo (2013)
GDP Growth (at country and local levels)	average of 73% of spending placed with locally-based providers of goods and services.	Salini Impregilo (2013)
GDP Growth (at country and local levels)	In all supply contracts, counterparties are required to sign up to the principles of the Salini Impregilo Code of Ethics.	Salini Impregilo (2013)

GDP Growth (at country and local levels)	local procurement (73% of supply purchased locally)	Salini Impregilo (2013)
GDP Growth (at country and local levels)	In 2014 our links with local providers of goods and services remained robust, with an average of 58% of spending placed with locally-based suppliers.	Salini Impregilo (2014)
GDP Growth (at country and local levels)	In 2015, we maintained good relationships with local suppliers, spending 75% of our procurement budget (on average) locally. Additionally, our construction sites contracted 3,500 new suppliers in 2015, of which 89% were local.	Salini Impregilo (2015a)
Green economy	Demonstrating compatibility of economic growth and sustainability – Under the 'green economy' paradigm, economic growth and socio-environmental sustainability are viewed not as incompatible, but as mutually reinforcing; creating jobs and sustainable	Salini Impregilo (2013)
Infrastructure need	Achieving the Sustainable Development Goals (SDGs) endorsed by the United Nations, which recognise the role of infrastructure in supporting sustainable development.	Salini Impregilo (2015a)
Infrastructure need	Addressing the growing infrastructure needs of the countries in which we operate	Salini Impregilo (2015a)
Infrastructure need	Creating infrastructure allows to secure an ongoing supply of natural resources such as water	Salini Impregilo (2015a)
Infrastructure need	Our projects help to enhance accessibility and raise countries' economic potential through energy and transport infrastructure.	Salini Impregilo (2015a)
Infrastructure need	Robust infrastructure is the lifeblood of strong economies and societies, playing a major role in industrial, agricultural, rural and urban development.	Salini Impregilo (2015a)
Infrastructure need	Infrastructure contributes significantly to these challenges, as it represents the backbone supporting the economic and social activities of every community, and is an essential prerequisite for growth	Salini Impregilo (2016b)
Job creation	During the year, some 90% of our 31,000 directly-employed staff consisted of local personnel, mainly employed within our African and American continent operations.	Salini Impregilo (2013)
Job creation	Our communities continued to receive strong support for their economies, in terms of job creation (90% of total workforce hired locally),	Salini Impregilo (2013)
Job creation	The principal measure of Salini Impregilo's contribution to local economies is the number of jobs created in host countries.	Salini Impregilo (2013)
Job creation	In addition to the direct workforce, the construction industry makes regular use of subcontractors for certain activities, as well as other providers of services (including technicians, consultants, catering staff, etc.), which contribute significantly to the number of jobs created at the local level. In 2014 around 16,700 people were employed	Salini Impregilo (2014)

	by our subcontractors, and another 2,900 by related service providers, 74% of whom were hired locally.	
Job creation	Although management roles at operational sites are primarily filled by expatriate Italian employees,	Salini Impregilo (2013)
Job creation	In Ethiopia, our ongoing Gibe III project and GERDP (Grand Ethiopian Renaissance Dam Project) employ over 12,000 direct staff, 96% of whom are recruited from local communities.	Salini Impregilo (2013)
Job creation	some 90% of our 31,000 directly-employed staff consisted of local personnel, mainly employed within our African and American continent operations	Salini Impregilo (2013)
Job creation	We focus on engaging a local workforce in areas where projects are located,	Salini Impregilo (2015a)
Job creation	the Group is involved in training talented local people, in order to create an international management team capable of transferring project capabilities around the world.	Salini Impregilo (2013)
Job creation	With the provision of skills training, health surveillance and high employment standards, job creation plays an important role in enhancing the capabilities of local personnel, mainly in developing countries	Salini Impregilo (2013)
Job creation	In 2015, we began collaborating with Milan's Polytechnic, an outstanding academic partner for specialist training and integrating recent graduates into the professional world. We helped to adapt an existing Masters degree course, inaugurating the first edition of the "International Construction Management" first and second Level Masters in early 2016.	Salini Impregilo (2015a)
Job creation	In 2015, we launched the Salini Impregilo Learning Academy,	Salini Impregilo (2015a)
Job creation	In addition to maintaining high employment standards and running health initiatives, our sites provide extensive skills training programmes for the local workforce. In 2015, we provided nearly 874,600 training hours to our workers globally, which is equal to an average of approximately 37 hours per individual (22 hours in 2014)	Salini Impregilo (2015a)
Job creation	We engaged nearly 15,000 people to participate in the projects at the end of 2015, 97% of whom come from rural communities nearby	Salini Impregilo (2015a)
Job creation	In April we launched the first edition of the "Master's in International Construction Management"	Salini Impregilo (2016b)

Poverty alleviation	Poverty alleviation – Infrastructures improvement is inextricably linked with poverty alleviation, particularly in low-income countries, where better infrastructure can provide a safety net against natural disasters and economic shocks.	Salini Impregilo (2013)
Poverty alleviation	We aim to understand the needs of future stakeholders (e.g. farmers) and where possible, we arrange the handover of these areas to local farmers after restoration, ensuring a direct benefit for local communities.	Salini Impregilo (2013)
Revenues creation (for shareholders; from labor income)	Our shareholders and investors benefited from the growth of our revenue (+13.6%) and backlog (+9.9% in the construction and plant sector) in terms of return on their investments and share value (+108.57%);	Salini Impregilo (2013)
Revenues creation (for shareholders; from labor income)	The Company has operating procedures and practices designed to ensure that its remuneration policies comply with the regulations applicable in all the countries where the Group operates and especially the minimum wage requirements, where these exist.	Salini Impregilo (2017a)
Environmental sustainability		
Circular economy	improving performance throughout the infrastructure's life cycle.	Salini Impregilo (2016b)
Circular economy	The reuse and recycling of waste was increased by 10% compared	Salini Impregilo (2016b)
Clean, renewable energy	High consumption of fossil fuels is widely recognised as the primary cause of global warming, which in turn leads to increased unpredictable precipitation, rising sea levels, increased catastrophic flooding and the spread of pests and diseases once limited to the tropics.	Salini Impregilo (2013)
Clean, renewable energy	Salini Impregilo strives to reduce energy consumption and related GHG emissions	Salini Impregilo (2013)
Clean, renewable energy	through our projects we play an important role in combatting climate change	Salini Impregilo (2013)
Clean, renewable energy	Transition to a low carbon economy – Investment in the energy, water, transport and building sectors has been highlighted by the United Nations Environmental Programme (UNEP) as fundamental to achieving a low-carbon and resource-efficient future.	Salini Impregilo (2013)
Clean, renewable energy	through our projects we play an important role in combatting climate change. Our worldwide leadership in the water sector rests on a track record of 230 hydroelectric projects completed with an installed capacity of more than 37,200 MW.	Salini Impregilo (2014)
Clean, renewable energy	The International Energy Agency (IEA) forecasts that the installed hydroelectric capacity will continue to grow, reaching 2,000 GW by 2050, preventing the emission of 3 billion tonnes of CO2 a year from the use of fossil sources29 The hydropower sector can generate reliable power at a lower cost than other sources, thereby contributing to the	Salini Impregilo (2016b)

	economic development of recipient countries with consequent benefits in terms of business opportunities, job creation, and social welfare.	
Impact Assessment	Construction of motorways, bridges, dams and railway lines requires the use of large quantities of water, aggregates, iron, cement and backfill: all raw materials which are mostly not renewable. The environmental assessments made at the start of a new contract consider these aspects and the related mitigation measures are designed to ensure the efficient management of these resources and, when possible, the use of alternative materials without reducing the quality, performance, security and functioning of the asset.	Salini Impregilo (2017a)
Impacts mitigation	Each site plans and carries out training activities for all staff involved in operations with potential impacts on the environment, sub-contractors' personnel included.	Salini Impregilo (2013)
Impacts mitigation	Local impacts mitigation	Salini Impregilo (2013)
Impacts mitigation	We are also committed to reducing the direct impacts of our construction activities to a minimum.	Salini Impregilo (2013)
Impacts mitigation	We are committed to reclaiming all areas affected by our plants, facilities, quarries and landfills during or after the completion of a project. The aim is to leave these areas in a condition that facilitates natural re-vegetation, prevents soil erosion, improves slope stability, returning affected areas to their original state.	Salini Impregilo (2013)
Impacts mitigation	we saved about 4.9 million litres of diesel both through purchasing renewable energy and recovering on-site energy by using special energysaving devices, achieving associated reduction of GHG emissions of 13,192 tonnes of CO2 equivalent, equal to 16% of total direct and indirect emissions. Moreover we installed a water recycling system at our batching plant recovering industrial water for reuse. In the period we also reached the record level of recycled waste, equal to 68% and we reused all the rocks and soil excavated	Salini Impregilo (2013)
Impacts mitigation	During the construction phase, special protection measures are required when sites are adjacent to – or within – protected areas and/or if protected wild species are present.	Salini Impregilo (2013)
Impacts mitigation	Every infrastructure project has an impact on the landscape and local flora and fauna. It should therefore be designed to ensure full integration with the surrounding environment and preservation of local species	Salini Impregilo (2013)
Impacts mitigation	Flora and fauna are protected by careful management of water, operating areas, quarries and areas designated for the storage of materials.	Salini Impregilo (2013)
Impacts mitigation	In particular, the Group strictly prohibits the removal, damage or disturbance of natural habitats in its site areas, applying to the relevant authorities for any intervention	Salini Impregilo (2013)

Impacts mitigation	Where environmental impact assessments highlighted risks of interference with protected species and, more generally, with the biodiversity of the interested areas, technical arrangements have been implemented to mitigate the possible impact of works on the environment	Salini Impregilo (2013)
Impacts mitigation	Salini Impregilo consolidates its monitoring and mitigation efforts with a commitment to preserving and protecting biological diversity at its operating sites.	Salini Impregilo (2014)
Impacts mitigation	Prior to starting any activities we carry out an assessment of the potential impacts deriving from noise and vibration, determining which measures to adopt to safeguard workers' health and safety	Salini Impregilo (2014)
Impacts mitigation	We place great emphasis on rationalisation and efficiency in the use of raw materials	Salini Impregilo (2013)
Impacts mitigation	We place great emphasis on rationalisation and efficiency in the use of raw materials,	Salini Impregilo (2014)
Impacts mitigation	We are committed to reclaiming all areas affected by our plants, facilities, quarries and landfills during and after the completion of a project. The aim is to leave these areas in a condition that facilitates natural re-vegetation, prevents soil erosion, improves slope stability, thereby returning affected areas to their original state.	Salini Impregilo (2014)
Impacts mitigation	In 2013 we reforested about 626,000 m2 trees. We also restored topsoil on about 2,267,000 m2 protected over 4,767,000 m2 of soil from erosion	Salini Impregilo (2013)
Impacts mitigation	Salini Impregilo carefully monitors and protects its sites to prevent potential damage to the soil and subsoil.	Salini Impregilo (2013)
Impacts mitigation	Salini Impregilo carefully monitors and safeguards its sites against any potential harm to the soil and subsoil.	Salini Impregilo (2014)
Impacts mitigation	Waste management plans are set up at each site to manage waste in a proper manner, complying with all applicable regulations and best practices.	Salini Impregilo (2013)
Impacts mitigation	Waste management plans are set up at each site to manage waste in a proper manner, complying with all applicable regulations and best practices.	Salini Impregilo (2014)
Impacts mitigation	We are committed to ensuring at each site a close management of all phases of the water cycle: from the identification of water needs and supply sources to the realisation of water handling networks with related supply and discharge points, control plans and emergency response procedures.	Salini Impregilo (2013)
Impacts mitigation	We are committed to ensuring at each site a close management of all phases of the water cycle	Salini Impregilo (2014)
Social sustainability		
Anti-corruption policy	working to help advance the UN Global Compact's ten universal principles on human rights, labour practices, the environment and anti-corruption.	Salini Impregilo (2013)

Anti-corruption policy	Salini Impregilo has a zero tolerance policy for all types of corruption and is committed to complying with the anti-corruption laws ruling in all the countries where it operates.	Salini Impregilo (2017a)
Community support - capacity building	local road improvements, electricity supply, sport facilities construction and job opportunities.	Salini Impregilo (2013)
Community support - capacity building	social support initiatives (about 90 in the year);	Salini Impregilo (2013)
Community support - capacity building	For decades now, one of the mainstays of our everyday activities is to ensure full integration of our sites with the surrounding areas, paying close attention to three key features: a deep knowledge of our territories, constant engagement practices, and commitment towards our communities. Through this threefold approach we have been able to obtain not only strong local support, but also optimum results for all concerned.	Salini Impregilo (2014)
Community support - capacity building	During the execution of our projects, local communities can access some of our sites' facilities, such as on-site clinics, training rooms, wells, roads and bridges.	Salini Impregilo (2013)
Community support - capacity building	We carried out 47 interventions in this field. Of particular importance was the creation of a water supply line in Ethiopia that withdrew pure water from a fountainhead on high ground to serve a remote village and a health centre offering care to almost 200,000 people.	Salini Impregilo (2013)
Community support - capacity building	we have realized dozens of projects in recent years, including schools, health centres, public offices, water networks, roads and bridges – mainly in Sub-Saharan Africa.	Salini Impregilo (2013)
Community support - capacity building	As our track record shows, the Group's direct involvement in providing public infrastructure is the mainstay of our investment in local communities.	Salini Impregilo (2014)
Community support - capacity building	In certain cases, such as in rural areas not served by basic amenities, we offer local communities free access to some of our sites' facilities, such as on-site health clinics, training rooms, water wells, roads, and bridges.	Salini Impregilo (2014)
Community support - capacity building	in recent years we have realised dozens of enhancement projects, including schools, health centres, public offices, water networks, roads, and bridges – mainly in Sub-Saharan Africa.	Salini Impregilo (2014)
Community support - capacity building	A total of 183 social programmes were carried out in the 2012–2013 period	Salini Impregilo (2013)
Community support - capacity building	In Uganda for example, in partnership with Oncology for Africa, we established a house to welcome patients from rural villages requiring chemo/radiotherapy at Nsambya Hospital in Kampala. (For more details see the following box). Our Ethiopian site clinics also ensured over 12,000 free healthcare interventions for local communities.	Salini Impregilo (2013)

Community support - capacity building	supporting orphanages, charitable institutions and helping people in need of medical treatment and assisting communities.	Salini Impregilo (2013)
Community support - capacity building	We supported 15 educational initiatives around the world, comprising the provision of educational materials and the realisation and rehabilitation of schools' facilities including libraries, laboratories, computer and sports facilities.	Salini Impregilo (2013)
Community support - capacity building	A total of 207 social programmes were carried out over the period 2012-2014 (48 in 2014)	Salini Impregilo (2014)
Community support - capacity building	Each programme is based on a sound knowledge of the territory in question and the needs of the inhabitants, to ensure that energies are directed toward initiatives of genuine interest to communities.	Salini Impregilo (2014)
Community support - capacity building	In some countries, particularly in Africa, where the schools are limited in number and children and young people have difficulty accessing these, we build schools and provide assistance for their daily operation. In other countries, we support education programmes in partnership with other organisations and local institutions.	Salini Impregilo (2014)
Community support - capacity building	Projects located far from health centres offer free healthcare assistance to people from local communities	Salini Impregilo (2014)
Community support - capacity building	> 29,800 people hosted at our projects during about 640 open-door events	Salini Impregilo (2015a)
Community support - capacity building	Founded the E4Impact Foundation to promote social entrepreneurship in Africa<br>- Provided more than 17,000 free healthcare interventions from our clinics to local communities in remote, rural areas<br>- Hosted more than 29,000 people at our projects during open-doors events<br>- Engaged over 1 million people through our digital platforms	Salini Impregilo (2015a)
Community support - capacity building	In particular, the E4impact MBA supports participants in transforming a business idea into a business plan, improving existing businesses, creating partnerships with investors, suppliers and customers	Salini Impregilo (2015a)
Community support - capacity building	The Foundation also launched another programme, called First-Step Africa, aimed at supporting the expansion of foreign small-tomedium sized businesses (SMEs) in Sub-Saharan Africa through the E4Impact MBA	Salini Impregilo (2015a)
Community support - capacity building	We founded E4Impact Foundation together with Italian companies Securfin and Mapei, the Università Cattolica del Sacro Cuore of Milan (Europe's largest private university), and the Always Africa association. Our collective effort builds on a previous programme launched in 2010 by the ALTIS Graduate School Business and Society at the Università Cattolica.	Salini Impregilo (2015a)

Community support - capacity building	The Group contributes to developing the areas in which it works through initiatives to assist the local communities, which may include sponsorships, social and philanthropic initiatives.	Salini Impregilo (2017a)
Ethics	This commitment is clearly stated in the Group's Code of Ethics. The Code defines the values and principles of honesty, fairness, integrity, transparency, impartiality, confidentiality, safety of physical integrity and human dignity, protection of the environment, dialogue and engagement with stakeholders as the distinctive elements of the Group's activities	Salini Impregilo (2013)
Ethics	Code of Ethics, which defines for each corporate value the principles that guide our behaviours. These include honesty, fairness, integrity, impartiality, confidentiality, physical integrity protection, respect for human dignity, environmental protection, and the respect of local communities. The Code represents a reference point for our employees and everyone who contributes to our mission and objectives.	Salini Impregilo (2015a)
Human Rights (of local populations and workers)	working to help advance the UN Global Compact's ten universal principles on human rights, labour practices, the environment and anti-corruption.	Salini Impregilo (2013)
Human Rights (of local populations and workers)	To this end, in 2014 the Group signed an International Framework Agreement with both the Italian and the International Construction Unions aimed at promoting the fundamental principles of human rights worldwide, in line with declarations ratified by the United Nations, the International Labour Organisation, and the OECD.	Salini Impregilo (2014)
Human Rights (of local populations and workers)	In line with the provisions of the Guiding Related SDGs Principles on Business & Human Rights of the United Nations, in 2016 the Group conducted a complete mapping of the human rights impacts potentially arising from its activities.	Salini Impregilo (2016b)
Human Rights (of local populations and workers)	The Group supports the rights enshrined in the International Bill of Human Rights and the International Labour Organization conventions.	Salini Impregilo (2016b)
Human Rights (of local populations and workers)	Indigenous rights	Salini Impregilo (2017a)
Human Rights (of local populations and workers)	Salini Impregilo is committed to ensuring respect for the rights enshrined in the International Charter of Human Rights and the fundamental conventions of the International Labour Organisation in line with the principles established in its Code of Ethics and Sustainability Policy	Salini Impregilo (2017a)
Human Rights (of local populations and workers)	Salini Impregilo is committed to respecting the rights of communities directly affected by its operations.	Salini Impregilo (2016b)
Human Rights (of local populations and workers)	The main issues managed in this area concern the safety of the community (eg. public safety associated with the movement of worksite vehicles and hazardous activities,	Salini Impregilo (2016b)

	such as excavation by blasting), health (through the adoption of health protocols to mitigate the spread of diseases associated with the presence of the new workforce in the project's area), the environment (through the protection of natural resources used by communities, such as water, protecting soil from erosion, adopting timely accident management procedures), cultural and archaeological heritage (through the appropriate management of archaeological finds).	
Human Rights (of local populations and workers)	Indigenous rights	Salini Impregilo (2017a)
Human Rights (of local populations and workers)	Regardless of local rules on minimum working age, we only hire personnel worldwide over the age of eighteen years, verifying applicant documents at each site to prevent and detect any fraud.	Salini Impregilo (2013)
Human Rights (of local populations and workers)	We respect national and international law at all our sites and contracts in force ensure equal rights and fair treatment. Regulatory conditions for non-EU personnel are based on, and therefore similar to, those for EU personnel, with the key differences being that leaving indemnity is not provided in all countries, and taxes and contributions are linked to the region in which an individual is employed.	Salini Impregilo (2013)
Human Rights (of local populations and workers)	Where applicable, the minimum salary levels in all countries in which Salini Impregilo operates are rigorously respected. In 2013 entry-level wages exceeded local minimum wages by an average of 19%1	Salini Impregilo (2013)
Human Rights (of local populations and workers)	best practices such as non-discrimination, prohibition of child labour, adequate economic treatment, health and safety, respect for the environment, freedom of association and collective<br>bargaining;	Salini Impregilo (2014)
Human Rights (of local populations and workers)	Another important issue worth highlighting is the management of security, understood as the protection of corporate assets and staff safety.	Salini Impregilo (2016b)
ENR recognition	September – Salini Impregilo confirms its place as worldwide leader in the water sector in the ENR ranking	Salini Impregilo (2016b)
Environmental Sustainability	working to help advance the UN Global Compact's ten universal principles on human rights, labour practices, the environment and anti-corruption	Salini Impregilo (2013)
Impact Assessment	All potential environmental impacts deriving from our construction activities are assessed according to a standardised methodology, based on specific criteria (probability of occurrence, consequences for the environment, duration of the event, difficulty of restoration). Following the impact assessment, each project prepares an	Salini Impregilo (2013)

	Environmental Management Plan, which describes the management and monitoring activities (Environmental Control Plans) for all environmental components involved.	
Impact Assessment	All potential environmental impacts deriving from our construction activities are assessed according to a standardised methodology, based on specific criteria (probability of occurrence, consequences for the environment, duration of the event, difficulty of restoration). Following the impact assessment, each project prepares an Environmental Management Plan, which describes the management and monitoring activities (Environmental Control Plans) for all the environmental components involved.	Salini Impregilo (2014)
Impact Assessment	Assesses the potential impacts deriving from the project and identifies mitigation and compensation measures, also through public consultation with affected stakeholders and communities	Salini Impregilo (2016b)
Impact Assessment	Regularly monitor the respect of the approved ESIA's requirements during the project's implementation	Salini Impregilo (2016b)
Impact Assessment	The ESIA is usually part of the document attached to the contract concluded between the client and the contractor	Salini Impregilo (2016b)
Stakeholders engagement	Engagement activities include regular meetings on several issues – from updates on potential impacting activities (such as the use of local water sources), to understanding the community's needs to find appropriate solutions.	Salini Impregilo (2013)
Stakeholders engagement	Engagement with local stakeholders	Salini Impregilo (2013)
Stakeholders engagement	Meetings with community representatives, regular communications activities, sponsorships and social contributions activities	Salini Impregilo (2013)
Stakeholders engagement	the Group's approach towards local communities, which aims to ensure full integration of our sites with the surrounding area.	Salini Impregilo (2013)
Stakeholders engagement	We have built a strong reputation as an organization committed to respecting local cultures, needs and expectations;	Salini Impregilo (2013)
Stakeholders engagement	> 3,000 people engaged in over 190 meetings held with local communities and their representatives	Salini Impregilo (2015a)
Stakeholders engagement	At an operating level, our principal engagement activities relate to the features of a particular project. Our key stakeholders in this context include partners, employees, local communities, suppliers, contractors and sub-contractors, clients, local authorities, and organisations such as unions and NGOs (nongovernmental organisations).	Salini Impregilo (2015a)
Stakeholders engagement	For Salini Impregilo, building shared growth also means interacting with and supporting the communities that live near our sites. We have a longstanding commitment to	Salini Impregilo (2015a)

	understanding the cultures, needs, and expectations of those communities. For example, we seek to integrate our sites with the surrounding areas by deepening our knowledge of the country and local area, and regularly engaging with communities.	
Stakeholders engagement	In 2015 we did it engaging over 86,700 local stakeholders through meetings, information campaigns and opendoors events	Salini Impregilo (2015a)
Stakeholders engagement	Transparency is fundamental in managing relationships with the communities living near our sites. Our engagement with communities is guided by fairness and respect for local cultures.	Salini Impregilo (2015a)
Stakeholders engagement	Working with our on-site stakeholders closely every day has shown us the importance of dialogue in the success of any project. Transparency and engagement are therefore intrinsic features of our sustainability strategy.	Salini Impregilo (2015a)
Stakeholders engagement	>42,300 people hosted at our projects during 740 open-door events<br>>262,200 people involved in over 160 campaigns of information on our projects	Salini Impregilo (2016b)
Stakeholders engagement	>6,200 people involved in about 400 meetings with local communities and their representatives	Salini Impregilo (2016b)
Stakeholders engagement	Contractual agreements between Salini Impregilo and its clients require in most cases that relations with local communities be managed jointly, usually with a leadership role assumed by the client and a supporting role provided by Salini Impregilo. Based on these agreements, the communication channels to be used in the worksite are defined, be they physical (e.g. office for relations with the public) or technological, in the form of dedicated phone lines, websites, email addresses, etc. These channels allow the project to maintain a direct contact with local communities, providing information and gathering any feedback. The comments and feedback received are analysed by the project's management and addressed according to specific procedures (grievance mechanisms). Working continuously in co-operation with its clients, Salini Impregilo develops and implements comprehensive communication campaigns to inform the local population about the projects' progress, while also planning worksite visits, special events and social initiatives.	Salini Impregilo (2016b)
Stakeholders engagement	Meetings, presentations, focus groups, workshops, interviews, consultations, career days, public events	Salini Impregilo (2016b)
Stakeholders engagement	Transparency is therefore a key driver for the Group, accompanied by an approach to local communities guided by the principles of fairness and respect for local cultures and traditions.	Salini Impregilo (2016b)

Work safety and health	All our sites outside Italy have medical facilities that provide 24-hour medical advice, an ambulance service and a guarantee that each work shift has access to medical staff. All operating sites have a pharmacy, providing access to urgently-required medicines and those needed for the treatment of chronic diseases.	Salini Impregilo (2013)
Work safety and health	All personnel receive an induction on the Group's health and safety policies and procedures when they join, with additional training on specific risks in line with their assigned tasks.	Salini Impregilo (2013)
Work safety and health	investment in health and safety protection (+54%) and injury reduction (-30%);	Salini Impregilo (2013)
Work safety and health	Our efforts involve designing and building suitable camps to accommodate workers and their families, equipped with facilities including canteens, hospitals, fitness centres and athletics fields, markets, bars and internet connections.	Salini Impregilo (2013)
Work safety and health	Salini Impregilo has a company health service,	Salini Impregilo (2013)
Work safety and health	Salini Impregilo recognises the critical importance of occupational health and safety – and the protection of employees and third parties across all activities at its offices or operating sites. The Group's main objectives in this area are eliminating or reducing to a minimum the risk of accidents by adopting a prevention and protection system applicable to all operating activities, according to the principle that all accidents can be prevented	Salini Impregilo (2013)
Work safety and health	We pay particular attention to the provision of good living conditions for personnel employed in remote areas and challenging socioenvironmental contexts.	Salini Impregilo (2013)
Work safety and health	Through the adoption of the Safety Management System, certified according to the BS OHSAS 18001 standard, Salini Impregilo has achieved important results over the years, including the cultivation of safety awareness among its employees; a significant reduction in accidents at work; the prevention of occupational diseases; and a steep reduction of insurance costs and fines.	Salini Impregilo (2014)
Work safety and health	Reduced injury rate (IR) by 14%, compared to 2014 - Invested 34.8 € million in H&S protection and prevention - Delivered more than 637,000 hours of training to employees on H&S issues	Salini Impregilo (2015a)

Codebook for unsustainability claims associated with the analyzed hydropower schemes

Codes	Coded Text	Source
Design-construction defects		
Design-construction defects	tests [in 1988] revealed that the original design would not achieve guaranteed efficiency values	Lang et al. (2000)
Design-construction defects	EMGESA ha hecho cambios sustanciales a su propuesta de manejo del embalse El Quimbo, después de recibir la licencia, sin ningún pronunciamiento de las autoridades pertinentes	Dussán Calderón (2016)
Design-construction defects	Many people who spoke to Human Rights Watch, including flood victims, dam project workers and an engineer familiar with the construction of Tokwe-Mukorsi dam, contend that the floods could have been prevented through letting out water downstream of the dam through water regulation tunnels in the dam wall.	Human Rights Watch (2015)
Geological vulnerability of the site	It has taken the LHDA years to build replacement houses for displaced people. Many of those displaced by powerline construction in 1990-91, for example, were still without housing in October 1995, according to the World Bank. Others who lost homes to earthquakes caused when the Katse reservoirs were filled were forced to live in temporary storage-shed type housing for months, including over a very harsh winter. Houses in the Mohale resettlement sites already suffer from cracked walls.	Lang et al. (2000)
Geological vulnerability of the site	The reservoir had to be emptied immediately to avoid a disaster. Engineers then discovered around 70 sinkholes in the reservoir bed, which they tried covering<br>between 1975 and 1978 by dumping thousands of tonnes of earth by the barge load. One sinkhole that appeared in 1984 was still there in 1991 and could affect the permeability of the dam.42 Downstream of the dam, a huge 50 metre-deep, 300 metre-wide plunge pool formed in 1976 when the main spillway began operating. During the following year's floods, the rocks at the base of the spillway eroded, threatening the safety of the huge concrete spillway. The auxiliary spillway had similar problems and the contractors had to undertake a three-year rock stabilisation programme	Lang et al. (2000)
Geological vulnerability of the site	The waters of the Yellow river are the most silt-laden in the world, and other dams on the Yellow river have run into serious sedimentation problems.	Lang et al. (2000)
Geological vulnerability of the site	As regards to the basin, sedimentation is higher than expected and the reservoir is likely to be filled with debris in the coming future.	Colajacomo (1999)

Geological vulnerability of the site	As regards to the basin, sedimentation is higher than expected and the reservoir is likely to be filled with debris in the coming future. These factors contribute to shortening the dam's life: according to some sources it will not last for more than 20 years. In some areas north of the basin, sedimentation has reached 100% of live storage and is fast reaching the area at the entrance of the dam tunnel. This critical area of the dam, which represents the depth of the basin in front of the tunnel (which takes in the water to produce electricity), was at about 100 m. depth when the dam started operation, but was only at 70 m. depth in 1996.	Colajacomo (1999)
Geological vulnerability of the site	diversos testigos han evidenciado que el nivel de las aguas parece haber subido por lo menos una decena de metros por encima del previsto, alcanzando partes de las colinas que según las previsiones de la CEDEGE no habrían debido ser inundadas.	Gerebizza (2009)
Geological vulnerability of the site	la acumulación constante de materiales arenosos depositados por los ríos Daule y Peripa en el fondo del embalse, que en el curso de los años han disminuido su capacidad global de almacenamiento de agua	Gerebizza (2009)
Geological vulnerability of the site	la erosión afecta al 85% del embalse y ha llevado en el curso de los últimos años a una ulterior pérdida de tierra.	Gerebizza (2009)
Geological vulnerability of the site	zona inestable del punto de vista que los técnicos . En ese lugar hay diez fallas técnicas y cuando empezó obras hundimientos de 12 metros	Entrevista
Geological vulnerability of the site	existencia de una falla geológica en la zona de construcción de la represa	Galindo Vanegas (2018)
Geological vulnerability of the site	Risk of seismic activity in the Gibe III project region, with the possibility of a major seismically determined event – including earthquake and massive landslide potential.	ARWG (2009)
Geological vulnerability of the site	ARWG study predicts that 50-75% of the reservoir water could be lost due to underground cracks in geological rock formations.	International Rivers (2009)
Geological vulnerability of the site	The GOE discounts the seismic danger to the planned Gibe III dam, ignoring key geological information. The international development banks and bilateral agencies engaged with feasibility and impact studies as well as funding of the project—directly and indirectly—also ignore available data pointing to major seismic risk	Carr (2017)

Geological vulnerability of the site	Acotación: El geólogo Jaime Suarez Díaz, fundador de la Sociedad Santandereana de Geotecnia, manifestó que la represa de Hidrosogamoso tuvo que ver con el temblor que sacudió a Bucaramanga el 10 de marzo.	CENSAT-AguaViva (n.d.)
Geological vulnerability of the site	The filling of what was then the world's largest reservoir was followed by considerable earthquake activity. The weight of Kariba’s reservoir translates into a mass of 180 billion metric tons. The reservoir is located in a tectonically active area, at the southern end of the African Rift Valley. Since its construction and filling in the early 1960s, Kariba has caused numerous earthquakes in the area, 20 of them larger than magnitude 5 on the Richter scale. Project documents did not discuss the possibility of reservoir-induced seismicity and the need to take this into account in the design of the dam, so the seismic activity’s affect on the dam’s safety is unknown.	International Rivers (2009a)
Geological vulnerability of the site	The Kariba Dam is in a dangerous state. Opened in 1959, it was built on a seemingly solid bed of basalt. But, in the past 50 years, the torrents from the spillway have eroded that bedrock, carving a vast crater that has undercut the dam’s foundations. Engineers are now warning that without urgent repairs, the whole dam will collapse. If that happened, a tsunami-like wall of water would rip through the Zambezi valley, reaching the Mozambique border within eight hours. The torrent would overwhelm Mozambique’s Cahora Bassa Dam and knock out 40% of southern Africa’s hydroelectric capacity. Along with the devastation of wildlife in the valley, the Zambezi River Authority (ZRA) estimates that the lives of 3.5 million people are at risk.	Darbourn (2015)
Geological vulnerability of the site	The Kariba Dam is in a dangerous state. Opened in 1959, it was built on a seemingly solid bed of basalt. But, in the past 50 years, the torrents from the spillway have eroded that bedrock, carving a vast crater that has undercut the dam’s foundations.	Darbourn (2015)
Geological vulnerability of the site	If the dam fails, the water will arrive in Mosul in four hours. It will arrive in Baghdad in 45 hours. Some people say there could be half a million people killed, some say a million.	Borger (2016)
Geological vulnerability of the site	Iraqi engineers involved in building the Mosul dam 30 years ago have warned that the risk of its imminent collapse and the consequent death toll could be even worse than reported. They pointed out that pressure on the dam’s compromised structure was building up rapidly as winter snows melted and more water flowed into the reservoir, bri	Borger (2016)
Geological vulnerability of the site	Mosul dam engineers warn it could fail at any time, killing 1m people	Borger (2016)

Geological vulnerability of the site	n the preceding years, successive foreign consultants had pointed out the weaknesses in the rock formations but all assured the Iraqi government the problem could be solved by grouting.	Borger (2016)
Geological vulnerability of the site	Dams built on this kind of rock are subject to a phenomenon called karstification, in which the foundation becomes shot through with voids and vacuums. According to former Iraqi officials who worked on the project, successive teams of geologists reached the same conclusion: no matter where they looked, the prevalence of gypsum would make maintaining a dam difficult.	Filkins (2016)
Geological vulnerability of the site	The problem, according to Azzam Alwash, an Iraqi-American civil engineer who has served as an adviser on the dam, is that “it’s just in the wrong place.” Completed in 1984, the dam sits on a foundation of soluble rock.	Filkins (2016)
Geological vulnerability of the site	An in-depth study by the European Commission's Science Centre, released last April, puts the number of Iraqis that could be affected by the dam's floodwater after its collapse at seven million. The 58-page report by the Joint Research Centre simulates different scenarios that may result from the dam's breakdown.	Bibbo (2016)
Geological vulnerability of the site	Due to the dam's construction on a soluble bedrock, constant grouting is necessary to keep the structure from collapsing in upon itself.	Bender (2014)
Geological vulnerability of the site	Built on water permeable rock, the inherent instability of the Mosul dam has been known since it was built and this was mitigated by continuous grouting	Annunziato et al (2016)
Geological vulnerability of the site	Concerns about the dam’s stability and safety began already during construction: because the dam is built on a foundation of gypsum, anhydrite and limestone, i.e. water-soluble minerals, water can seep under the dam and compromise its stability, posing the risk of catastrophic failure (Al-Ansari et al 2015 and references therein – Ref. 1). To avoid this, continuous maintenance has taken place since the dam’s opening, in the form of grouting: new leaks are plugged by injecting a cement mixture into the compromised spots. This ad-hoc solution in itself is not deemed satisfactory, since grouting at one location causes the seepage of subsurface water to move to another location but does not stop it	Annunziato et al (2016)
Geological vulnerability of the site	Tunneling for hydropower project using the drill and blast technique in a fragile region like the Himalayas seems to be definitely having geological and hydrological impacts both of which need to be assessed urgently. Kinnaur is the second most landslide prone district in the state of Himachal. This is also a highly seismic zone.	Himdara (2015)
High costs of maintenance	Remedial work increased the cost of the dam from a projected $800 million to $1.5 billion.	Lang et al. (2000)

High costs of maintenance	Guatemala still spends US$ 150 million a year to produce electricity. Every year a minimum of US$ 8 million are spent on structural maintenance costs of the Chixoy project, and only when fully operating does it cover about 50-60% of the country's needs.	Colajacomo (1999)
High costs of maintenance	Maintenance costs are higher than planned and additional technical problems have required additional maintenance work.	Colajacomo (1999)
High costs of maintenance	A total of 95,000 tonnes of grout of different types were used over the dam's lifetime.	Borger (2016)
High costs of maintenance	Nasrat Adamo, the dam's former chief engineer who spent most of his professional career shoring it up in the face of fundamental flaws in its construction, said that the structure would only survive with round-the-clock work with teams filling in holes in the porous bedrock under the structure, a process known as grouting.	Borger (2016)
High costs of maintenance	On Wednesday, the Iraqi government announced it had signed a €273m (£210m) contract with an Italian contractor to reinforce and maintain the Mosul dam for 18 months,	Borger (2016)
High costs of maintenance	To keep it stable, hundreds of employees have to work around the clock, pumping a cement mixture into the earth below. Without continuous maintenance, the rock beneath would wash away, causing the dam to sink and then break apart.	Filkins (2016)
High costs of maintenance	Under a $300m contract, funded by the World Bank, the Italian company is doing maintenance and repair works, in addition to consolidating the foundations of the dam with injections of a cement mix, in a process called grouting.	Bibbo (2016)
High costs of maintenance	Due to the dam's construction on a soluble bedrock, constant grouting is necessary to keep the structure from collapsing in upon itself.	Bender (2014)
Oversizing or Undersizing	he project was economically justified on the assumption that Argentinian electricity demand would increase by 8-10% per year during the 1980s. In fact, demand grew by around just 2%, so that when the first turbines came on-line in the mid-1990s, Argentina already had a surplus of generating capacity.	Lang et al. (2000)
Oversizing or Undersizing	However, the level of water in the reservoir fell after it was filled in 1991, and on completion the dam was able to generate only 80152 MW, around half its installed capacity.	Lang et al. (2000)
Oversizing or Undersizing	In October 1999, the project was running at half capacity and was expected to lose more than Rmb1bn ($72.2 million) over the year.	Lang et al. (2000)
Oversizing or Undersizing	the dam is operating at only 60% of its installed capacity, below the project's financial break-even point.	Lang et al. (2000)
Oversizing or Undersizing	The dam started to generate electricity in 1998, but by September that year, the Xinhua News Agency reported that Liu Junfeng, the general manager of Ertan Hydropower Development	Lang et al. (2000)

	Corporation, • has become increasingly anxious• because he could sell only 60% of the dam's output.	
Oversizing or Undersizing	The project was economically justified on the assumption that Argentinian electricity demand would increase by 8-10% per year during the 1980s. In fact, demand grew by around just 2%, so that when the first turbines came on-line in the mid-1990s, Argentina already had a surplus of generating capacity.	Lang et al. (2000)
Oversizing or Undersizing	under-performance (20 hour per week black-outs during the 1994 drought)	Lang et al. (2000)
Oversizing or Undersizing	different from the purported dam capacity (250 MW) to date, the dam has not generated to its expected 250 MW. Recent records so far seen indicate that the dam is producing only between 150–155 MW as opposed to 250 MW.	NAPE (2014)
Oversizing or Undersizing	the plant has never operated at more than 70% of its expected capacity.	Colajacomo (1999)
Oversizing or Undersizing	I think the dam is sized for the peak flow rate of the river, which lasts just a few months. The peak flow rate of Blue Nile is under 6,000 cubic meters per second (mcs), even exceeding 6,500 mcs once in a while. With 145 meters of dam height, this peak flow can produce about 7,000 MW. The average flow rate of Blue Nile is reported to be much lower. So, given the height of the dam and the flow rate, there is no way the dam can produce 6,000 MW for more than 3 months of the year even if the dam stored the difference between peak-flow and design-flow rates. The only scenario under which the power output will be annually consistent is if the hydroelectric dam is designed for a mean flow, which is about 1,456 mcs. This will provide just less than 2,100 MW.	International Rivers (2013)
Oversizing or Undersizing	Engineers' Concerns […] A number of experts believe the dam is not going to produce as much power as is claimed, and that the dam should be smaller in size for efficiency and cost. Asfaw Beyene, a Professor of Mechanical Engineering at San Diego State University (California) says the dam is 300% over-sized (/node/8082) . "More than half of the turbines will be rarely used," he says. "GERD's available power output, based on the average of river flow throughout the year and the dam height, is about 2,000 megawatts, not 6,000. There is little doubt that the system has been designed for a peak flow rate that only happens during the 2-3 months of the rainy season. Targeting near peak or peak flow rate makes no economic sense." Beyene notes that that the issue is so highly politicized that "it seems to suppress legitimate engineering inputs and environmental discussions." He suggests that the concerned authorities should make the project transparent, and resize the hydroelectric power output by reducing the number of turbines.	International Rivers (2014)

Oversizing or Undersizing	A mediados de 1994 la represa era capaz de generar a lo sumo la mitad de su capacidad y los usuarios de electricidad hondureños sufrieron cortes de hasta 14 horas diarias.	McCully (2001)
Poor or none feasibility and alternatives' study	LHWP's proponents did not study Lesotho's potential alternative energy sources, nor did they not study water conservation as an option	Lang et al. (2000)
Poor or none feasibility and alternatives' study	Presentaba derechos de agua insuficientes para su funcionamiento y en litigio legal en los tribunales de justica además de una violación al acuerdo de Solución Amistosa de Ralco; el cual establecía el compromiso del Estado de Chile que comprometía lo siguiente: "acordar mecanismos vinculantes para todos los órganos del Estado que aseguren la no instalación de futuros mega proyectos, particularmente hidroeléctricos, en tierras indígenas del alto Bío Bío"	OLCA (2014)
Poor or none feasibility and alternatives' study	An analysis of the risks of climate change on Uganda's energy sector and its economy has not been undertaken. Uganda has vast renewable energy resources in the form of solar, geothermal and wind. These are environmentally friendly sources of energy that need to be exploited and promoted. NAPE published a study in 2005 showing that Uganda's geothermal potential stand at 450MW but could be more<br>These pro-poor energy options like solar, wind, biomass and geothermal have not been adequately studied to provide evidence that Bujagali dam project is the least-cost option. Although there have been various efforts in the recent past to analyze Uganda's renewable energy potential, the bloated dam project had the effect of stifling development of viable renewable energy options.	NAPE (2014)
Poor or none feasibility and alternatives' study	Il progetto non dispone di una dettagliata analisi sui costi-benefici di lungo termine.	Manes (2012)
Poor or none feasibility and alternatives' study	In relazione al punto 8, lo studio indica in 110.480.746 dollari il costo complessivo del progetto. Lo studio non presenta dettagli sul costo delle misure descritte nel piano di gestione ambientale, per tanto non esistono garanzie che esso venga implementato.	Manes (2012)
Poor or none feasibility and alternatives' study	Lo studio non presenta un'analisi dettagliata delle alternative.	Manes (2012)
Poor or none feasibility and alternatives' study	Lo studio omette una valutazione dettagliata degli impatti sinergici e cumulativi di lungo termine. Il piano di gestione ambientale non offre alcuna garanzia in merito alla sua implementazione. Fra le altre cose, manca il budget, la garanzia di finanziamento, i meccanismi di decisione e le responsabilità e il personale incaricato della sua esecuzione.	Manes (2012)

Poor or none feasibility and alternatives' study	La Asociación de Afectados por el Proyecto Hidroeléctrica El Quimbo -Asoquimbo- ha demostrado que el problema se originó en el desconocimiento por parte del Gobierno del presidente Alvaro Uribe del Auto No. 517 de 31 de julio de 1997 mediante el cual El Ministerio de Ambiente declaró no viable el Proyecto Hidroeléctrico El Quimbo, sin embargo, en el 2007 subastó el Proyecto a Emgesa y declaró mediante Auto No 515 del 22 de febrero de 2008 que El Quimbo "No requería de la presentación de Diagnóstico Ambiental de Alternativas (DAA) y otorgó Licencia Ambiental Mediante Resolución 0899 del 15 de mayo de 2009.<br>Seis años después de expedida la Licencia los hechos demuestran que los daños económicos, sociales, ambientales y culturales son irreparables como se advirtió desde un comienzo cuando se sustentó que el proyecto era inviable fundamentalmente por dos razones: La primera, porque no existía justificación técnica para "la desproporción entre el área de inundación y la poca generación de electricidad". El proyecto El Quimbo necesita 20 hectáreas de embalse para instalar una unidad de potencia de energía (Gigawatio), mientras una hidroeléctrica en cualquier otro lugar de Colombia apenas necesitaría dos hectáreas. La segunda, por la afectación estructural del área productiva, no es conveniente que se inunden las mejores tierras con vocación agrícola de la región y sería imposible restituir la actividad productiva en la zona.	Dussán Calderón (2016)
Poor or none feasibility and alternatives' study	The GOE discounts the seismic danger to the planned Gibe III dam, ignoring key geological information. The international development banks and bilateral agencies engaged with feasibility and impact studies as well as funding of the project—directly and indirectly—also ignore available data pointing to major seismic risk.	Carr (2017)
Poor or none feasibility and alternatives' study	No such environmental flow requirement was determined prior to construction. Since then project authorities have resisted efforts to increase downstream flows, because they claim additional releases will not allow them to produce enough power. The project feasibility study only analyzed impacts on habitat flooded by the reservoir. The report failed to mention any impacts on the Kihansi Gorge ecosystem.	International Rivers (2001)
Poor or none feasibility and alternatives' study	LHWP feasibility studies completed during the 1983-1985 period not only underestimated the number of potential resettlers and other project affected people but also seriously underestimated environmental impacts with the project authorities subsequently concluding that "there were no major environmental obstacles to the implementation of the project"	Lenka Thamae and Pottinger (2006)
Poor or none feasibility and alternatives' study	Also the recently published cost-benefit analysis (https://bankwatch.org/wpcontent/uploads/2017/12/Nenskra-costbenefitanalysis-Jul2017.pdf) contains gaps like a comparison with investments in energy efficiency as an	Chipashvili (2017)

	alternative to the Nenskra dam. More importantly, it doesn't really include an analysis of any costs.	
Poor or none feasibility and alternatives' study	Many people who spoke to Human Rights Watch, including flood victims, dam project workers and an engineer familiar with the construction of Tokwe-Mukorsi dam, contend that the floods could have been prevented through letting out water downstream of the dam through water regulation tunnels in the dam wall.	Human Rights Watch (2015)
Structural and components' defects	A week later one of the two remaining active tunnels collapsed, bringing down nearly half a million cubic metres of concrete and rock.	Lang et al. (2000)
Structural and components' defects	arbela almost led to the collapse of the dam during the initial filling of its reservoir in the 1970s.	Lang et al. (2000)
Structural and components' defects	The dam has been plagued with problems ever since 1974 when the reservoir impoundment began. Two of the four tunnels being used to control the rate of filling were damaged and forced to close. A week later one of the two remaining active tunnels collapsed, bringing down nearly half a million cubic metres of concrete and rock	Lang et al. (2000)
Structural and components' defects	The turbines of Ghana's Akosombo dam have long failed to work properly	Lang et al. (2000)
Structural and components' defects	Three turbines had to be taken out of service in 1998 at a cost of $5 million in lost production when cracks appeared	Lang et al. (2000)
Structural and components' defects	The Nalubaale dam powerhouse and the bridge have cracks and pose a risk to the Bujgali dam downstream	NAPE (2014)
Structural and components' defects	The power plant started operating after the reservoir was filled in 1983. However, it was shut down five months later because it was feared that the tunnel carrying water from the reservoir to the plant was about to collapse.	Colajacomo (1999)
Structural and components' defects	falta de planificación del proyecto y en consecuencia por los efectos no esperados de la construcción de la hidroeléctrica	Galindo Vanegas (2018)
Structural and components' defects	Kariba, like many dams, has been affected by a condition that mars its concrete, known as "alkali-aggregate reaction." While regional concern about the dam's condition is widespread, little is known about the extent of the problem or its implications for dam safety	International Rivers (2009a)
Structural and components' defects	The dam was constructed on highly karstified beds of the Fatha Formation. In view of this fact, grouting operations were conducted during the construction period to fill the cavities, fissures, joints and cracks in the karstified beds. Unfortunately, all the executed efforts did not stop the seepage under the foundation of the dam. After impounding in 1986, new seepage locations were recognized. Grouting operations continued and various studies were conducted to find suitable grout or technique to overcome this problem. The seepage due to the dissolution of	Al-Ansari et al (2015)

	gypsum and anhydrite beds raised big concern about the safety of the dam and its possible failure.	
Structural and components' defects	Concerns about the dam's stability and safety began already during construction: because the dam is built on a foundation of gypsum, anhydrite and limestone, i.e. water-soluble minerals, water can seep under the dam and compromise its stability, posing the risk of catastrophic failure (Al-Ansari et al 2015 and references therein – Ref. 1). To avoid this, continuous maintenance has taken place since the dam's opening, in the form of grouting: new leaks are plugged by injecting a cement mixture into the compromised spots. This ad-hoc solution in itself is not deemed satisfactory, since grouting at one location causes the seepage of subsurface water to move to another location but does not stop it.	Annunziato et al (2016)
Structural and components' defects	The value for the maximum wave height in Mosul in these calculations is in good agreement with the values estimated in the study of Swiss Consultants in 1984 (24m).	Annunziato et al (2016)
Structural and components' defects	The Financial Gazette can reveal that while a recent risk assessment ruled out the possibility of the dam wall collapsing, the conundrum dramatically playing out at TokweMukosi has everything to do with a 1992 decision to change the dam's design in order to cut costs.	Chenga (2014)
Structural and components' defects	water is now seeping through the dam wall, heightening concerns that if the seepage persists it could further weaken the massive structure.	Chenga (2014)
Financial unsustainability		
Financial un-sustainability	Financing has not been found for the $857 million worth of additional construction work required to fill the reservoir, and for past and future resettlement and environmental mitigation costs.	Lang et al. (2000)
Financial un-sustainability	Guatemala still spends US$ 150 million a year to produce electricity. Every year a minimum of US$ 8 million are spent on structural maintenance costs of the Chixoy project, and only when fully operating does it cover about 50-60% of the country's needs.	Colajacomo (1999)
Financial un-sustainability	Moreover, the dam works in a very discontinuous way: in summer the energy demand is not sufficient to cover the total supply of the country and so the Chixoy power is not used.	Colajacomo (1999)
Financial un-sustainability	The dam has turned out to be a financial disaster, since it does not cover the country's energy needs.	Colajacomo (1999)
Financial un-sustainability	Il progetto non dispone di una dettagliata analisi sui costi-benefici di lungo termine.	Manes (2012)
Financial un-sustainability	In relazione al punto 8, lo studio indica in 110.480.746 dollari il costo complessivo del progetto. Lo studio non presenta dettagli sul costo delle misure descritte nel piano di gestione ambientale, per tanto non esistono garanzie che esso venga implementato.	Manes (2012)

Financial un-sustainability	Lo studio omette una valutazione dettagliata degli impatti sinergici e cumulativi di lungo termine. Il piano di gestione ambientale non offre alcuna garanzia in merito alla sua implementazione. Fra le altre cose, manca il budget, la garanzia di finanziamento, i meccanismi di decisione e le responsabilità e il personale incaricato della sua esecuzione.	Manes (2012)
Financial un-sustainability	The government says it will finance the costly project itself, and has developed a plan to sell dam bonds directly to citizens at home and abroad, and to private companies. Various reports say bond sales are not meeting expectations, due to "risk perceptions"" among investors. Meetings to sell the bonds have met with protests in a number of cities around the world (for example San Diego (http://sandiegofreepress.org/2013/04/protest-in-city-heights-of-controversial-ethiopian-consulate-meeting/) and Canada (http://www.youtube.com/watch?v=DtikdU8FdVw%20Switzerland%20http://ethsat.com/2013/06/05/ethiopians-in-switzerland-abort-a-pro-govt-meeting/)).<br>Pressure to buy the bonds is intense. The Brookings Institute reports: (http://www.brookings.edu/blogs/up-front/posts/2013/07/23-ethiopia-hydroelectric-powerplant-mbaku) "Government employees have been encouraged to devote as much as one or two months of their salaries to the purchasing of the GERD bonds. Most public workers in Ethiopia earn relatively low wages and face a significantly high cost of living. Hence, they are not likely to be able to sacrifice that much of their salaries to invest in this national project. Nevertheless, many of them have been observed purchasing the GERD bonds, primarily because of pressure from the government and the belief that participation in this national project is a show of one's patriotism."	International Rivers (2014)
Financial un-sustainability	Tajik government has raised funds through a public offering of shares. According to a 2010 UN World Food Programme report, as Tajik citizens were requested to buy Rogan shares, sometimes under threat of losing their jobs	Skoba (2013)
Electricity overproduction	The dam started to generate electricity in 1998, but by September that year, the Xinhua News Agency reported that Liu Junfeng, the general manager of Ertan Hydropower Development Corporation, • has become increasingly anxious• because he could sell only 60% of the dam's output. Sichuan province has an electricity glut after a steady slowdown in economic growth from 12.6% in 1994 to just 7% in 1999.16	Lang et al. (2000)
Electricity overproduction	The project was economically justified on the assumption that Argentinian electricity demand would increase by 8-10% per year during the 1980s. In fact, demand grew by around just 2%, so that when the first turbines came on-line in the mid-1990s, Argentina already had a surplus of generating capacity	Lang et al. (2000)

Electricity overproduction	Moreover, the dam works in a very discontinuous way: in summer the energy demand is not sufficient to cover the total supply of the country and so the Chixoy power is not used.	Colajacomo (1999)
Increase of energy tariffs and public debt	A second, but potentially more serious problem, is that the electricity generated at Ertan is significantly more expensive than that produced by smaller power stations that have sprung up since Ertan's inception in 1991	Lang et al. (2000)
Increase of energy tariffs and public debt	ANDE wants to increase electricity tariffs by 30% to overcome its critical financial state	Lang et al. (2000)
Increase of energy tariffs and public debt	The dam started to generate electricity in 1998, but by September that year, the Xinhua News Agency reported that Liu Junfeng, the general manager of Ertan Hydropower Development Corporation, • has become increasingly anxious• because he could sell only 60% of the dam's output. Sichuan province has an electricity glut after a steady slowdown in economic growth from 12.6% in 1994 to just 7% in 1999.	Lang et al. (2000)
Increase of energy tariffs and public debt	The project was economically justified on the assumption that Argentinian electricity demand would increase by 8-10% per year during the 1980s. In fact, demand grew by around just 2%, so that when the first turbines came on-line in the mid-1990s, Argentina already had a surplus of generating capacity.	Lang et al. (2000)
Increase of energy tariffs and public debt	Aquí en la región se genera el 30% de la energía de Chile, sin embargo, los costos de esta son los más altos del país. Aquí en Quilaco, Santa Bárbara y Alto Bio Bio se paga una tarifa domiciliaria por concepto de energía mucho más alta que en Santiago, Concepción o Calama, sin embargo, el costo social, ambiental, cultural, emocional, etc. nosotros lo hemos pagado, y de acuerdo al mensaje entregado por la propia presidenta Michelle Bachelet en la inauguración de la Central Angostura -en donde avala este modelo- seguiremos pangándolo mientras la normativa continúe en su rol actual.	OLCA (2014)
Increase of energy tariffs and public debt	Aquí en la región se genera el 30% de la energía de Chile, sin embargo, los costos de esta son los más altos del país. Aquí en Quilaco, Santa Bárbara y Alto Bio Bio se paga una tarifa domiciliaria por concepto de energía mucho más alta que en Santiago, Concepción o Calama, sin embargo, el costo social, ambiental, cultural, emocional, etc. nosotros lo hemos pagado, y de acuerdo al mensaje entregado por la propia presidenta Michelle Bachelet en la inauguración de la Central Angostura -en donde avala este modelo- seguiremos pangándolo mientras la normativa continúe en su rol actual.	OLCA (2014)
Increase of energy tariffs and public debt	Unfortunately, the expected reduction in electricity costs has not been realized, and instead the average cost of electricity increased after the dam was commissioned. It's now the most expensive hydropower in Africa, making it unaffordable for many Ugandans	International Rivers (n.d)

Increase of energy tariffs and public debt	Unfortunately, the expected reduction in electricity costs has not been realized, and instead the average cost of electricity increased after the dam was commissioned. It's now the most expensive hydropower in Africa, making it unaffordable for many Ugandans	International Rivers (n.d)
Increase of energy tariffs and public debt	Equally the current tariff of UGX 520.60 (USD Cents 2.01) per unit for domestic consumers is high for an ordinary Ugandan and is a threefold increase since before the onset of the dam construction. This is opposed to a reduction of power cost - a lie that was traded for social acceptance and Banks approval of the dam project. Uganda's electricity tariff is the highest in the East African region.	NAPE (2014)
Increase of energy tariffs and public debt	Equally the current tariff of UGX 520.60 (USD Cents 2.01) per unit for domestic consumers is high for an ordinary Ugandan and is a threefold increase since before the onset of the dam construction. This is opposed to a reduction of power cost - a lie that was traded for social acceptance and Banks approval of the dam project. Uganda's electricity tariff is the highest in the East African region.	NAPE (2014)
Increase of energy tariffs and public debt	Because of the dam, the country's national debt has increased substantially, with the population paying the real costs of the whole operation. INDE incurred a US$40 million debt. As of 1991, INDE's debt accounted for 45% of Guatemala's foreign debt. Meanwhile electricity prices have increased, in part to pay for the debt of this project.	Colajacomo (1999)
Increase of energy tariffs and public debt	Energy costs supported by the population have constantly increased during the last few years, but still only 30% of the population benefit from electric power.	Colajacomo (1999)
Increase of energy tariffs and public debt	Energy costs supported by the population have constantly increased during the last few years, but still only 30% of the population benefit from electric power.	Colajacomo (1999)
Increase of energy tariffs and public debt	Meanwhile electricity prices have increased, in part to pay for the debt of this project.	Colajacomo (1999)
Increase of energy tariffs and public debt	Until 1974, Guatemala had only $120m (£75m) of debt. Thereafter, debt increased rapidly, by $100m a year or more in 1978, 1979 and 1980, and then more than $250m a year in 1981 and 1982, at the height of the terror. By 1985 the country's debt had reached $2.2bn – an increase of more than $2bn in 10 years.	Dearden (2012)
Increase of energy tariffs and public debt	En 1995 era además evidente que numerosos préstamos ya contraídos por los diversos gobiernos ecuatorianos para la construcción del mega proyecto infraestructural no estaban alcanzando los resultados previstos.<br>En aquellos años, Ecuador estaba, además, atravesando una crisis cada vez mayor de la deuda, generada en gran parte por los numerosos préstamos contraídos para la construcción de la represa y de las otras obras infraestructurales incluidas en el proyecto Jaime Roldos Aguilera, con importantes consecuencias económicas y sociales, que los ulteriores créditos necesarios	Gerebizza (2009)

	para la construcción de […] De acuerdo al informe de la CAIC... ... en la fase de planeación los beneficios del proyecto habían sido tal vez exagerados. Por ejemplo, en los contratos de crédito se estableció el objetivo de “poner en producción bajo riego de 100.000 hectáreas, de las cuales 50.000 en ambas márgenes del rio Daule”. El Informe subraya en cambio que “en la realidad solo se ejecutaron 17.000 hectáreas” y “tampoco se ocurrieron los beneficios de navegación, recreación y turismo, que fueron previstos. De hecho, occurrió todo lo contrario”. En 1994, Italia era el primer acreedor bilateral de Ecuador, con créditos totales correspondientes al 22% de la deuda contraída por Ecuador con el Club de Paris.	
Increase of energy tariffs and public debt	ngentes montos de recursos, casi en su totalidad provenientes del endeudamiento externo”. Para su construcción entre 1982 y 2006, Ecuador contrajo préstamos por un total de 1.203.302.432 dólares, de los cuales más de 248 millones se destinaron sólo a la central hidroeléctrica de Daule Peripa/Marcel Laniado De Wind. Una cifra astronómica para un proyecto que no ha generado los beneficios esperados y para una central que hoy produce en promedio sólo un tercio de la potencia prevista.	Gerebizza (2009)
Increase of energy tariffs and public debt	La obra es la primera que se vende bajo el esquema de subasta por cargo de confiabilidad, que significa que a Emgesa se le garantiza “un ingreso fijo independiente de su participación diaria en el mercado mayorista asignado por un período de 20 años, reduciendo así el riesgo de su inversión”, pago que se suma a la factura de los usuarios	Dussán Calderón (2016)
Increase of energy tariffs and public debt	La obra es la primera que se vende bajo el esquema de subasta por cargo de confiabilidad, que significa que a Emgesa se le garantiza “un ingreso fijo independiente de su participación diaria en el mercado mayorista asignado por un período de 20 años, reduciendo así el riesgo de su inversión”, pago que se suma a la factura de los usuarios	Dussán Calderón (2016)
Increase of energy tariffs and public debt	Preliminary financial estimates done by International Rivers show that the project has a Net Present Value (#_ftn1) [1] of -$640 million, with an estimated project cost of $4.8 billion over a fifty-year period, which may lead to increased national debt (Sanyanga et al, 2016).	International Rivers (2017)
Increase of energy tariffs and public debt	The government says it will finance the costly project itself, and has developed a plan to sell dam bonds directly to citizens at home and abroad, and to private companies. Various reports say bond sales are not meeting expectations, due to “risk perceptions”” among investors. Meetings to sell the bonds have met with protests in a number of cities around the world (for example San Diego (http://sandiegofreepress.org/2013/04/protest-in-city-heights-of-controversial-ethiopian-consulate-meeting/) and Canada (http://www.youtube.com/watch?v=DtikdU8FdVw%20Switzerland%20http://ethsat.com/2013/06/05/ethiopians-in-switzerland-abort-a-pro-govt-meeting/)).	International Rivers (2014)

	Pressure to buy the bonds is intense. The Brookings Institute reports: (http://www.brookings.edu/blogs/up-front/posts/2013/07/23-ethiopia-hydroelectric-powerplant-mbaku) “Government employees have been encouraged to devote as much as one or two months of their salaries to the purchasing of the GERD bonds. Most public workers in Ethiopia earn relatively low wages and face a significantly high cost of living. Hence, they are not likely to be able to sacrifice that much of their salaries to invest in this national project. Nevertheless, many of them have been observed purchasing the GERD bonds, primarily because of pressure from the government and the belief that participation in this national project is a show of one’s patriotism.”	
Increase of energy tariffs and public debt	En la comuna de Alto Biobío existe un alto porcentaje de población Pehuenche y es una de las comunas más pobres del país. El 65 por ciento de su población están por debajo de la línea de la pobreza y pese a estar al lado de las centrales hidroeléctricas de Ralco y Pangue, sus habitantes pagan $ 150 el kilowatt (kW), casi el doble que en Concepción y un 67 por ciento más cara que en Santiago.	ElDesconcierto (2014)
Increase of energy tariffs and public debt	Sin embargo, a pesar de que el proyecto favoreció la economía local durante su puesta en marcha, el precio de la energía en la zona continúa siendo uno de los más caros del país	ElDesconcierto (2014)
Increase of energy tariffs and public debt	Molteplici aziende hanno iniziato da allora ad arricchirsi con i lavori di iniezione cementifera nelle falle che si aprono in continuazione sotto la struttura, pagati ora dai contribuenti statunitensi, ora da quelli iracheni, ora attraverso un ulteriore indebitamento dell'Iraq con la Banca Mondiale (200mln di dollari per questi interventi	InfoAut (2016)
Increase of energy tariffs and public debt	Yet it did reveal a guaranteed price of USD 0.08532 per kWh at which the government of Georgia will have to buy electricity from Nenskra – almost twice the current wholesale price (USD 0.047) for electricity bought in Georgia and imported from abroad.	Chipashvili (2017)
Increase of energy tariffs and public debt	Tajik government has raised funds through a public offering of shares. According to a 2010 UN World Food Programme report, as Tajik citizens were requested to buy Rogan shares, sometimes under threat of losing their jobs	Skoba (2013)
Increase of energy tariffs and public debt	Vecinos de comunas como Alto Bío Bío, Santa Bárbara y Quilaco, además de integrantes de la asamblea ciudadana que reúne a esas tres comunas, protestaron por la instalación de centrales y el altísimo costo que pagan por la electricidad pese a existir 4 generadoras en el cause del rió Bío Bío.	Osses (2014)
Increase of energy tariffs and public debt	El proyecto hidroeléctrico Yacyretá ha causado una pérdida neta a las economías de Argentina y Paraguay. El valor presente neto de la inversión es mas de 8,5 mi millones negativos, considerando los costos y beneficios desde su inicio y proyectando los futuros costos y beneficios.	Terol and Reid (2004)

Increase of energy tariffs and public debt	la magnitud del pasivo es tal que el inclusive aumentar en 70% la producción de Yacyretá no podrá saldarlo.	Terol and Reid (2004)
Project Cost overrun	In February 1997, the World Bank authorised its Inspection Panel to • review and assess• the problems with the dam, following a request by the Paraguayan environmental human rights NGO, Sobrevivencia - Amigos de la Tierra Paraguay, and a group of people affected by Yacyreta. The Panel estimated that at least another $2 billion would be needed to mitigate effectively the impacts of filling the reservoir to its final design level of 83 metres above sea level. The Panel stated, • one has to question seriously whether (Yacyreta's) debt would ever be repaid at 83 metres once the full costs are taken into account.	Lang et al. (2000)
Project Cost overrun	Remedial work increased the cost of the dam from a projected $800 million to $1.5 billion.	Lang et al. (2000)
Project Cost overrun	The Bank found that rectifying the mistakes would require at least another $130 million and take until the year 2000	Lang et al. (2000)
Project Cost overrun	The dam's costs soared from an original estimate of $2.7 billion to $11.5 billion	Lang et al. (2000)
Project Cost overrun	The rescue works on the Tarbela dam boosted the cost from an estimated $800 million in 1968 to $1.5 billion by 1986.	Lang et al. (2000)
Project Cost overrun	Three turbines had to be taken out of service in 1998 at a cost of $5 million in lost production when cracks appeared	Lang et al. (2000)
Project Cost overrun	Independent analysts see the Kali Gandaki controversy as a text-book case of why large foreign-funded and foreign-built projects turn out to be so expensive.	Khadka (2003)
Project Cost overrun	Under pressure to get the Kali Gandaki hydroelectric plant inaugurated by King Gyanendra by next month, Nepal Electricity Authority (NEA) is trying to hush up controversy over a shady transfer of cost overruns in Nepal's biggest power project. The NEA board initially wanted an explanation about the unauthorised payment of $30 million to the project's Italian civil contractor without its approval	Khadka (2003)
Project Cost overrun	By the time the Bujugali Dam was completed, the total costs had skyrocketed from the initial US$800 Million to $1.3 billion	International Rivers (n.d)
Project Cost overrun	The dam cost kept on growing from $580 million at inception to $860 million and fi nally $902 million ($3.6million per MW) at completion. Independent investigations by the Ugandan Parliamentary adhoc committee on energy put the dam's actual cost at $1.3 billion ($5.2million per MW or more).	NAPE (2014)
Project Cost overrun	The final cost of the project has not yet been clearly defined. Evaluations range from US$1.2 billion (521% higher than predicted) to US$2.5 billion	Colajacomo (1999)
Project Cost overrun	An Italian contracting company, Salini Impregilo JVC, began construction of Tokwe-Mukorsi dam in 1998 but stopped several times due to funding challenges. When construction resumed	Human Rights Watch (2015)

	in April 2011, it was estimated that the project would be completed within 31 months at a cost of US$133.8 million.4 2015 at the cost of $298.7 million.	
Project Cost overrun	Originally budgeted at $2.5 billion during the period of military dictatorships in Argentina and Paraguay, the project's total cost has now exceeded $15 billion	International Rivers (n.d./a)
Time overrun	the project was at least two years behind schedule	Lang et al. (2000)
Time overrun	The project, originally scheduled to be completed in 1989, is still not finished, and the most recent estimated completion date is 2001	Lang et al. (2000)
Time overrun	The Bujagali hydropower dam development was marred with controversies that saw the dam take over 18 years to complete.	NAPE (2014)
Time overrun	for a long time the Bumbuna dam long risked remaining only a collective fantasy, at most an unfinished colossus exposed to the erosion of time. The word 'Bumbuna' thus became for the people of Sierra Leone a synonym of 'a never-ending story' (cf. Mazzei and Scuppa 2006: 15). 'Bumbuna', ironically sang the well-known Sierra Leonean singer Emerson Bockarie in the years immediately following the end of the war – 'will be finished only at the end of the world' (Awareness Times 2008b)	D'Angelo (2014)
Time overrun	there was a widespread opinion among the inhabitants of the Tonkolili District that the works to complete the power station had been deliberately slowed down by the Italian constructors themselves (Mazzei and Scuppa 2006). In this way they would have had more time to secretly mine and smuggle out the gold and diamonds found during the construction of the dam.	D'Angelo (2014)
Time overrun	Bumbuna was becoming a joke. "When Bumbuna is completed" became a popular phrase, which indicated, "never."	MAZZEI AND SCUPPA (2006)
Time overrun	The construction is reportedly lagging behind schedule	Hussein (2014)
Time overrun	la construcción de la represa solo tiene 87,19% de avance y está paralizada.	Transparencia Venezuela (2018)
Time overrun	Transparencia Venezuela, 04 de diciembre de 2018. Once años han pasado desde que comenzó la construcción de la Central Hidroeléctrica Manuel Piar, mejor conocida como represa de Tocoma, en el sur del país y, pese a que debió estar lista en 2012, aún no ha aportado ningún megavatio al Sistema Eléctrico Nacional.	Transparencia Venezuela (2018)
Time overrun	proyecto Tocoma, el cual según las claúsulas Tercera y Cuarta del contrato ~rmado entre el Estado venezolano y la constructora brasileña Odebrecht el 19 de marzo de 2007, la obra debía de haber sido culminada en junio de 2013.	El Pitazo (2019)
Geopolitical and interstate conflict		

Geopolitical and interstate conflicts	In September, South African troops invaded Lesotho, ostensibly to restore order in the face of public protests against the government. In fact, the invasion was prompted in large part by a concern to protect the Lesotho Highlands project — South Africa's largest investment in the region. When the shooting was over, 17 people had been killed near the project's Katse dam and many more had died fighting in the capital, which was left in ruins. South Africa's Star newspaper stated: • Protection of the dam and its pipeline supplying [the region] with water was a top priority of the occupation forces.	Lang et al. (2000)
Geopolitical and interstate conflicts	The area around the dam was the centre of clashes between the various armed groups involved in the conflict. The village of Bumbuna was sacked and destroyed by the rebels and the dam became a strategic target.	D'Angelo (2014)
Geopolitical and interstate conflicts	Moreover, a tribal conflict between communities in the project area was stirring up opposition to the project.	MAZZEI AND SCUPPA (2006)
Geopolitical and interstate conflicts	The dam became a strategic target for rebel forces during the conflict and witnessed two major battles fought around its premises. These battles led to the near complete destruction of the village of Bumbuna and to extensive human losses in the nearby communities. The dam site itself was attacked several times but never seized by the rebel troops	MAZZEI AND SCUPPA (2006)
Geopolitical and interstate conflicts	In a further blow to Egypt, its alliance with Sudan faltered in 2012 when Sudan, which gets 35 percent of the Nile water according to the 1959 treaty, rescinded its initial opposition to Ethiopia's renaissance dam.	Hussein (2014)
Geopolitical and interstate conflicts	On Jan. 8, Ethiopia turned down (http://www.bloomberg.com/news/2014-01-08/ethiopiarejects-egyptian-proposal-on-nile-as-dam-talks-falter.html) Egypt's demand that it suspend construction of its mega-dam on the Nile, further escalating tensions between the two states. Fearing that Ethiopia's $4.2 billion project would reduce the river's flow, Egypt calls for a halt in construction until the dam's downstream impact is determined. Otherwise, it has vowed to protect its "historical rights" to the Nile at "any cost."	Hussein (2014)
Geopolitical and interstate conflicts	Egyptian politicians - including president Mohamed Morsi - have been caught live on air discussing plans to sabotage an Ethiopian dam project.	ABC News (2013)
Geopolitical and interstate conflicts	A threat of potentially existential proportions has emerged that could encroach on the single source of livelihood of over 100 million Egyptians"	Zane (2020)
Geopolitical and interstate conflicts	Egyptian Foreign Minister Sameh Shoukry has previously warned that filling and operating the dam without an agreement "that protects the downstream communities… would heighten tensions and could provoke crises and conflicts that further destabilise an already troubled region".	BBC News (2020)

	A conflict between Egypt and Ethiopia, which are both US allies, would put millions of civilians at risk.	
Geopolitical and interstate conflicts	Ethiopia has started filling the Grand Ethiopian Renaissance Dam, as Egypt still calls it an ‘existential threat’.	Roussi (2020)
Geopolitical and interstate conflicts	In an extension of a bilateral dispute between Ethiopia and Egypt over the $4.8 billion Grand Ethiopian Renaissance Dam being built on the Nile River, Egyptian hackers launched a cyber attack on a number of Ethiopian government websites over the course of the past week.	Zelalem (2020)
Geopolitical and interstate conflicts	At roughly the same time, a violent cross-border conflict on the border with Kenya ended with 540 deaths and the forced displacement of around 65,000 people in what appears to have been a revenge among the Turkana of Kenya against the Dassanech and Nyangatom of Ethiopia. In four attacks, which occurred on May 4, 2012, August 29, 2013, September 16, 2014 and October 8, 2015, due to inter-ethnic conflict that broke out in the name of limited resources, about 320 people died. From testimonies gathered among the inhabitants of the Dassanech woreda in several subsequent attacks, the Turkana took 2464 head of cattle. The theft of 77 donkeys and 18 cows from the Nyangatom community was also reported, and 300 fishing nets and nine calves from Dassanech, always by the Turkana.	Franchi and Manes (2016)
Geopolitical and interstate conflicts	The dwindling of resources caused by the dam would increase conflicts between local ethnic groups.	International Rivers (2009)
Geopolitical and interstate conflicts	Anche se cooperano ed effettuano scambi commerciali, tra alcuni di questi popoli si verificano periodicamente dei conflitti per l’utilizzo delle scarse risorse naturali. Con la progressiva sottrazione di terre da parte del governo, la competizione è andata crescendo e l’introduzione delle armi da fuoco ha reso i litigi più pericolosi di un tempo.	Survival International (2019)
Geopolitical and interstate conflicts	Italy has said it plans to send 450 troops to protect the dam site,	Borger (2016)
Geopolitical and interstate conflicts	propaganda video posted online shows a fighter carrying a flag across, and a man’s voice says, “The banner of unification ~utters above the dam.”	Filkins (2016)
Geopolitical and interstate conflicts	n 1981, Saddam ordered the construction to begin—urged on, according to another former senior Iraqi official, by the military situation. (The official, who lives in Baghdad, spoke to me on condition of anonymity, fearing that he would lose his pension if he spoke out.) A year before, Saddam had launched a huge invasion of Iran, hoping to seize its oil fields and possibly to overthrow its government	Filkins (2016)
Geopolitical and interstate conflicts	Iraq’s leaders feared that they were due for another flood, which would strand the Army. “It was of the utmost importance to begin construction of the dam as quickly as possible,” the official said	Filkins (2016)

Geopolitical and interstate conflicts	The Trevi engineers, scrambling to keep the dam functioning, are operating in a militarized environment. Hundreds of Italian and Kurdish soldiers patrol the area, on alert for an attack. In September, the Italian media reported that fighters were preparing an operation to recapture the dam. The following month, Kurdish forces fired a missile at a team of ISIS commandos who were approaching with a load of explosives	Filkins (2016)
Geopolitical and interstate conflicts	When isis fighters took the dam, in 2014,	Filkins (2016)
Geopolitical and interstate conflicts	ISIS militants captured Iraq's largest dam on Thursday, wresting it away from the Kurdish Peshmerga's control	Bender (2014)
Geopolitical and interstate conflicts	La scorta del cantiere L'ufficio stampa dello stato maggiore della difesa però precisa che i soldati italiani presso la diga non partecipano ad azioni offensive; hanno la sola missione di garantire la sicurezza della ditta italiana che ha ottenuto la commessa per mettere in sicurezza la diga omonima, sul fiume Tigri. In altre parole, l'esercito italiano farà la scorta armata a un cantiere civile.	Forti (2016)
Geopolitical and interstate conflicts	nel 1981 le monarchie del golfo finanziarono la costruzione della diga (allora chiamata "Saddam") per permettere una maggiore produzione di energia elettrica e un'irrigazione più efficiente del nord del paese, sperando che ciò aiutasse l'Iraq a far fronte alla guerra.	InfoAut (2016)
Geopolitical and interstate conflicts	In Uzbekistan, cotton accounts for some 60% of foreign exchange receipts and provides about 45% of employment. The reservoir could take as long as 18 years to fill and during this period, water flow downstream would be reduced. Therefore, Uzbekistan argues the Rogun dam would significantly reduce the amount of water reaching Uzbekistan	Skoba (2013)
Geopolitical and interstate conflicts	Relations are described as constantly strained due to the common rivers, as well as Tajikistan's dependence on Uzbekistan for transit of energy and various goods. In April 2012, Uzbekistan cut gas deliveries to Tajikistan for two weeks. Earlier, Uzbekistan had been known to stop rail traffic to Tajikistan, damaging the trading relationship between the countries.	Skoba (2013)
Geopolitical and interstate conflicts	the potential impacts on downstream nations such as Uzbekistan are a significant source of controversy	Human Rights Watch (2014)
Impacts - environmental disruption		
Emission of greenhouse gases	The Nile basin is also going to be severely affected by the impacts of climate change (Swain, 2011). Flooding 168,000 hectares will result in decomposition of vegetation, leading to emissions of carbon dioxide and methane gases. These greenhouse gases contribute to climate change. Grand	International Rivers (2017)

Emission of greenhouse gases	El impacto medioambiental se agrava por los gases tóxicos y de efecto invernadero que produce la putrefacción de la materia orgánica en el río, pues éstos generan una alteración en el clima. De acuerdo a la comunidad, la temperatura de la zona presenta cambios drásticos	CENSAT-AguaViva (n.d.)
Biodiversity loss	The area of river to be flooded contains about 170 islands, approximately 80% of which will be submerged by the reservoir. The islands and the forests along the Caroni river represent a unique and scarce habitat for several species.	Lang et al. (2000)
Biodiversity loss	The dam was partly funded by the World Bank. The floodplain of the Kafue river was once one of the richest wildlife habitats in the world. The Itezhitezhi dam was built upstream of the Batoka Gorge dam and prevented the remaining seasonal floods of the Kafue river, thus effectively wiping out the habitat.	Lang et al. (2000)
Biodiversity loss	when the first turbine was opened in 1994, more than 120,000 dead fish were found downstream, thought to have been killed by the lack of oxygen in the reservoir caused by rotting vegetation	Lang et al. (2000)
Biodiversity loss	Yam production in the floodplain area fell by 100,000 tonnes after the dam's completion, while downstream fish stocks fell by 60-70%	Lang et al. (2000)
Biodiversity loss	Es la mortandad de peces más grande en Colombia por esa causa, según el Ministerio	Eliécier Quintero (2007)
Biodiversity loss	Se destruyeron 842 hectáreas de bosque ripario y tropical seco y las epifitas, poblaciones de peces que son básicos para la seguridad alimentaria, especies de aves, de reptiles y tres especies de mamíferos en severo peligro: la pacaraná, el mono nocturno de manos grises y la nutria neotropical.	Dussán Calderón (2016)
Biodiversity loss	Elimination of the riverine forest and woodland, due to at least a 57% to 60% reduction of river flow volume, with accompanying destruction of forest biodiversity	ARWG (2009)
Biodiversity loss	En los primeros días, cuando comenzó a desviarse el río, se registró una alta e incontrolada mortandad de peces	Roa Avendaño and Duarte Abadía (2012)
Biodiversity loss	Tigrillos, armadillos, reptiles y osos se han encontrado, según testimonios de habitantes de la zona, muertos o heridos sobre las carreteras.	Roa Avendaño and Duarte Abadía (2012)
Biodiversity loss	En el río ha existido una alta diversidad de fauna íctica, los pobladores locales mencionan algunos de ellos: bocachico, dorada, blanquillo, comelón, capaz, golosa, chocas, boroncoro, lamprea, titeto, hocicón, capitán, tierra loca, bagre, e incluso caimanes y rayas. Actualmente, las actividades tradicionales de subsistencia son desplazadas por los daños ecológicos y por la	Roa Avendaño and Duarte Abadía (2013)

	disminución de la oferta de bienes naturales del río. El pescado escasea en la región y lo que se pesca se consume con el riesgo de estar contaminado	
Biodiversity loss	The more than $1 billion project would create a 57-square-mile reservoir by damming two large glacial rivers flowing from Europe's largest glacier, the Vatnajoekull. The dams would destroy a large portion of Iceland's highlands, including the Dimmugljufu canyon as well as several protected areas and important nesting grounds for reindeer and pink-footed geese. Iceland's Central Highlands make up one of Europe' largest (previously) pristine wilderness areas. Additional environmental impacts will be felt due to the hydrological changes wrought by the dam's construction and operation. These include, but are not limited to, increased erosion, changes in water temperature and turbidity that would impact aquatic life, and destruction of habitat, especially breeding grounds and migration routes	GNAD (2011)
Biodiversity loss	Loss of wildlife habitat around dam, powerhouse sites and nearby areas.	Thanju (2008)
Biodiversity loss	"Fish is therefore largely disappearing from the lake," Gunnar explained.	IRO (2013)
Biodiversity loss	Gunnar stated that farmlands and natural relics are at risk, islets and banks to the north of the bridge. "You can already see the impacts on the diversity of this very beautiful bird paradise. It pains me to witness this," Gunnar, who owns the land Egilsstaðir I, to which the islets belong, remarked.	IRO (2013)
Biodiversity loss	Today there is virtually no big game left on the Zambian side of the reservoir.	Scudder (2005)
Biodiversity loss	In spite of its small size, though, the project has had drastic impacts on biodiversity. The Kihansi Gorge ecosystem is one of only 25 Global Biodiversity Hotspot as designated by IUCN. It is home to numerous endemic flora and fauna species that lived in the spray of the gorge's 800– meter–high waterfall. This waterfall was destroyed by the dam, which collects water above the gorge, diverts it into a series of tunnels running into and out of the power plant, and returns the water to the river at the bottom of the gorge, 6 km downstream. The loss of the spray from the waterfall, which was laden with mineral–rich silt, has sent the critically endangered Kihansi Spray Toad and at least two endangered plant species to the brink of extinction. The previously unknown Kihansi Spray Toad exists nowhere else on earth, and the dam has already destroyed over 90% of its habitat. Efforts to rescue the toad through a captive breeding program and the installation of a sprinkler system in the gorge have failed to stabilize population levels.	International Rivers (2001)
Biodiversity loss	It was only found in the Kihamsi Falls area of Tanzania, but its population had crashed in recent years from a high of an estimated 17,000 individuals.<br>Conservationists suggest that the rapid decline was primarily the result of of a dam being	BBC News (2009)

	constructed upstream from the toads' habitat, which resulted in a 90% reduction in the flow of water.	
Biodiversity loss	Endangered Species: The Maloti minnow, rock catfish and the bearded vulture are some of the known rare and endangered species that are losing habitat to the project. A threatened endemic plant, the Spiral Aloe, will be affected by the project, as will rare bird species that nest in the area. Since no studies were done before construction began on Katse Dam, it is unknown whether the Maloti minnow was found in streams and rivers in the area. LHDA reports that the minnow has been found in at least seven sites in the catchment area of Mohale Dam. The mitigation measures for the minnow include an untried captive breeding program, as part of an effort to restock the fish in appropriate habitat. However, there are plans to introduce trout for recreational fishing in the reservoir, which the EIA acknowledges would "eliminate through predation" remaining populations of the minnow in the watershed. In addition, the umbraculate frog – an important indicator species for water quality – will "disappear from the area," according to the EIA.	International Rivers (2005)
Biodiversity loss	these impacts include reductions in wetlands habitat, less water available downstream for people and wildlife, reductions in fisheries,	International Rivers (2005)
Biodiversity loss	El principal impacto del aprovechamiento de Piedra del Águila consistió en la pérdida de un tramo de aproximadamente 100 km del valle del río Limay y parte del valle del río Collón Curá. Esta zona se caracterizaba por la gran variedad y diversidad de vegetación y fauna silvestre, así como también por poseer una corriente de agua de gran calidad.	Balazote and Radovich (2003)
Biodiversity loss	Impacts on biodiversity are a key concern of the project as it will cause irreversible damage.	Green Alternative (2012)
Biodiversity loss	Reduction in flow of the Lower Kihansi River, Tanzania, caused by implementation of a hydropower project in May 2000 has the potential to lead to changes in vegetation composition of spray maintained wetlands. These wetlands are the only known habitat for the Kihansi Spray Toad, Nectophrynoides aspergini	Quinn et al (2005)
Biodiversity loss	The industrialization of Iceland's natural resources will not only devastate vast landscapes of great natural beauty and scientific importance, but impair species such as reindeer, seals and fish, and the already endangered pink-footed goose and several other bird species.	Saving Iceland (2005)
Biodiversity loss	The surface of Hálslón, the reservoir by Kárahnjúkar dams in Iceland's eastern highlands, is constantly rising, swallowing nests and eggs laid by geese. The area is the nesting ground for greylag geese amongst numerous other species of rare and endangered birds. At least 500 greylag nests are thought to be at risk.	Saving Iceland (2007)

Biodiversity loss	Because of the lack of studies on the project's impacts, some of the damage caused by the dam is only now becoming apparent, including the possibility that water from the reservoir may be seeping into Argentina's Iberá wetlands, destroying the region's rich biodiversity.	International Rivers (n.d./a)
Biodiversity loss	In 1994, Yacyretá's floodgates were closed and the reservoir partially filled, flooding river islands that were home to endemic species, decimating fish populations,	International Rivers (n.d./a)
Deforestation	the banks of the reservoir have been heavily deforested and transformed into a desert due to the fragility of the terrain that continues to slide down into the basin. A number of attempts to replant trees have been unsuccessful, perhaps because they came too late (started in 1991).	Colajacomo (1999)
Deforestation	Se destruyeron 842 hectáreas de bosque ripario y tropical seco	Dussán Calderón (2016)
Deforestation	Inundación de 7482,4 hectáreas de la Reserva Forestal de la Amazonía, afectando el Bosque Seco Tropical	Galindo Vanegas (2018)
Deforestation	The dam will flood 1,680 square kilometers of forest in northwest Ethiopia (an area about four times the size of Cairo),	International Rivers (2014)
Deforestation	Questo causerà l'inaridimento di molte aree a riva ed farà scomparire la foresta ripariale.	Survival International (2019)
Deforestation	incremento en la tasa de deforestaciones	Roa Avendaño and Duarte Abadía (2013)
Deforestation	Environmental impacts Major impacts occurred due to the implementation of the project on physical, biological and socioeconomic and cultural environment are summarized below: […] Submergence of forest land and other land uses due to creation of 5.3 km long (65 ha) reservoir.	Thanju (2008)
Deforestation	Removal of 6,093 trees of various species (khayar, bakaino, ipil-ipil, simal, sissoo and sal) due to implementation of the project.	Thanju (2008)
Deforestation	environmental concerns raised in relation to the Rogun dam include further desertification of the region as well as the risk of flooding and accidents. The	SKOBA (2013)
Disruption of river ecology and water contamination	At one point, one-fifth of the water surface was clogged with aquatic plants.	Lang et al. (2000)
Disruption of river ecology and water contamination	se ha iniciado de inmediato un proceso de eutrofización de las aguas.	Gerebizza (2009)

Disruption of river ecology and water contamination	un tercio de las 27mil hectáreas del embalse, ha sido cubierto por una planta acuática invasora, la Eichornia crassipes (Jacinto de agua), conocida por las poblaciones locales como “lechuguin”, que por su rápido crecimiento y velocidad de multiplicación, constituye un grave problema ambiental. Sus largas raíces bajo la superficie, que pueden llegar a tener hasta un metro de longitud, se marchitan rápidamente causando emisiones nocivas y contaminando el agua.	Gerebizza (2009)
Disruption of river ecology and water contamination	La falta de planificación se evidenció en la necesidad de construir con urgencia plantas de tratamiento de aguas residuales para los municipios circunvecinos, como una medida de prevención de la contaminación de las aguas del embalse.	Galindo Vanegas (2018)
Disruption of river ecology and water contamination	il y a une probabilité de 60 % que l’événement u les eaux de la retenue de Garafiri sont eutrophes » se produise. Or, l’eutrophisation peut avoir de lourdes conséquences sur le plan environnemental et économique, de par la désoxygénation des eaux et la production de composés toxiques et corrosifs (ammoniaque et hydroxyde sulfuré entre autres).	Garafiri_39850105
Disruption of river ecology and water contamination	Grand Renaissance is located in a region with extremely high temperatures and low precipitation. Potential evaporation rates are very high. The evaporation losses from the dam’s reservoir could be as high as three billion cubic meters per year (Zehabesha, 2012). The water losses will reduce the overall water flow that reaches the downstream Nile (Water Diplomacy Aquapedia, 2014).	International Rivers (2017)
Disruption of river ecology and water contamination	One hydrologist estimates that the reservoir could evaporate 3bn cubic meters of water a year – three times Egypt’s annual rainfall, and enough to meet the basic needs of up to half a million people.	International Rivers (2014)
Disruption of river ecology and water contamination	The project proposes an artificial flood to mitigate these impacts. However, the proposed flood would last only 10 days, while the natural flood builds gradually over several months, until it peaks in August or September. A truncated 10-day flood would not reach all the areas now nurtured by annual flooding, and would likely fall far short of supporting current agricultural productivity.	International Rivers (2009)
Disruption of river ecology and water contamination	During the initial period of dam/reservoir filling, the lake level would drop up to 1–2m (95% confidence interval)	Velpuri and Senay (2013)
Disruption of river ecology and water contamination	Secondo gli esperti la riduzione del flusso del fiume causerà l’abbassamento del livello del lago Turkana di circa due terzi. Questo distruggerà la riserve ittiche da cui dipendono centinaia di migliaia di indigeni.	Survival International (2019)

Disruption of river ecology and water contamination	Secondo numerosi esperti indipendenti, la diga, le piantagioni e i canali di irrigazione avranno un enorme impatto sui delicati ecosistemi della regione modificando le esondazioni stagionali del fiume Omo e riducendone drammaticamente il volume	Survival International (2019)
Disruption of river ecology and water contamination	Sin embargo, el daño al río es aún más grave. De acuerdo al estudio que realizaron investigadores de la Universidad Santo Tomás, aguas abajo –las aguas que están después del muro de la presa–, la saturación de oxígeno arrojó resultados inferiores al 18%, lo	CENSAT-AguaViva (n.d.)
Disruption of river ecology and water contamination	Un estudio de la Universidad Santo Tomas, liderado por el reconocido ambientalista e ingeniero químico Jairo Puente, concluyó que en las aguas del Sogamoso es grave la falta de oxígeno.	Roa Avendaño (2016)
Disruption of river ecology and water contamination	alteración de la calidad del agua.	Roa Avendaño and Duarte Abadía (2012)
Disruption of river ecology and water contamination	Según las comunidades, fueron permanentes los vertimientos de químicos y lodos durante la construcción del túnel y de ahí que el río mantuviera durante meses con una alta carga de sedimentos. Además, han sido varios los derrames de contaminantes sobre las quebradas y el río durante la construcción de la obra, los cuales han sido denunciados por los pobladores locales.	Roa Avendaño and Duarte Abadía (2012)
Disruption of river ecology and water contamination	The more than $1 billion project would create a 57-square-mile reservoir by damming two large glacial rivers flowing from Europe's largest glacier, the Vatnajoekull. The dams would destroy a large portion of Iceland's highlands, including the Dimmugljufu canyon as well as several protected areas and important nesting grounds for reindeer and pink-footed geese. Iceland's Central Highlands make up one of Europe' largest (previously) pristine wilderness areas. Additional environmental impacts will be felt due to the hydrological changes wrought by the dam's construction and operation. These include, but are not limited to, increased erosion, changes in water temperature and turbidity that would impact aquatic life, and destruction of habitat, especially breeding grounds and migration routes	GNAD (2011)
Disruption of river ecology and water contamination	Hydrological changes in the 13 km stretch downstream between the dam and the confluence with the major tributary, Badi Gad.	Thanju (2008)
Disruption of river ecology and water contamination	With the dam's establishment, Jökulsá á Dal was made to flow into Lagarfljót and glacial sediment increases the water's turbidity or cloudiness.	IRO (2013)

Disruption of river ecology and water contamination	"Cahora Bassa has wrought massive geomorphological and ecological changes on the system" with actual changes "far worse" than even he had predicted in the 1970s	Scudder (2005)
Disruption of river ecology and water contamination	flood recession agriculture has been greatly curtailed since flooding only rarely inundates flood plains.	Scudder (2005)
Disruption of river ecology and water contamination	In the delta, Davies estimates a 40-45 percent loss of mangroves. Outlying channels have been clogged with aquatic vegetation that is no longer flushed out, while the productivity of former grasslands for wildlife has been reduced by the invasion of woody plants. In the main river, fish stocks have declined, while changes in vegetation suggest "saline wedge intrusion" (ibid).	Scudder (2005)
Disruption of river ecology and water contamination	Kariba's construction drastically altered, and regularized, the natural regime of the Zambezi. Thereafter low season flows increased, while flood water flows decreased.	Scudder (2005)
Disruption of river ecology and water contamination	They lamented over the fact that after the Kpong Dam was constructed, flow rate of water below the dam dropped drastically leading to the seizure of seasonal flooding and recession regimes of the Volta River.	Owusu et al (2016)
Disruption of river ecology and water contamination	impacts of altered river regime on downstream ecosystems and resource users is uncertain and unpredictable," but notes that water quality below the dam will be altered – including lower sediments, oxygen levels and nutrients and a change of water temperature.	International Rivers (2005)
Disruption of river ecology and water contamination	The effects of diverting most of the river's flow below the dams are substantial. Basically, these impacts include reductions in wetlands habitat, less water available downstream for people and wildlife, reductions in fisheries, and cessation of flooding. The hydrological variability of these floods is critical to many species found in ecosystems downstream of the LHWP – a variability that will cease with the damming of the waters.	International Rivers (2005)
Disruption of river ecology and water contamination	El principal impacto del aprovechamiento de Piedra del Águila consistió en la pérdida de un tramo de aproximadamente 100 km del valle del río Limay y parte del valle del río Collón Curá. Esta zona se caracterizaba por la gran variedad y diversidad de vegetación y fauna silvestre, así como también por poseer una corriente de agua de gran calidad.	Balazote and Radovich (2003)
Disruption of river ecology and water contamination	The scheme will considerably reduce the environmental flow of the Nakra river, leaving just 10 per cent of average annual flow (AAF) downstream regardless of seasonal fluctuations. Moreover, environmental flow rate has been defined on 5% of AAF (0.85 m3 per second) downstream of the Nenskra Dam	Green Alternative (2012)
Erosion and sedimentation	erosion (the coasts of neighbouring Togo and Benin being lost at the rate of 10m a year)	Lang et al. (2000)

Erosion and sedimentation	Soil erosion, already a major problem, has been aggravated by the construction of the dams and will be worsened still further as displaced villagers are forced to cultivate and overgraze steeper hillsides.	Lang et al. (2000)
Erosion and sedimentation	The trapping of silt behind the dam has also led to severe coastal erosion downstream, with beaches and sections of the highway along the West African coast from Ghana to Nigeria being washed away.	Lang et al. (2000)
Erosion and sedimentation	Ahora se registran fallas y derrumbes que afectan a la población y hacen que aumente el nivel del sedimentación del embalse y, por tanto, su cota máxima"	SEMANA (2019)
Erosion and sedimentation	Ethiopia's highlands are one of the most erosion-prone places on earth, and sedimentation of the reservoir is a big risk for the dam's power output and lifespan (International Rivers, 2013). The mountainous terrain of Ethiopia is highly fragile in terms of slope stability, which may easily lead to landslides and slope failures (Swain, 2014).	International Rivers (2017)
Erosion and sedimentation	ha crecido la cantidad de sedimentos que transporta el río, presentándose caudales muy fuertes que erosionan los límites del cauce	Roa Avendaño and Duarte Abadía (2012)
Erosion and sedimentation	The more than $1 billion project would create a 57-square-mile reservoir by damming two large glacial rivers flowing from Europe's largest glacier, the Vatnajoekull. The dams would destroy a large portion of Iceland's highlands, including the Dimmugljufu canyon as well as several protected areas and important nesting grounds for reindeer and pink-footed geese. Iceland's Central Highlands make up one of Europe' largest (previously) pristine wilderness areas. Additional environmental impacts will be felt due to the hydrological changes wrought by the dam's construction and operation. These include, but are not limited to, increased erosion, changes in water temperature and turbidity that would impact aquatic life, and destruction of habitat, especially breeding grounds and migration routes	GNAD (2011)
Erosion and sedimentation	Soil erosion and land slides due to project construction works.	Thanju (2008)
Erosion and sedimentation	In the past months, Landsvirkjun, the national power company, has presented reports on erosion of the banks of Lagarfljót and other consequences of the glacial river's rechanneling.	IRO (2013)
Erosion and sedimentation	The erosion is more extensive and the water level higher than what all mathematical models indicated.	IRO (2013)
Erosion and sedimentation	"Tunneling for hydropower project using the drill and blast technique in a fragile region like the Himalayas seems to be definitely having geological and hydrological impacts both of which need to be assessed urgently. Kinnaur is the second most landslide prone district in the state	Himdara (2015)

	of Himachal. This is also a highly seismic zone. Despite this the apathy of the government and the blind push for hydro is cos~ng the local people”,	
Erosion and sedimentation	landslides, rock falls and slope destabilisation had occurred all over the roads and in villages due to the construction acitivity for hydropower development in	Himdara (2015)
Loss of protected, sacred or archaelogical sites	The reservoir will also flood 100 archaeological sites where 10,000 year-old relics, and objects from the Song Dynasty (900-1279 AD), have been found.	Lang et al. (2000)
Loss of protected, sacred or archaelogical sites	Inundó 8586 hectáreas de las cuales el 95% hacen parte de la Reserva Forestal Protectora de la Amazonía y del Macizo Colombiano	Dussán Calderón (2016)
Loss of protected, sacred or archaelogical sites	Y allí hay mucho hallazgo arqueológico , porque una zona arqueológicamente rica tiene se habían registrado más 78 lugares muy importante y se encontraron un cementerio indígena	Interview
Loss of protected, sacred or archaelogical sites	Inundación de 7482,4 hectáreas de la Reserva Forestal de la Amazonía	Galindo Vanegas (2018)
Loss of protected, sacred or archaelogical sites	Lake Turkana National Parks in Kenya has been placed on the List of World Heritage in Danger today at UNESCO’s World Heritage Committee meeting, due to impacts from the Gibe III dam	IUCN (2018)
Loss of protected, sacred or archaelogical sites	Il fiume attraversa i parchi nazionali Mago e Omo e, nel 1980, il suo bacino è stato inserito nell’elenco dei Patrimoni dell’Umanità dell’Unesco per la sua particolare importanza geologica e archeologica	Survival International (2019)
Loss of protected, sacred or archaelogical sites	Nel giugno 2018, l’UNESCO ha inserito il Lago Turkana nella Lista dei Patrimoni dell’Umanità in Pericolo.	Survival International (2019)
Loss of protected, sacred or archaelogical sites	The more than $1 billion project would create a 57-square-mile reservoir by damming two large glacial rivers flowing from Europe's largest glacier, the Vatnajoekull. The dams would destroy a large portion of Iceland's highlands, including the Dimmugljufu canyon as well as several protected areas and important nesting grounds for reindeer and pink-footed geese. Iceland's Central Highlands make up one of Europe' largest (previously) pristine wilderness areas. Additional environmental impacts will be felt due to the hydrological changes wrought by the dam's construction and operation. These include, but are not limited to, increased erosion, changes in water temperature and turbidity that would impact aquatic life, and destruction of habitat, especially breeding grounds and migration routes	GNAD (2011)

Loss of protected, sacred or archaelogical sites	The more than $1 billion project would create a 57-square-mile reservoir by damming two large glacial rivers flowing from Europe's largest glacier, the Vatnajoekull. The dams would destroy a large portion of Iceland's highlands, including the Dimmugljufu canyon as well as several protected areas and important nesting grounds for reindeer and pink-footed geese. Iceland's Central Highlands make up one of Europe' largest (previously) pristine wilderness areas. Additional environmental impacts will be felt due to the hydrological changes wrought by the dam's construction and operation. These include, but are not limited to, increased erosion, changes in water temperature and turbidity that would impact aquatic life, and destruction of habitat, especially breeding grounds and migration routes.	GNAD (2011)
Loss of protected, sacred or archaelogical sites	The growing reservoir also displaced as many as 40,000 people, drowning 94 inhabited villages and an unknown number of archaeological sites (Öktem, 2002: 315).	Stahl (2019)
Loss of protected, sacred or archaelogical sites	direct impact on the proposed protected areas – the Upper Svaneti National Park and the Upper Svaneti Protected Landscape – thus undermining conservation efforts.	Green Alternative (2012)
Impacts | socio-economic disruption		
socio-economic disruption	Desde cuando comenzaron a aparecer peces muertos, la industria ha tenido pérdidas por unos 4.000 millones de pesos.	Eliécier Quintero (2007)
socio-economic disruption	Desde la vereda Media Estancia, en donde yo vivo, hacer ese trayecto se vuelve exageradamente largo. Eso promueve aspectos como el desapego social y el estancamiento económico del municipio.	SEMANA (2019)
socio-economic disruption	Gustavo Martínez, exconcejal de Macanal, consideró, entre tanto, que en esa población ya no es posible adelantar agricultura limpia porque los campesinos deben acudir a químicos para poder sacar, al menos, los productos de su pancoger	SEMANA (2019)
socio-economic disruption	Los agricultores también aseguran haber sufrido por esta presa. Aseveran que el clima cambió y que sus cultivos tradicionales se han visto afectados por cuenta de la aparición de plagas, hongos y microorganismos que dañan los frutos y el suelo. “El hielo que produce este cuerpo de agua, especialmente en las noches y las madrugadas, acabó con los cultivos de café, papaya, guayabos, lulo y tomate, que antes se daban en esta región. Ahora nos toca vivir de otras cosas como la pesca y la minería”,	SEMANA (2019)
socio-economic disruption	A casi 30 años de la construcción de estas dos grandes obras de infraestructura, aproximadamente 50.000 personas, buena parte de las cuales permanecieron aisladas al interior del dique artificial creado por la represa de Daule Peripa, todavía sufren sus impactos.	Gerebizza (2009)

socio-economic disruption	egún las organizaciones de la sociedad civil ecuatoriana que trabajan en el territorio, se habla de casi 50.000 personas afectadas directa e indirectamente por el dique artificial.	Gerebizza (2009)
socio-economic disruption	Esas ventas y compras han producido cambios en la valoración de las tierras, situación que ha generado tensiones en la comunidad.	Roa Avendaño and Duarte Abadía (2012)
socio-economic disruption	la fase de construcción del proyecto cambia drásticamente los modos de vida de las personas.	Roa Avendaño and Duarte Abadía (2012)
socio-economic disruption	the project owners original ly ignored the consequences the project would have on the 5,000 to 6,000 Cree and 3,500 Inuit whose fishing, hunting and trapping traditions would be disrupted	Wall (2017)
socio-economic disruption	Impacts to indigenous Bote (fisherman) community. About 21 houses, 5 cowshed and approximately 13 ropani of land from the Bote families were acquired.	Thanju (2008)
socio-economic disruption	Conflits entre les paysans de même village	Raphaël et al (2019)
socio-economic disruption	Conflits inter-villages	Raphaël et al (2019)
socio-economic disruption	faced with land scarcity after more than 40 years, we are now witnessing conflicts of all kinds, particularly between natives and their hosts.	Raphaël et al (2019)
socio-economic disruption	approximately 152,000 Lesotho villagers living within the Senqu River basin below the Katse and Mohale Dams would be adversely affected to varying degrees by LHWP.	Lenka Thamae and Pottinger (2006)
socio-economic disruption	For the last one and a half year the administra~on has not been able to restore connectivity to nearby towns and villages as the road here (near Tapri) still remains blocked	Himdara (2015)
socio-economic disruption	Investigations by the World Bank and Inter–American Development Bank have shown that authorities of the bi–national company EBY are unable to handle the devastating social dislocation that raising the reservoir would cause.	International Rivers (n.d./a)
Accidents	In 1999, at least 39 people were killed and thousands were left homeless after floodgates were opened to release rising floodwaters at the Kainji, Jebba and Shiroro dams	Lang et al. (2000)
Accidents	41 dead.	Scudder (2005)
Accidents	81 people were reported dead.	Scudder (2005)
Accidents	The actual cause of the mystery deaths remains unknown. Based on the symptoms, the clustering of deaths toward the end of the dry season, and the age and sex of the victims, the most likely cause was consumption of toxic plants gathered by women and children during periods of food scarcity.	Scudder (2005)

Displacement & Resettlement	35,000 people were evicted when the reservoir was flooded.	Lang et al. (2000)
Displacement & Resettlement	Construction is planned to be completed in 2002. To make way for the reservoir, 180,000 people will be evicted from their homes and about 300,000 people will be affected by the project.	Lang et al. (2000)
Displacement & Resettlement	Environmentalists have long claimed that the dam violates World Bank environmental standards and procedures for resettlement of local populations.	Lang et al. (2000)
Displacement & Resettlement	Some 96,000 people were displaced by the project and are still fighting in the courts for compensation	Lang et al. (2000)
Displacement & Resettlement	The Kainji dam was completed in 1968 and caused the displacement of 44,000 people.	Lang et al. (2000)
Displacement & Resettlement	Su construcción, con la inundación de 641 hectáreas, significó el traslado de 126 personas o 46 familias, dentro de las cuales la mayoría se encontraban bajo la línea de la pobreza. Algunas de ellas, incluso, ya habían enfrentado un proceso de relocalización cuando se construyó la Central Pangue.	Ecosistemas (2014)
Displacement & Resettlement	about 120 long-established agricultural communities that were submerged by the reservoir of the Tarbela Dam. Around 96,000 people were displaced as a consequence of the dam, reservoir, and associated infrastructure, which was 20 percent more than originally estimated.	Bennet and McDowell (2012)
Displacement & Resettlement	the Bujagali dam affected 3,190 households or 13,760 individuals	NAPE (2014)
Displacement & Resettlement	Después de haber perdido casa, tierra y animales, muchas familias se vieron forzadas a emigrar a otras zonas del País, la mayor parte hacia la ciudad de Guayaquil.	Gerebizza (2009)
Displacement & Resettlement	Altogether 1,468 families lost their land (or part of it), their houses, or both, out of which 263 families were defined as SPAF2 and 1,205 families as PAF3	Thanju (2008)
Displacement & Resettlement	Cash compensation for the loss of land, house and other assets. The formal tenants of guthi (communal) land were paid an additional 42% compensation, totaling 75% vis-à-vis legal provision of only 33% compensation. Compensation also provided for standing crops and grass damaged during the project construction.	Thanju (2008)
Displacement & Resettlement	El llenado del embalse significó el traslado de 126 personas, algunas de las cuales ya habían sido relocalizadas durante la construcción de la central Pangue en 1996, y estando la mayor parte de ellas bajo la línea de la pobreza.	ElDesconcierto (2014)
Displacement & Resettlement	Cuando se produjo la inundación, vivían en el paraje aproximadamente 70 pobladores. La afectación sufrida por esta comunidad consistió en la inundación del 32,3% de las tierras de su propiedad y la pérdida total de las viviendas e instalaciones construidas.	Balazote and Radovich (2003)

Displacement & Resettlement	The dam will create a massive reservoir that will displace an estimated 7,000 families (about 42,000 people) by the time the project is completed. Between 2009 and early 2014, the government has already resettled approximately 1,500 families out of the reservoir zone to several other locations in Tajikistan.	Human Rights Watch (2014)
Displacement & Resettlement	In 1990 around 16,000 people were living in the project area. 380 families had to be relocated.	KFW (2006)
Displacement & Resettlement	expulsion of 15,000 people from their homes	International Rivers (n.d./a)
Forced or violent displacement	About 80,000 farmers were forced to move to make way for the 8,500 square kilometre Volta reservoir — the largest in the world	Lang et al. (2000)
Forced or violent displacement	In 1958, police of the then British colony of northern Rhodesia shot dead eight villagers and wounded over 30 more during the evictions	Lang et al. (2000)
Forced or violent displacement	In 1999, at least 39 people were killed and thousands were left homeless after floodgates were opened to release rising floodwaters at the Kainji, Jebba and Shiroro dams	Lang et al. (2000)
Forced or violent displacement	Less than 25% of the 50,000 people who would be forced to move to make way for the Yacyreta's reservoir if fully filled have been resettled so far.	Lang et al. (2000)
Forced or violent displacement	A mi familia y a mí nos sacaron de allá a la fuerza.	SEMANA (2019)
Forced or violent displacement	Hubo presiones, intimidación y algunas expropiaciones	SEMANA (2019)
Forced or violent displacement	Según los habitantes de Macanal, la construcción del embalse también trajo consigo el desplazamiento forzado. "La gente se vio obligada a irse por cuenta del mismo Estado.	SEMANA (2019)
Forced or violent displacement	The filling of the basin began in January 1983 right after the final massacre. The people had to start moving away and the Rio Negro village was abandoned. They took refuge in the mountains and of them " a non precised number of men, women and children died because of the forced transfer" 8. Some remained in the mountains for five years and only after the first amnesty, announced by General Mejia Victores in 1985, they started to walk towards their resettlement village, Pacux, escorted by the army.	Colajacomo (1999)
Forced or violent displacement	Thirty years ago a massacre took place in the Guatemalan highlands that left 400 people dead. Countless more were displaced, tortured, raped or left starving. And all to make way for a hydroelectric dam.	Dearden (2012)
Forced or violent displacement	Construction proceeded without a comprehensive census of affected peoples or a plan to address compensation, resettlement and alternative livelihoods.	Johnston (2010)

Forced or violent displacement	Dam releases occurred with no warning and resulting flash floods destroyed crops, drowned livestock and sometimes killed people.	Johnston (2010)
Forced or violent displacement	river-basin communities were evicted through violent interventions and, in some instances, massacres.	Johnston (2010)
Forced or violent displacement	Violence associated with resettlement negotiations and forced displacement included the kidnapping, torture and deaths of four community leaders from two villages	Johnston (2010)
Forced or violent displacement	violence escalated into a series of massacres that, by September 1982, had resulted in the deaths of 444 of the 791 members of the Río Negro community	Johnston (2010)
Forced or violent displacement	When the reservoir waters rose in January 1983, ten communities in the Chixoy river basin had been destroyed by massacre, including the village of Río Negro. Any survivors were hunted down in the surrounding hills, and then forcibly resettled at gunpoint.	Johnston (2010)
Forced or violent displacement	después del llenado del embalse han sido obligados a abandonar sus propias tierras 14.965 campesinos,	Gerebizza (2009)
Forced or violent displacement	– sfollamento forzato di 44.ooo persone, che non hanno ricevuto l'indennizzo previsto dal progetto. Gli sfollati sono stati costretti a spostarsi in villaggi costruiti dalla stessa Impregilo s.p.a., senza accesso all'acqua ed alle infrastrutture di base;	CDCA (n.d.)
Forced or violent displacement	.. the military operation in question caused the death of more than 45 pastors<br>Mursi and 39 Bodi while their flocks were grazing. Many people have been driven away and their animals stolen by soldiers. From February 2015, more than 120 people were killed and 265 arrested and held in the Jinka prison. Three young, educated Mursi, who had played the role of representing their communities within the woreda, were fired on charges of having stirred up protests against resettlement.	Franchi and Manes (2016)
Forced or violent displacement	fueron desalojados a la fuerza por la policía	BHRRC (2017)
Forced or violent displacement	However, the organization singled out Iraq for what it says is an ambitious civil engineering project with the aim of deliberately draining the marshes. "If the world public knew that Saddam Hussein is about to destroy the last 10% of this antique garden of Eden, they might be as angry as I am and force them to stop somehow Tsaid Baroness Nicholson, speaking for the organization, which is involved with providing assistance to an estimated 95,000 South Iraqi Shias, including a large number of Marsh Arabs—also known as Madan—who are refugees in Iran. According to AMAR, prior to the Iraqi drainage project, about 400,000 people lived in the marshlands, which are historically rich farming areas; and about 50% were Marsh Arabs. Now, 30,000 people still live there.	Showstack (2012)

Forced or violent displacement	Armed soldiers ordered us to pack our belongings and leave the place immediately, telling us that the area within a 50-kilometer radius around the dam will be turned into a game park—hence the eviction order. They said the government did not have money for compensation but that was not going to stop the evictions	Human Rights Watch (2015)
Forced or violent displacement	authorities at Chingwizi camp misused humanitarian aid by coercing them to accept the government's resettlement plan.	Human Rights Watch (2015)
Forced or violent displacement	Before the government forcibly shut down the camp in mid-August 2014, United Nations agencies, international donors, and local groups provided humanitarian aid including shelter, food, clothing, potable water, health, and sanitary facilities. The Minister Bhasikiti issued a directive that all donations should be presented to his office for onward distribution to beneficiaries; donors were prohibited from directly giving out their donations. He said the directive would enable government to exercise greater control over the distribution of the aid.	Human Rights Watch (2015)
Forced or violent displacement	Flood victims said the government has subjected them to harassment, threats, physical violence, and used "cruel methods" to force them out of Chingwizi camp and onto onehectare plots in Bongo and Nyoni sections of Nuanetsi Ranch.	Human Rights Watch (2015)
Forced or violent displacement	Other steps taken to coerce flood victims, said Chingwizi camp leaders, include government officials directing groups working at Chingwizi camp to demolish camp toilets, close down the camp school and shut down its only clinic in early August 2014.	Human Rights Watch (2015)
Forced or violent displacement	The actual displacement took place during an emergency that some believe could have been avoided through efforts to prevent or minimize the floods.	Human Rights Watch (2015)
Forced or violent displacement	the central government have coerced the displaced to accept the one-hectare sites (instead of the five-hectare sites initially promised) with violence, harassment, and in some cases restricting access to water, food, and other essentials.	Human Rights Watch (2015)
Forced or violent displacement	The government used armed anti-riot police to forcibly move the flood victims from Chingwizi camp into resettlement on Nuanetsi ranch.	Human Rights Watch (2015)
Forced or violent displacement	They [government authorities] are now using food aid as a weapon to force me to occupy a one-hectare plot. They are not giving us food. If we complain they tell us to occupy the one-hectare plots in order to get food. The Assistant District Administrator … is the one telling us to move to onehectare plots in order to get food.	Human Rights Watch (2015)
Inadequate or no compensation measures	Around 20,000 people will lose some or all of their land because of the project. The land compensation is also delayed because the implementing agency, Pakistan's Water and Power Development Authority (WAPDA), has acquired less than half of the required new land.	Lang et al. (2000)

Inadequate or no compensation measures	have received no compensation.	Lang et al. (2000)
Inadequate or no compensation measures	Measures taken to help the 24,000 people who lost their farms, homes or access to communal grazing land as a result of Phase 1 of the LHWP have been heavily criticised as ineffective.	Lang et al. (2000)
Inadequate or no compensation measures	Some 96,000 people were displaced by the project and are still fighting in the courts for compensation	Lang et al. (2000)
Inadequate or no compensation measures	The 740 flooded villages were replaced by only 52 new ones. The reservoir rose faster than anticipated and many people were forced to flee, leaving behind their belongings for which they were never compensated.	Lang et al. (2000)
Inadequate or no compensation measures	The arid resettlement lands are far away from the reservoir.	Lang et al. (2000)
Inadequate or no compensation measures	Villagers also complain that compensation payments have been inadequate or remain unfulfilled. The compensation package provided for a lump payment for people who lost less than 1000 square metres of land. Those who lost more were to receive an annual delivery of corn for 15 years. Even LHDA health officers admit that the handouts are insufficient to • sustain the life of an individual• . In 1993, an LDHA survey revealed widespread dissatisfaction, the majority stating that the cash compensation did not reflect the productive value of the land. The payments also failed to take account of the loss of wild plants, fuel wood and building materials.	Lang et al. (2000)
Inadequate or no compensation measures	In 2000, nearly 25 years after displacement, the World Commission on Dams (WCD) found that many grievances over failures in the consultation and compensation process had still to be addressed.	Bennet and McDowell (2012)
Inadequate or no compensation measures	long delays in receiving alternative land and payments, which resulted in significantly reduced real value due to the devaluation of the rupee, and lack of employment and training opportunities.	Bennet and McDowell (2012)
Inadequate or no compensation measures	the strict rules disadvantaged smaller landowners whose property didn’t exactly fit the criteria	Bennet and McDowell (2012)

Inadequate or no compensation measures	then they released water without giving them proper compensation.	Bennet and McDowell (2012)
Inadequate or no compensation measures	Those with holdings of less than these limits were eligible only for cash compensation.	Bennet and McDowell (2012)
Inadequate or no compensation measures	We're all left without any resources. Compensation money has been consumed in building a home	Bennet and McDowell (2012)
Inadequate or no compensation measures	Issues of compensation and resettlement related both to the Bujagali Dam and its transmission lines have not been fully resolved	International Rivers (n.d)
Inadequate or no compensation measures	a total of 557 people affected by transmission line who felt that the compen-<br>sation given to them by the Uganda Electricity Transmission Company limited (UETCL) was not adequate took their case to court.	NAPE (2014)
Inadequate or no compensation measures	AES had committed itself to compensating all the project-affected people and had also promised other development projects and benefits. However AES left the project before promises were fulfilled.	NAPE (2014)
Inadequate or no compensation measures	AES promised communities a school but failed to honor their promise.	NAPE (2014)
Inadequate or no compensation measures	As we can see the review panel found the situation of the affected people to be less well off than before resettlement.	NAPE (2014)
Inadequate or no compensation measures	Missing payment for the contested land	NAPE (2014)
Inadequate or no compensation measures	The Bujagali dam Resettlement and Compensation Action Plan, and Community Development Action Plans was criticized by citizen groups locally and internationally for being shallow, shortsighted and focusing only on short-term impacts of the project.	NAPE (2014)
Inadequate or no compensation measures	The Bujagali project violated the lenders policies on resettlement and compensation.	NAPE (2014)

Inadequate or no compensation measures	The compensations given to people affected, however, were far below the current market rates.	NAPE (2014)
Inadequate or no compensation measures	when the dam and its related quarry were built, the local communities were not consulted, and remained uncompensated for land lost.	MAZZEI AND SCUPPA (2006)
Inadequate or no compensation measures	A la gente le hicieron pagos irrisorios; pagaban lo que ofrecía el Estado y punto. Y si no, la expropiaban. Nunca se dio chance de negociar.	SEMANA (2019)
Inadequate or no compensation measures	A muchos no les pagaron lo justo y a otros simplemente les tocó irse.	SEMANA (2019)
Inadequate or no compensation measures	La gente en medio de su inocencia tuvo que entregar sus tierras. Nunca se previó una reubicación como tal"	SEMANA (2019)
Inadequate or no compensation measures	During construction of the Chixoy dam, gravel and sand were removed from land shared by the villages of Pajales, Rio Negro and Xococ. Following protests from the locals, US$120,000 was given as compensation, but the money was divided unequally, causing conflicts among Rio Negro and Xococ communities.	Colajacomo (1999)
Inadequate or no compensation measures	The right to this common land has been lost and has not been compensated with the delimitation of protected areas for the benefit of affected people, as	Colajacomo (1999)
Inadequate or no compensation measures	Today, as former dictator Efraín Ríos Montt is brought to trial in Guatemala on counts of genocide, the survivors of the Chixoy massacre have still not received reparations	Dearden (2012)
Inadequate or no compensation measures	and in those few cases where compensatory agreements were achieved, formal documentation codifying communal rights was not provided.	Johnston (2010)
Inadequate or no compensation measures	Compensatory efforts were few and grossly inadequate to meet the basic needs of displaced communities, let alone provide redress for the full extent of lost land, property, communal resources, livelihoods and lives.	Johnston (2010)
Inadequate or no compensation measures	Construction began without legal acquisition of the land	Johnston (2010)

Inadequate or no compensation measures	Construction proceeded without a comprehensive census of affected peoples or a plan to address compensation, resettlement and alternative livelihoods.	Johnston (2010)
Inadequate or no compensation measures	Displaced communities lived in profound poverty, but because the utility was privatised and loans repaid in full, no mechanism within the utility or with international financiers existed for affected people to complain or negotiate assistance.	Johnston (2010)
Inadequate or no compensation measures	Downstream and upstream communities affected by the project have never been compensated for their losses	Johnston (2010)
Inadequate or no compensation measures	e compensazioni e il piano di re-insediamento erano a dir poco inadeguati.	Manes (2012)
Inadequate or no compensation measures	The 33 communities affected by the construction of the Chixoy Dam entered into negotiations with the Guatemalan government for integral reparations. The reparations package included an unprecedented budget for long-term community development. It was agreed to by President Colom, but he never officially signed and the reparations were not implemented.	GHRC (2011)
Inadequate or no compensation measures	A casi 30 años de la construcción de estas dos grandes obras de infraestructura, aproximadamente 50.000 personas, buena parte de las cuales permanecieron aisladas al interior del dique artificial creado por la represa de Daule Peripa, todavía sufren sus impactos. Por desgracia, no han recibido todavía compensación alguna.	Gerebizza (2009)
Inadequate or no compensation measures	año adicional por el cual ninguno ha sido resarcido.	Gerebizza (2009)
Inadequate or no compensation measures	De las entrevistas a algunos habitantes de las comunidades aisladas, se desprende que poquísimos campesinos han logrado, en el curso de los años, obtener compensación alguna por los daños sufridos.	Gerebizza (2009)
Inadequate or no compensation measures	– sfollamento forzato di 44.ooo persone, che non hanno ricevuto l'indennizzo previsto dal progetto. Gli sfollati sono stati costretti a spostarsi in villaggi costruiti dalla stessa Impregilo s.p.a., senza accesso all'acqua ed alle infrastrutture di base	CDCA (n.d.)
Inadequate or no compensation measures	Per la costruzione della diga sono stati sfollati circa 40.000 contadini, le cui terre sono state espropriate senza indennizzo a partire dall'inizio dei lavori di costruzione.	CDCA (n.d.)

Inadequate or no compensation measures	El pago de las compensaciones sociales, económicas y ambientales previstas en la licencia ambiental, como las que se omitieron en la misma o "no previstas".	Galindo Vanegas (2018)
Inadequate or no compensation measures	expropriation of land without compensation	Hodbod et al (2019)
Inadequate or no compensation measures	Three months ago, the government bulldozers destroyed my workshop. I went to the district administration demanding compensation and I presented my appeal to the project Omo-Kuraz Sugar coordination office in Hanna. No one has answered." The worst situations took place regarding the work of private companies. Local residents are forced to leave their homes and their lands by force, without receiving any compensation.	Franchi and Manes (2016)
Inadequate or no compensation measures	Las tierras que les adjudicaron a muchos resultaron infértiles, a otros los timaron entregándoles 3 hectáreas cuando les habían prometido 5 y a otros, como los pescadores y jornaleros de aguas arriba –las aguas que están antes del muro de la presa–, no los incluyeron en los planes de reparación y reubicación.	CENSAT-AguaViva (n.d.)
Inadequate or no compensation measures	En resumen, a las tierras se les adjudica un valor monetario, sin tener ningún estudio previo que pueda demostrar otras formas de valoración, especialmente relacionadas con los medios de subsistencia de los pobladores.	Roa Avendaño and Duarte Abadía (2012)
Inadequate or no compensation measures	los campesinos sin tierra, que al carecer de títulos de propiedad no son considerados en los planes de mitigación.	Roa Avendaño and Duarte Abadía (2012)
Inadequate or no compensation measures	the issue of reparations continues to be dealt with on an inadequate ad hoc, case by case, basis.	Scudder (2005)
Inadequate or no compensation measures	les villages t indemnisations promises aux paysans pour la perte de leu plantations n'ont toujours pas été versées et ne le seront v ment jamais. Alors que dans beaucoup d'autres villages, énergies humaines est dépensé en palabres (discussions) aujourd'hui, cinq années après le déménagement, à prop nisations des plantations de café inondées, dans les Tos, le c a refusé de rembourser systématiquement	PROWIZUR (1976)
Inadequate or no compensation measures	'absence des terres cultivables et les difficultés d'accès à la terre est relevée comme une autre cause importante de la pauvreté, surtout dans certains villages déplacés (AVB). Pour certaines populations déplacées les terres cultivables restent insuffisantes.	Pittalunga (2002)

Inadequate or no compensation measures	According to some of the respondents they never fully recovered from the damages caused by the floods and apparently, this has contributed to their present misery.	Owusu et al (2016)
Inadequate or no compensation measures	Many who lost homes to power–line construction in 1990–91, for example, still did not have replacement housing in October 1995, according to the World Bank.	International Rivers (2005)
Inadequate or no compensation measures	Distinta fue la situación de las familias Mapuches afectadas. Algunos fueron indemnizados y se asentaron en la cercana ciudad de San Carlos de Bariloche con resultados desastrosos. Es importante aclarar que estos grupos no eran propietarios de las tierras que ocupaban y, debido a ello, sólo recibieron como compensación un reducido monto indemnizatorio por las mejoras que en ellas habían realizado. Lo nefasto de esta política no se redujo solamente a la mínima cantidad de dinero percibida, sino también a la escasa experiencia de los grupos domésticos afectados en estrategias económicas centradas en la monetarización.	Balazote and Radovich (2003)
Inadequate or no compensation measures	La empresa Hidronor S.A., en relación a los efectos de la represa de Piedra del Águila sobre la comunidad, se limitó a discutir un monto indemnizatorio que recibiría el Estado provincial y que hasta el presente los afectados no han percibido.	Balazote and Radovich (2003)
Inadequate or no compensation measures	According to all resettled people Human Rights Watch interviewed, the government has not awarded compensation to any residents for this damage.	Human Rights Watch (2014)
Inadequate or no compensation measures	In addition to losing dekhan farmland, resettled families have been awarded significantly smaller household land plots. Over 85 percent of resettled families interviewed by Human Rights Watch stated that they had lost access to land or described feeling compelled to sell livestock due to lack of adequate land in the resettlement sites and the need to raise additional funds to finance home construction. Although multi-family households receive one plot per family, the size of each plot is such that a house occupies much of it, leaving little room for livestock or farming.	Human Rights Watch (2014)
Inadequate or no compensation measures	Most people told Human Rights Watch that they had to spend considerable amounts of their own money in order to build a house of similar size and quality to their former home.	Human Rights Watch (2014)
Inadequate or no compensation measures	most residents have waited for several years between the assessment and the time that they receive their first compensation payment.	Human Rights Watch (2014)

Inadequate or no compensation measures	significant delays between assessment and compensation, during which time the cost of construction materials increased significantly due to inflation.	Human Rights Watch (2014)
Inadequate or no compensation measures	The government does not have an on-the-ground monitoring or outreach system to assess resettled people's needs, to determine whether officials and agencies are fulfilling their duties, or to provide assistance to people in crisis.	Human Rights Watch (2014)
Inadequate or no compensation measures	The government has allocated land to displaced families, but it has not built houses for them on that land nor provided sufficient compensation to all families in accordance with international human rights standards to build new homes of a similar size and standard to those they previously owned.	Human Rights Watch (2014)
Inadequate or no compensation measures	As of February 2014, the government had compensated just 896 out of 6,393 families since work began on the dam 16 years earlier, according to local government Minister Ignatious Chombo. People should be able to be compensated and choose their residence and the government denied these rights.	Human Rights Watch (2015)
Inadequate or no compensation measures	Displaced families—the majority of which had not yet been financially compensated following property evaluations by the government prior to the floods<br>According to Minister —were told the government would only financially compensate them based on property evaluations "as soon as resources are available," at an undetermined time.	Human Rights Watch (2015)
Inadequate or no compensation measures	land at Chingwizi—they say—is not amenable to farming.	Human Rights Watch (2015)
Inadequate or no compensation measures	The government shut down the camp in August in an attempt to permanently relocate the families on a different part of Nuanetsi Ranch where each family was allocated a one-hectare plot of land. The families would now have significantly less land than they previously owned when they were in Masvingo.<br>Masvingo Provincial Affairs Minister Kudakwashe Bhasikiti, said the families, in their new Nuanetsi location, would only grow sugar cane for a planned government-owned ethanol project. The reasoning behind families being asked to grow sugar cane was that it would help the project achieve profitability quicker than if families were given option to grow other crops. Bhasikiti said that because the Tokwe-Mukorsi dam is still under construction, it would take about seven years for the ethanol project – a sugar cane irrigation scheme – to be established, during which time, flood victims say, they will have no source of livelihood. Bhasikiti said the families were resettled there to enable them to benefit from the sugar cane irrigation scheme when it eventually becomes operational.	Human Rights Watch (2015)

Inadequate or no compensation measures	The result was that those displaced lost much moveable property for which they have not been compensated. Rather than being compensated fairly for the land they lost and allowed freedom of movement, families were impoverished and offered a stark choice: accept comparably tiny plots and become farmworkers on a sugar cane plantation or lose even the inadequate aid on offer from the government. And even should they take the government's deal, it is unclear whether they could hope to derive a sustainable living, or even steady tenure over this farmland, because of the dispute over the title ownership of the land.	Human Rights Watch (2015)
Inadequate resettlement	It has taken the LHDA years to build replacement houses for displaced people. Many of those displaced by powerline construction in 1990-91, for example, were still without housing in October 1995, according to the World Bank. Others who lost homes to earthquakes caused when the Katse reservoirs were filled were forced to live in temporary storage-shed type housing for months, including over a very harsh winter. Houses in the Mohale resettlement sites already suffer from cracked walls.	Lang et al. (2000)
Inadequate resettlement	The resettled farmers did not have enough land to farm,	Lang et al. (2000)
Inadequate resettlement	Issues of compensation and resettlement related both to the Bujagali Dam and its transmission lines have not been fully resolved	International Rivers (n.d)
Inadequate resettlement	The Bujagali project violated the lenders policies on resettlement and compensation.	NAPE (2014)
Inadequate resettlement	The resettled community have also faced new challenges to their already inadequate land: several households are complaining that a neighbor community is pushing them out of the land they have gotten. The land containing the resettlement previously belonged to the other village, and recently they have been laying claims to land used by the resettled households.	NAPE (2014)
Inadequate resettlement	The resettled community have also faced new challenges to their already inadequate land: several households are complaining that a neighbor community is pushing them out of the land they have gotten. The land containing the resettlement previously belonged to the other village, and recently they have been laying claims to land used by the resettled households. The	NAPE (2014)
Inadequate resettlement	In Pacux the housing situation has worsened	Colajacomo (1999)
Inadequate resettlement	Compensatory efforts were few and grossly inadequate to meet the basic needs of displaced communities, let alone provide redress for the full extent of lost land, property, communal resources, livelihoods and lives.	Johnston (2010)

Inadequate resettlement	us habitaciones no han sido reconstruidas, viven sin acceso a la energía eléctrica y en su mayoría, sin agua potable, sin asistencia sanitaria, sin vías, en habitaciones autoconstruidas y obligadas a cultivar tierras de bajísimo rendimiento, aproximadamente 1/5 de la productividad normal, las únicas que quedaron disponibles después de la creación del embalse.	Gerebizza (2009)
Inadequate resettlement	– sfollamento forzato di 44.ooo persone, che non hanno ricevuto l'indennizzo previsto dal progetto. Gli sfollati sono stati costretti a spostarsi in villaggi costruiti dalla stessa Impregilo s.p.a., senza accesso all'acqua ed alle infrastrutture di base;	CDCA (n.d.)
Inadequate resettlement	55,000 people were reported to be affected, 15,000 of whom were being accommodated in shelters while 26,300 were in shelters in Caia District.	Scudder (2005)
Inadequate resettlement	By the end of March, flooding was said to have affected 635,000 people, 180,000 of whom were living in emergency shelters	Scudder (2005)
Inadequate resettlement	For those who lost houses, replacement housing took years to complete.	International Rivers (2005)
Inadequate resettlement	Resettled individuals who would otherwise earn income from employment abroad are faced with the dilemma of leaving their families in an unfinished home to earn money for construction or staying while spending their savings.	Human Rights Watch (2014)
Inadequate resettlement	The government does not have an on-the-ground monitoring or outreach system to assess resettled people's needs, to determine whether officials and agencies are fulfilling their duties, or to provide assistance to people in crisis.	Human Rights Watch (2014)
Inadequate resettlement	The government has allocated land to displaced families, but it has not built houses for them on that land nor provided sufficient compensation to all families in accordance with international human rights standards to build new homes of a similar size and standard to those they previously owned.	Human Rights Watch (2014)
Inadequate resettlement	The government has allocated land to displaced families, but it has not built houses for them on that land nor provided sufficient compensation to all families in accordance with international human rights standards to build new homes of a similar size and standard to those they previously owned.	Human Rights Watch (2014)
Inadequate resettlement	Those who built their own houses told Human Rights Watch researchers that they spent on average three years building the main house, plus additional time constructing outbuildings such as a kitchen, bathing room, food storage shed, and toilet, consistent with their previous living structures and typical households in Tajikistan.<br>Longer construction times often compelled families who resettled quickly to new villages to live in half-completed houses, sometimes cooking outdoors or bathing in unfinished basements. In other cases, families remained in their original homes while male relatives	Human Rights Watch (2014)

	regularly traveled distances of up to 200 kilometers to complete construction of new homes, often spending significant amounts of money on transportation. Men reported spending as much as a month at a time away from their families, sleeping in tents, neighbors' homes, or in their unfinished houses while building their new homes. Many families in this situation noted the significant costs of running two households at once.	
Inadequate resettlement	Part of the land in Chingwizi, in Mavingo, has been temporarily allocated to victims of the TokweMurkosi floods of the just-ended rainy season, but DTZ says politicians are ominously encroaching deeper into Nuanetsi Ranch which is owned by DTZ.<br>The politicians are also allegedly planning to parcel out part of the land to Triangle Sugar Limited, with whom they aim to have a joint venture, without the blessings of DTZ, where controversial tycoon Billy Rautenbach has a massive crocodile farming project and wants to establish an ethanol plant.	Mambo (2014)
Inadequate resettlement	The Masvingo Zanu PF leadership is however said to have engaged Triangle Sugar Limited to form a joint venture company to grow cane on the land meant for the displaced. "The people are just being given half a hectare of dry land plots to pacify them. We are concerned that lack of consultation with DTZ could be evidence that the current developments constitute land invasions disguised as land compensation for people from Tokwe Murkosi," said a senior DTZ official.	Mambo (2014)
Inadequate resettlement	Four resettled women told Human Rights Watch that they continue to live in tents and the government has directed all resettled people not to build any permanent structures on the plots they were allocated	Human Rights Watch (2015)
Inadequate resettlement	The government shut down the camp in August in an attempt to permanently relocate the families on a different part of Nuanetsi Ranch where each family was allocated a one-hectare plot of land. The families would now have significantly less land than they previously owned when they were in Masvingo.	Human Rights Watch (2015)
Inadequate resettlement	Today, a year after the disaster, these 3,300 families are completely dependent on aid for food and shelter on Nuanetsi Ranch, and are unable to build permanent homes as ownership of the land is in dispute.	Human Rights Watch (2015)
Health related issues	The reservoir will form an extensive, shallow body of water with relatively little circulation. Large volumes of organic mater and minerals are expected to accumulate in the impounded water, conditions which create an ideal breeding ground for carriers of tropical diseases such as mosquitoes.	Lang et al. (2000)

Health related issues	The resettled farmers did not have enough land to farm, and in 1966 the World Food Programme and USAID sent over 6,000 tonnes of emergency food aid in an attempt to prevent the resettled villagers from starving.	Lang et al. (2000)
Health related issues	Water-borne diseases such as schistosomiasis, ochocerciasis and malaria have increased dramatically since the filling of the reservoir.	Lang et al. (2000)
Health related issues	drug addiction had become so widespread among young men, especially in Khallabat.	Bennet and McDowell (2012)
Health related issues	Everyone is uneasy. One or two of every family are mentally upset.”	Bennet and McDowell (2012)
Health related issues	Life is full of worries . . . joblessness has taken away peace of mind.	Bennet and McDowell (2012)
Health related issues	One can [look around] here and see how unemployed young people are taking drugs; most of them are using heroin; some are using chars [hashish]. We have no words to describe their plight. Pay a visit to this place late at night and you will find them in groups of five or six, busy using drugs of various kinds.	Bennet and McDowell (2012)
Health related issues	perennial hunger	Bennet and McDowell (2012)
Health related issues	Several narrators commented that, soon after relocation, a significant number of older people died prematurely. The causes of these deaths cannot be known but the narrators made a direct link between the experience of relocation and the trauma of losing their land and home.	Bennet and McDowell (2012)
Health related issues	La fumigación con esos venenos ha generado problemas de salud en la gente, ya hay antecedentes de intoxicaciones y eso ese uno los impactos que nos trajo el embalse y que nadie se detiene a analizar"	SEMANA (2019)
Health related issues	lo cual causó de inmediato graves problemas, especialmente económicos, sociales y psicológicos	SEMANA (2019)
Health related issues	All medicinal plants growing along the river were lost due to the dam construction and the two Maya priests of the community as well as those who knew traditional healing systems were killed.	Colajacomo (1999)
Health related issues	Relocation has caused worsened living conditions for local communities and the quality of their nutrition.	Colajacomo (1999)
Health related issues	The health centre built by INDE is unfit for use: soon after its construction, it lacked medical staff and medicines.	Colajacomo (1999)

Health related issues	Thirty years ago a massacre took place in the Guatemalan highlands that left 400 people dead. Countless more were displaced, tortured, raped or left starving. And all to make way for a hydroelectric dam.	Dearden (2012)
Health related issues	degenerative effect on the culture, economy and health of both displaced and still resident communities	Johnston (2010)
Health related issues	significant declines in dietary protein, declines that help explain the region's extraordinarily high rates of malnutrition and infant mortality.	Johnston (2010)
Health related issues	Ten years of life under such guarded conditions produced an array of social, economic and psychological damages.	Johnston (2010)
Health related issues	Las mismas plantas además, son también vectores para la transmisión de enfermedades infecciosas: zancudos y moscas infectadas depositan sus huevos en el interior de las plantas acuáticas, aumentando en el curso de los años, la incidencia de enfermedades infectivas como la malaria y el dengue, incluido el dengue hemorrágico, presente en la comunidad en porcentajes relativamente altos. De las entrevistas efectuadas emergieron preocupantes índices de mortalidad infantil y de ancianos, debido sobretodo, a hepatitis generadas por consumo de agua no potable y por enfermedades infecciosas difundidas a través de los zancudos.	Gerebizza (2009)
Health related issues	Cette retenue d'eau créée par le barrage a favorisé la prolifération de plusieurs vecteurs de maladies hydriques. Ces pathologies qui affectent la santé et la qualité de vie des populations sont le paludisme, le schistosomiase et l'onchocercose.	Kourouma and Lamah (n.d.)
Health related issues	The reservoir created by the dam has already increased the incidence of diseases in flooded areas upstream	Franchi and Manes (2016)
Health related issues	La contaminación del río no solo les ha causado enfermedades cutáneas y respiratorias,	CENSAT-AguaViva (n.d.)
Health related issues	an epidemic of human sleeping sickness broke out that killed an unknown number of people.	Scudder (2005)
Health related issues	Gwembe Tonga anxiety about what the future might bring, though ill-defined, was prescient. It	Scudder (2005)
Health related issues	health problems, resentment and loss of customs anticipated the physiological, psychological and cultural components of multidimensional stress which became a major threat to Gwembe Tonga society	Scudder (2005)
Health related issues	In explaining why he said "When we stayed by the river, the water was fast-flowing and safe to drink. Once we were moved, we had to drink stagnant water and I think this is what killed my children"	Scudder (2005)
Health related issues	increase in the prevalence of malnutrition, diarrhea and malaria	Scudder (2005)

Health related issues	Throughout the Valley the first two years following removal were the most difficult for the Gwembe Tonga (Scudder and Colson, 1982: 142, Colson, 1971). Both “grieving for a lost home” (Fried, 1963) and anxiety for the future characterized Gwembe Tonga resettlers. In the Lusitu, reactions to the mystery deaths are an extreme example of the impact of resettlement on mental health.	Scudder (2005)
Health related issues	d'autres régions du lac o ment a provoqué des crises psychologiques	PROWIZUR (1976)
Health related issues	Il a été noté par endroit l’existence de nombreuses maladies endémiques (paludisme, diarrhée, maladies respiratoires).	Pittalunga (2002)
Health related issues	The respondents explained that the post-Kpong Dam construction has not only led to a high prevalence of existing waterborne diseases, but also contributed to the emergence of new ones such as cholera.	Owusu et al (2016)
Health related issues	This may have resulted in psychological stresses among parents and household heads who are responsible for sustaining the social and economic situation of their family or household members.	Owusu et al (2016)
Health related issues	Los problemas sociales aquejan a<br>cientos de familias de las comunas descritas, donde además de pagar por los costos ambientales producto de estos megaproyectos, deben lidiar con un panorama económico desfavorable. Sólo en Alto Biobío el desempleo alcanza el 90 por ciento, mientras que la tasa de suicidios triplica la media nacional.	ElDesconcierto (2014)
Health related issues	AIDS was introduced by the work	International Rivers (2005)
Health related issues	prostitution and alcoholism greatly increased	International Rivers (2005)
Health related issues	Now, because he lacks the space for crops or livestock, he struggles to feed his family.	Human Rights Watch (2014)
Health related issues	They reported having less meat to eat and less milk for their children and families in general, and having a reduced number of hot meals per day.	Human Rights Watch (2014)
Health related issues	Through this mindless vandalism against nature, the Icelandic tourist industry will also be affected and the health and life of the Icelandic people.	Saving Iceland (2005)
Health related issues	Some measures, such as malaria protection for the population living in the highlands, were introduced too late and were not pursued vigorously enough. If accompanying low-cost health measures had been introduced in time (education, mosquito nets, drugs for treating persons who have contracted the disease, etc.) the spread of malaria in the highlands could at least have been considerably slowed.	KFW (2006)

Health related issues	were exposed to malaria, but had no access to adequate supplies of anti-malarial drugs	Human Rights Watch (2015)
Increase in violence and crime	aumento de la criminalidad, aumento de la demandad de instalaciones penitenciarias,	Galindo Vanegas (2018)
Increase in violence and crime	Before independence, people were living in harmony. But now that the lake has reduced, the other tribes have moved closer and raids have intensified along with killings on both sides.”	Allibhai (2015)
Increase in violence and crime	intensification of thefts among various communities.	Franchi and Manes (2016)
Increase in violence and crime	prostitution and alcoholism greatly increased	International Rivers (2005)
Local poverty creation		
Damage or loss of properties and households	A number of households from both sides of the river banks in the districts of Buyikwe (western bank) and the district of Jinja (Eastern bank) in 2009 raised concerns about damages to their houses due to blasting of rocks at the dam site. People’s houses developed cracks as a result of the blasting. In the same year, Salini Constructori –the company contracted by BEL to construct the dam denied responsibility, arguing that houses affected were outside the area specified for the impact. They also said that those buildings affected were not built according to civil engineering standards.	NAPE (2014)
Damage or loss of properties and households	In Pacux the housing situation has worsened	Colajacomo (1999)
Damage or loss of properties and households	In 2004, people in the resettlement villages were living in extreme poverty, with homes crumbling	Johnston (2010)
Damage or loss of properties and households	us habitaciones no han sido reconstruidas, viven sin acceso a la energía eléctrica y en su mayoría, sin agua potable, sin asistencia sanitaria, sin vías, en habitaciones autoconstruidas y obligadas a cultivar tierras de bajísimo rendimiento, aproximadamente 1/5 de la productividad normal, las únicas que quedaron disponibles después de la creación del embalse.	Gerebizza (2009)
Damage or loss of properties and households	Three months ago, the government bulldozers destroyed my workshop. I went to the district administration demanding compensation and I presented my appeal to the project Omo-Kuraz Sugar coordination office in Hanna. No one has answered	Franchi and Manes (2016)

Damage or loss of properties and households	En la cuenca media, las explosiones han causado réplicas telúricas y varias viviendas presentan facturas.	Roa Avendaño and Duarte Abadía (2012)
Damage or loss of properties and households	After three flood gates were opened on February 26th, downstream Zambezi waters had risen the next day by “at least” five meters in the Lusitu area and below Chirundu “with the flood waters sometimes reaching 1.5 kms inland.” In Chiawa approximately two-thirds of the households of a population of about 8,000 had their riverbank gardens flooded.	Scudder (2005)
Damage or loss of properties and households	After three flood gates were opened on February 26th, downstream Zambezi waters had risen the next day by “at least” five meters in the Lusitu area and below Chirundu “with the flood waters sometimes reaching 1.5 kms inland.” In Chiawa approximately two-thirds of the households of a population of about 8,000 had their riverbank gardens flooded.	Scudder (2005)
Damage or loss of properties and households	Les populations déplacées n’ayant pas pour la plupart de terre pour pratiquer l’agriculture	Raphaël et al (2019)
Damage or loss of properties and households	More than 60% of the respondents reported that the construction of the Kpong Dam has led to the loss of property in the downstream communities. The respondents recalled that many of their foodstuffs, farmlands, houses and other valuable properties were inundated during the “great floods” in the 1960s and 1980s when the Akosombo and Kpong Dams were opened, respectively.	Owusu et al (2016)
Damage or loss of properties and households	as a result of frequent blasting and earth moving activity, many residents have suffered damage to their homes	Human Rights Watch (2014)
Damage or loss of properties and households	People still living in these communities stated that blasting for these materials has damaged their homes, shattering all of their windows and cracking their walls.	Human Rights Watch (2014)
Damage or loss of properties and households	It has taken the LHDA years to build replacement houses for displaced people. Many of those displaced by powerline construction in 1990-91, for example, were still without housing in October 1995, according to the World Bank. Others who lost homes to earthquakes caused when the Katse reservoirs were filled were forced to live in temporary storage-shed type housing for months, including over a very harsh winter. Houses in the Mohale resettlement sites already suffer from cracked walls.	Lang et al. (2000)
Damage or loss of properties and households	Many indigenous Indian peoples depend on the streams and rivers that connect with the Caroni river for fishing and drinking water. The project will disrupt local settlements, affecting	Lang et al. (2000)

	infrastructure and services. At least 1,014 people will be displaced by the project, losing homes and farmland.	
Damage or loss of properties and households	Those who built their own houses told Human Rights Watch researchers that they spent on average three years building the main house, plus additional time constructing outbuildings such as a kitchen, bathing room, food storage shed, and toilet, consistent with their previous living structures and typical households in Tajikistan.<br>Longer construction times often compelled families who resettled quickly to new villages to live in half-completed houses, sometimes cooking outdoors or bathing in unfinished basements. In other cases, families remained in their original homes while male relatives regularly traveled distances of up to 200 kilometers to complete construction of new homes, often spending significant amounts of money on transportation. Men reported spending as much as a month at a time away from their families, sleeping in tents, neighbors' homes, or in their unfinished houses while building their new homes. Many families in this situation noted the significant costs of running two households at once.	Human Rights Watch (2014)
Damage or loss of properties and households	much household property was lost during the forced removal	Human Rights Watch (2015)
Damage or loss of properties and households	Today, a year after the disaster, these 3,300 families are completely dependent on aid for food and shelter on Nuanetsi Ranch, and are unable to build permanent homes as ownership of the land is in dispute.	Human Rights Watch (2015)
Energy Poverty	Eight out of 10 families in the Paraguayan capital, Asuncion, have electricity, but four out of five families living in rural Caazapa do not.	Lang et al. (2000)
Energy Poverty	in 1994 the reservoir level fell below the new operating minimum, resulting in electricity blackouts of up to 20 hours a week	Lang et al. (2000)
Energy Poverty	Bujugali Dam failed to help the 86% of Uganda's population who are not connected to the national grid.	International Rivers (n.d)
Energy Poverty	The dam-affected people are concerned of the slow progress of connecting electricity to the resettlement area. Uganda Electricity Transmission Company Limited (UECTL) installed the utility poles and lines in 2012, however the power has not been turned on to-date. The affected people are expected to pay a connection fee of UGX 250.000 (USD 100) which they say they cannot afford. Only 11 households have agreed to pay for connection. The community expected to be provided electricity for free as was promised by AES initially, but this is not likely to be the case. BEL has however, committed to pay for connection in the surrounding community including the 11 households that had agreed. It is not clear when this will happen.	NAPE (2014)

Energy Poverty	very few will actually receive energy access. Increased access to electricity will only benefit those already connected to the electrical grid. Approximately 85% of Ethiopians live in rural areas, only 2% of whom have access to electricity, while 86% of the urban dwellers are able to enjoy this privilege (Energypedia, 2016). Whether dividends will materialize is also an open question; the odds of the GERD generating above 2800 MW are low and there may be no dividends to share.	International Rivers (2017)
Energy Poverty	Mais très peu de ménages de ces localités sont raccordés au réseau électrique. Seulement 6,8% de ménages possèdent l'électricité à N'denou et 15,4% à Diéviessou.	Pittalunga (2002)
Energy Poverty	During the winter months, the government provides electricity to families in resettled communities on a schedule, usually for three to four hours in the morning and three to four hours in the evening,	Human Rights Watch (2014)
Energy Poverty	In addition, outside of the summer months, the government provides electricity to resettled communities for only a few hours per day.	Human Rights Watch (2014)
Energy Poverty	Only a handful of people interviewed by Human Rights Watch reported having continuous access to electricity either before or after resettlement.	Human Rights Watch (2014)
Loss of basic facilities and services	A schoolchild whose family was uprooted by the Katse, when asked to comment on the dam, wrote: • There is nothing worse than working hard at something and then have something come and destroy it. We were satisfied with the way we were working. We were plowing maize and beans. We were eating fresh maize. We had trees. We had firewood and people were buying it from us. We were getting money and we were able to go to school. When LHDA came and destroyed everything that was important to my family, we started to become poor. The dam took our fields and our trees. That was the end of our money. We needed to look hard to find enough money for us to attend school. We were given maize, beans, and a little money, but it is not as much as we were producing before. That was the end of our fire and fresh maize. Now, when I look at the dam, I still get very angry.•	Lang et al. (2000)
Loss of basic facilities and services	Antes de que el gobierno nacional tomara la decisión de inundar las 1.260 hectáreas que conforman el embalse, entre lo que hoy son los puertos de Batá I y Batá II, existía un puente sobre el río Batá que permitía a las comunidades pasar a cualquier hora con sus mercancías y sus animales.<br>Tras el llenado de la represa, en 1975, retiraron el puente. Las comunidades pidieron construirlo de nuevo. Conformaron una comisión negociadora que se encargaría de hacer valer lo estipulado en el plan de obra y en el presupuesto general que contemplaba esa estructura. El Estado, por medio de Interconexión Eléctrica (ISA), empresa que ejecutó el proyecto, se	SEMANA (2019)

	comprometió a estudiar la respuesta.<br>44 años han pasado desde entonces y no hay asomo del puente.	
Loss of basic facilities and services	enfermarse está prácticamente prohibido en ese sector durante el verano. Sin transporte fluvial, trasladar a un paciente hasta el centro de salud del municipio se convierte en una odisea difícil y muy costosa. Deben pagar hasta 30.000 pesos por un expreso en carro particular.	SEMANA (2019)
Loss of basic facilities and services	el índice de carencias de servicios de base entre la comunidad ubicada al interior del embalse, está entre los más altos del País.	Gerebizza (2009)
Loss of basic facilities and services	Faible niveau d'éducation et de qualification	Pittalunga (2002)
Loss of basic facilities and services	Le mauvais état des routes affecte l'écoulement des produits agricoles et aggravent les problèmes de commercialisation. De façon générale, il n'existe pas de routes reliant le lac aux d riifférents villages verains. Il n'existe que des sentiers encore mal entretenus. L'accès aux campements de pêche situés au bord du lac reste une contrainte majeure.	Pittalunga (2002)
Loss of basic facilities and services	The government is responsible for building schools in each resettled community, but despite Flood Zone Directorate representatives emphasizing plans to prioritize building schools, the communities in Rudaki and Dangara have been without a local school for several years. Children and parents in those areas reported that children spent as much as two hours walking to school, due to the long distances. During certain times of the year, children as young as seven years old must walk to or from school in the dark. Some parents told Human Rights Watch that they keep their children home from school in the cold or inclement weather and reported that their children resisted attending school because of the long walk. Parents in both resettled and yet to be resettled communities reported that children with disabilities did not attend local schools, and only a few went to specialized education programs in Dushanbe for a few weeks per year.	Human Rights Watch (2014)
Loss of basic facilities and services	A school for the displaced, called Mulale, which was relocated from Chingwizi to Nyoni section, charges $55 per term school fees. The sum is beyond the reach of most parents or guardians affected by the floods, who are solely dependent on aid. As a result, hundreds of children are at risk of being unable to continue their education as there are no other schools for the displaced.	Human Rights Watch (2015)
Loss of livelihoods and employment	A schoolchild whose family was uprooted by the Katse, when asked to comment on the dam, wrote: • There is nothing worse than working hard at something and then have something come and destroy it. We were satisfied with the way we were working. We were plowing maize and beans. We were eating fresh maize. We had trees. We had firewood and people	Lang et al. (2000)

	were buying it from us. We were getting money and we were able to go to school. When LHDA came and destroyed everything that was important to my family, we started to become poor. The dam took our fields and our trees. That was the end of our money. We needed to look hard to find enough money for us to attend school. We were given maize, beans, and a little money, but it is not as much as we were producing before. That was the end of our fire and fresh maize. Now, when I look at the dam, I still get very angry.•	
Loss of livelihoods and employment	About 17,000 farmers lost their land to make way for the dam and agribusinesses. Years later, many were still landless and jobless.	Lang et al. (2000)
Loss of livelihoods and employment	Hundreds of thousands more people were adversely affected as their grazing and agricultural lands are no longer irrigated by the river's annual flood34	Lang et al. (2000)
Loss of livelihoods and employment	Loss of Livelihoods Of the total land area of Lesotho, less than 10% is suitable for arable farming. The Mohale valley, which would be flooded when the Mohale dam is completed, contains Lesotho's most fertile land and is the only region in the country to produce a surplus. Phases 1A and 1B of the project will together result in the loss of 4,635 hectares of grazing land and 1,500 hectares of arable land, according to the World Bank.	Lang et al. (2000)
Loss of livelihoods and employment	Many indigenous Indian peoples depend on the streams and rivers that connect with the Caroni river for fishing and drinking water. The project will disrupt local settlements, affecting infrastructure and services. At least 1,014 people will be displaced by the project, losing homes and farmland.	Lang et al. (2000)
Loss of livelihoods and employment	Measures taken to help the 24,000 people who lost their farms, homes or access to communal grazing land as a result of Phase 1 of the LHWP have been heavily criticised as ineffective. Because Lesotho has so little arable land, those evicted to make way for the reservoirs have not been given replacement farmland but are forced to find new livelihoods. The Mohale will affect another 7,400 people.	Lang et al. (2000)
Loss of livelihoods and employment	The panel also found that thousands of people have lost their jobs as a result of the dam	Lang et al. (2000)
Loss of livelihoods and employment	the water supply is neither reliable nor safe.	Lang et al. (2000)
Loss of livelihoods and employment	Yam production in the floodplain area fell by 100,000 tonnes after the dam's completion, while downstream fish stocks fell by 60-70%. •	Lang et al. (2000)
Loss of livelihoods and employment	Los efectos negativos que han causado estas ocupaciones en el territorio son cuantiosos. Desapareció parte de una cultura milenaria, desapareció un ecosistema prístino de proporciones, y junto con esto también un polo de desarrollo que convivía en forma armónica con la cultura mapuche pewenche y su entorno: el ecoturismo.	OLCA (2014)

Loss of livelihoods and employment	Los efectos negativos que han causado estas ocupaciones en el territorio son cuantiosos. Desapareció parte de una cultura milenaria, desapareció un ecosistema prístino de proporciones, y junto con esto también un polo de desarrollo que convivía en forma armónica con la cultura mapuche pewenche y su entorno: el ecoturismo.	OLCA (2014)
Loss of livelihoods and employment	in the absence of proper employment many men could only get intermittent casual labor.	Bennet and McDowell (2012)
Loss of livelihoods and employment	Now they have lost their lands	Bennet and McDowell (2012)
Loss of livelihoods and employment	there was unemployment; they had lost everything.	Bennet and McDowell (2012)
Loss of livelihoods and employment	La tragedia para él y otros 200 pescadores artesanales comenzó hace tres semanas, cuando todo lo que podían atrapar con sus redes eran peces muertos.	Eliécier Quintero (2007)
Loss of livelihoods and employment	BEL extended piped water in the resettlement area; however, only 3 water points were constructed in the compounds of 3 houses that now have to sell the water to the rest of the community. These water points are not enough to serve a community of 35 households. The community is concerned about availability as and when water is required as someone has to be present at the water point to grant access. The people are also concerned that this water is not free as there is a fee of UGX 100 for a 20 liter jerry can for utility maintenance, and on average they need 5 jerry cans a day for their domestic use which totals to UGX 500 or 2 US dollars. By Ugandan standards, this cost is very high, as poor people in Uganda live below 1 dollar a day. Although there are also two boreholes where the community can get water without charge, the available water is not enough to serve the whole community of 35 households.	NAPE (2014)
Loss of livelihoods and employment	he substantial negative impact caused by this project (with the loss of traditional economies, lands and housing) makes it now almost impossible for the affected communities to satisfy their primary	Colajacomo (1999)
Loss of livelihoods and employment	Now local people cannot manage or have direct access to natural resources, thus affecting their living conditions and the wider environment.	Colajacomo (1999)
Loss of livelihoods and employment	Compensatory efforts were few and grossly inadequate to meet the basic needs of displaced communities, let alone provide redress for the full extent of lost land, property, communal resources, livelihoods and lives.	Johnston (2010)
Loss of livelihoods and employment	Dam releases occurred with no warning and resulting flash floods destroyed crops, drowned livestock and sometimes killed people.	Johnston (2010)

Loss of livelihoods and employment	Downstream communities experienced water shortages (wells and springs dried up and available water was often contaminated). Crop failures were common.	Johnston (2010)
Loss of livelihoods and employment	For those who remained in the Chixoy river basin, periodic flooding seriously affected the length of the agricultural season and the number of harvests per year. Moreover, many fishing villages saw the complete loss of local fisheries	Johnston (2010)
Loss of livelihoods and employment	In 2004, people in the resettlement villages were living in extreme poverty, with homes crumbling and few economic opportunities	Johnston (2010)
Loss of livelihoods and employment	The declining ability to produce food is related directly to the loss of productive agricultural land, pastureland and access to viable river and forest resources	Johnston (2010)
Loss of livelihoods and employment	The loss of access to fertile lands, pasture and river and forest resources also produced measurable change in household ability to generate monetary income.	Johnston (2010)
Loss of livelihoods and employment	Upstream communities had seen part of their agricultural land flooded, and lost access to land, roads and regional markets	Johnston (2010)
Loss of livelihoods and employment	Después de haber perdido casa, tierra y animales, muchas familias se vieron forzadas a emigrar a otras zonas del País, la mayor parte hacia la ciudad de Guayaquil.	Gerebizza (2009)
Loss of livelihoods and employment	Sus habitaciones no han sido reconstruidas, viven sin acceso a la energía eléctrica y en su mayoría, sin agua potable, sin asistencia sanitaria, sin vías, en habitaciones autoconstruidas y obligadas a cultivar tierras de bajísimo rendimiento, aproximadamente 1/5 de la productividad normal, las únicas que quedaron disponibles después de la creación del embalse.	Gerebizza (2009)
Loss of livelihoods and employment	us habitaciones no han sido reconstruidas, viven sin acceso a la energía eléctrica y en su mayoría, sin agua potable, sin asistencia sanitaria, sin vías, en habitaciones autoconstruidas y obligadas a cultivar tierras de bajísimo rendimiento, aproximadamente 1/5 de la productividad normal, las únicas que quedaron disponibles después de la creación del embalse.	Gerebizza (2009)
Loss of livelihoods and employment	– assenza di un'adeguata manutenzione della diga che ne ha comportato il cedimento, causando la distruzione di 60 villaggi, oltre che la perdita di 100.000 ettari di piantagioni di riso, miglio, frumento e zucchero e di allevamenti; inoltre il mal funzionamento delle turbine ha contaminato l'acqua con un'altissima percentuale di metalli, danneggiando gravemente la popolazione locale dedita alla pesca. Imp	CDCA (n.d.)
Loss of livelihoods and employment	Destruyó el 98 por ciento de la pesca artesanal , que afectó a 3500 familias ,	Interview
Loss of livelihoods and employment	Activities such as flood-recession farming, gold-panning, and fishing will be greatly affected	International Rivers (2017)
Loss of livelihoods and employment	Communities face hunger	The Oakland Institute (2019)

Loss of livelihoods and employment	The end of the Omo River's annual flood has decimated local livelihoods.	The Oakland Institute (2019)
Loss of livelihoods and employment	is already causing the inhabitants a substantial deterioration of food security.	Franchi and Manes (2016)
Loss of livelihoods and employment	dam will cause food insecurity, chronic hunger	International Rivers (2009)
Loss of livelihoods and employment	Secondo gli esperti la riduzione del flusso del fiume causerà l'abbassamento del livello del lago Turkana di circa due terzi. Questo distruggerà la riserve ittiche da cui dipendono centinaia di migliaia di indigeni.	Survival International (2019)
Loss of livelihoods and employment	On behalf of the tribal peoples of the Lower Omo in Ethiopia and of Lake Turkana in Kenya Survival International filed a complaint against Salini Impregilo with the Italian NCP in March 2016. The complaint addresses the human rights violations associated with Salinis construction of the controversial Gibe III dam in the Omo River, which runs from the North of Ethiopia to Lake Turkana that borders Kenya.<br>As the dam has cut off the Omo rivers regular flooding, and made possible the development of irrigated plantation agriculture in the Lower Omo Valley, the complainant alleges that the dam is set to destroy the livelihoods of hundreds of thousands of tribal people in Ethiopia and Kenya who live along the river and around the shores of the lake	OECD Watch (2017)
Loss of livelihoods and employment	Afectación a la soberanía alimentaria:	Moreno Socha (2019)
Loss of livelihoods and employment	Las personas que extraían piedra del río perdieron este medio de vida.	Roa Avendaño and Duarte Abadía (2012)
Loss of livelihoods and employment	Estas transformaciones ambientales inciden negativamente en los medios de vida de las poblaciones que dependen directamente de las dinámicas del río: la pesca, la minería artesanal y la agricultura de subsistencia. De un lado, su soberanía alimentaria se ve amenazada por la alta e incontrolada a mortandad de peces que causa la desviación del río y el represamiento de las aguas. Además, la destrucción de las unidades secas de los ríos (playones y vegas) inhibe la siembra de cultivos de subsistencia. De otro lado, pescadores, mujeres vendedoras de pescado y campesinos han sido desplazados por la obra y sus actividades son prohibidas o han sido restringidas. De esta manera, estos pobladores han perdido su seguridad y autonomía alimentaria y en la actualidad sus medios de vida dependen cada vez más del mercado externo	Roa Avendaño and Duarte Abadía (2013)

Loss of livelihoods and employment	Los impactos ambientales que genera la represa, en su primera fase de construcción, están afectando directamente los medios de vida de las poblaciones que viven del río.	Roa Avendaño and Duarte Abadía (2013)
Loss of livelihoods and employment	In February 2000 and February 2001, flood releases from Kariba devastated communities from below the Kariba Gorge to the Mozambique border as well as within Mozambique.	Scudder (2005)
Loss of livelihoods and employment	In the short term, people will be extremely short of their usual foods for at least three months	Scudder (2005)
Loss of livelihoods and employment	Inadequately announced to downstream residents, releases in late February 2000 destroyed much of the maize crop in Chief Chiawa's area just before its harvest. According to a BBC report March 8, "More than 12,000 people are at risk of starvation… Crops of maize, bananas, pumpkins and groundnuts were submerged by the rising flood waters. Local people have been so desperate to salvage any of the precious food that they have been diving from canoes to try to harvest the underwater crops…at least one farmer has been attacked and seriously injured by a crocodile…Local people complain that they were given very little information…and there certainly seems to be no contingency plan in place to help people" (BBC, March 6, 2000)	Scudder (2005)
Loss of livelihoods and employment	More than 12,000 people are at risk of starvation… Crops of maize, bananas, pumpkins and groundnuts were submerged by the rising flood waters. Loc	Scudder (2005)
Loss of livelihoods and employment	opening of the spillgates at Kariba dam has had a devastating impact on the livelihood of people living downstream…	Scudder (2005)
Loss of livelihoods and employment	The actual cause of the mystery deaths remains unknown. Based on the symptoms, the clustering of deaths toward the end of the dry season, and the age and sex of the victims, the most likely cause was consumption of toxic plants gathered by women and children during periods of food scarcity.	Scudder (2005)
Loss of livelihoods and employment	the productivity of the Sofala Banks shrimp fishery, which is one of Mozambique's major sources of foreign exchange, has been adversely affected with reduced yields correlated with dam-induced reduced runoff	Scudder (2005)
Loss of livelihoods and employment	Celles qui trouvent assez éloignés les anciens villages, continuent de quémander directement auprès du village d'accueil les compléments de terre nécessaires à leur subsistance	Raphaël et al (2019)
Loss of livelihoods and employment	Les populations déplacées n'ayant pas pour la plupart de terre pour pratiquer l'agriculture	Raphaël et al (2019)
Loss of livelihoods and employment	'absence des terres cultivables et les difficultés d'accès à la terre est relevée comme une autre cause importante de la pauvreté, surtout dans certains villages déplacés (AVB). Pour certaines populations déplacées les terres cultivables restent insuffisantes	Pittalunga (2002)

Loss of livelihoods and employment	Some respondents (22%) complained that the practice of opening spillways often resulted in the destruction of farms and fish cages below the dam.	Owusu et al (2016)
Loss of livelihoods and employment	the construction of the dam has led to the collapse of other economic activities that some of the people in the community depended on for their sustenance. A well-known activity reported was the canoe transport operation along the Lower Volta River. The majority of the respondents (70%) attributed this collapse to low downstream water level after the impoundment of the dam.	Owusu et al (2016)
Loss of livelihoods and employment	The most serious effect of the Kpong Dam reported by the respondents in both Amedika (90%) and Natriku (70%) was the loss of recession agriculture.	Owusu et al (2016)
Loss of livelihoods and employment	Los problemas sociales aquejan a cientos de familias de las comunas descritas, donde además de pagar por los costos ambientales producto de estos megaproyectos, deben lidiar con un panorama económico desfavorable. Sólo en Alto Biobío el desempleo alcanza el 90 por ciento, mientras que la tasa de suicidios triplica la media nacional.	ElDesconcierto (2014)
Loss of livelihoods and employment	The once remote mountain communities of the Lesotho Highlands have been changed dramatically by the project. Some 20,000 people moved into the area to work on the project, and many squatters' camps were established near the project as well.	International Rivers (2005)
Loss of livelihoods and employment	Though not required to shift their residence, some were adversely affected because they lost arable land and/or grazing to the project, or because their livelihood below project dams could be adversely affected by fundamental changes in a river's natural annual regime.	Lenka Thamae and Pottinger (2006)
Loss of livelihoods and employment	Las principales actividades económicas del establecimiento, antes de producirse el traslado forzoso hacia el actual emplazamiento ubicado en el paraje Corralito, eran la agricultura y la ganadería, especializándose en la producción ovina. De la superficie total de 7.500 has más de 500 se encontraban bajo riego. Cabe destacar que estas últimas, así como las mejoras más importantes de la explotación, se encontraban dentro de las 2400 has inundadas.	Balazote and Radovich (2003)
Loss of livelihoods and employment	Government representatives have acknowledged the importance of farmland to people facing resettlement. The Flood Zone Directorate has allocated 100 and 80 hectares of land for farms and pastures in two of the resettlement sites, Saidon y Bolo and Yoli Garm Oba, respectively, close to existing villages near the dam site that were under construction when Human Rights Watch visited in early 2014. However, those resettled to some other sites will not have access to farm and pasture land.	Human Rights Watch (2014)
Loss of livelihoods and employment	In some resettled communities visited by Human Rights Watch, residents faced water shortages that left them without sufficient water for drinking and other household needs and prevented them from growing even a small amount of food. Prior to resettlement, families typically had continual access to water via mountain springs. In certain resettled communities,	Human Rights Watch (2014)

	they often receive water supplied by electric pumps for only a few hours a day. To compensate for the lack of reliable water supply, resettled families collect water in bottles and tanks to use throughout the day for drinking and household tasks, but when mechanical problems interrupt the water supply, they must walk several kilometers to a canal or river and carry containers of water home. Resettled residents also stated that water shortages made it difficult to mix concrete or make clay bricks used to build their homes, slowing construction times.	
Loss of livelihoods and employment	Now, because he lacks the space for crops or livestock, he struggles to feed his family.	Human Rights Watch (2014)
Loss of livelihoods and employment	Resettled people also reported few prospects for formal, long term employment. Resettled people in some communities complained that, despite government promises of job placement and assistance, they have not been able to secure employment.	Human Rights Watch (2014)
Loss of livelihoods and employment	We now don't have enough food. We have a hot meal only once per day, for lunch. We have meat just one time per month. Every week we have 30 eggs for 10 people. In Sicharog we had hot milk for breakfast, hot meals for lunch and dinner. We had meat every day there. The old place was better. We used to be able to feed ourselves from the land and from our animals. Here we must buy everything and there is not enough money.	Human Rights Watch (2014)
Loss of livelihoods and employment	we only have 800 square meters each, so we can't grow enough food for the family.	Human Rights Watch (2014)
Loss of livelihoods and employment	Through this mindless vandalism against nature, the Icelandic tourist industry will also be affected and the health and life of the Icelandic people.	Saving Iceland (2005)
Loss of livelihoods and employment	The flood victims contend that until the irrigation scheme becomes fully operational, they will not have a sustainable source of livelihood.	Human Rights Watch (2015)
Loss of livelihoods and employment	their crops were destroyed by the floods	Human Rights Watch (2015)
Loss of livelihoods and employment	Today, a year after the disaster, these 3,300 families are completely dependent on aid for food and shelter on Nuanetsi Ranch, and are unable to build permanent homes as ownership of the land is in dispute.	Human Rights Watch (2015)
Malnutrition and lack of safe water	Many indigenous Indian peoples depend on the streams and rivers that connect with the Caroni river for fishing and drinking water. The project will disrupt local settlements, affecting infrastructure and services. At least 1,014 people will be displaced by the project, losing homes and farmland.	Lang et al. (2000)
Malnutrition and lack of safe water	the water supply is neither reliable nor safe.	Lang et al. (2000)

Malnutrition and lack of safe water	BEL extended piped water in the resettlement area; however, only 3 water points were constructed in the compounds of 3 houses that now have to sell the water to the rest of the community. These water points are not enough to serve a community of 35 households. The community is concerned about availability as and when water is required as someone has to be present at the water point to grant access. The people are also concerned that this water is not free as there is a fee of UGX 100 for a 20 liter jerry can for utility maintenance, and on average they need 5 jerry cans a day for their domestic use which totals to UGX 500 or 2 US dollars. By Ugandan standards, this cost is very high, as poor people in Uganda live below 1 dollar a day. Although there are also two boreholes where the community can get water without charge, the available water is not enough to serve the whole community of 35 households.	NAPE (2014)
Malnutrition and lack of safe water	Entre el 70 y el 90% de los habitantes no tiene acceso al agua potable.	Gerebizza (2009)
Malnutrition and lack of safe water	inadequate water supplies, and severe protein deficiencies presumably increased the death rate from dysentery in the months immediately after resettlement	Scudder (2005)
Malnutrition and lack of safe water	the farmers in both Amedika and Natriku reported that the cost of farm inputs such as fertilizers has impeded their production.	Owusu et al (2016)
Malnutrition and lack of safe water	most of the boreholes were dry, and that the few that were not dry produced saline water that is unsuitable for drinking. As a result, people must walk up to 20 kilometers in search of potable water.	Human Rights Watch (2015)
Monetary poverty	There were financial hardships	Bennet and McDowell (2012)
Monetary poverty	The loss of access to fertile lands, pasture and river and forest resources also produced measurable change in household ability to generate monetary income.	Johnston (2010)
Monetary poverty	The loss of access to fertile lands, pasture and river and forest resources also produced measurable change in household ability to generate monetary income.	Johnston (2010)
Monetary poverty	incremento en el costo de la vida	Roa Avendaño and Duarte Abadía (2012)
Monetary poverty	the farmers in both Amedika and Natriku reported that the cost of farm inputs such as fertilizers has impeded their production.	Owusu et al (2016)
Monetary poverty	The quotation above elucidates how the loss of economic livelihoods induced by the dam may have significantly affected the social and economic standards of families by worsening poverty levels.	Owusu et al (2016)

Monetary poverty	People who had previously relied on their lands to provide food reported that, after resettlement, they had to purchase most or all of their food at markets, leaving less money for other household needs.	Human Rights Watch (2014)
Monetary poverty	Today, a year after the disaster, these 3,300 families are completely dependent on aid for food and shelter on Nuanetsi Ranch, and are unable to build permanent homes as ownership of the land is in dispute.	Human Rights Watch (2015)
Loss or threat to cultural identity	More than 210 villages were flooded or washed away.36	Lang et al. (2000)
Loss or threat to cultural identity	The Volta River Authority is now resettling 80,000 people, from seven different ethnic groups, desirous of preserving their own culture and traditions	Hilton (1966)
Loss or threat to cultural identity	Destruyen lugares de alto valor ambiental, modificando y borrando para siempre los ecosistemas, la memoria y la historia del territorio reemplazándolos por los embalses muertos que además siembran el temor acarrean un peligro por su cercanía a los volcanes. El territorio mapuche pewenche sigue sin protección alguna ante la amenaza del capital privado.	OLCA (2014)
Loss or threat to cultural identity	Los efectos negativos que han causado estas ocupaciones en el territorio son cuantiosos. Desapareció parte de una cultura milenaria, desapareció un ecosistema prístino de proporciones, y junto con esto también un polo de desarrollo que convivía en forma armónica con la cultura mapuche pewenche y su entorno: el ecoturismo.	OLCA (2014)
Loss or threat to cultural identity	A strong concern voiced in the accounts was the declining contact with the extended family, in particular how this had diminished the role of older relatives as guides or mentors.	Bennet and McDowell (2012)
Loss or threat to cultural identity	dismantling of social relations and networks, the loss of identity and status, combined with personal feelings of grief, anxiety, and powerlessness, seem to have undermined the ability of some individuals to recover from displacement.	Bennet and McDowell (2012)
Loss or threat to cultural identity	Every family had its identity. But the dam has scattered us [to different resettlement sites]. Families have disintegrated. And now our children, because of the distance, do not recognize their nearest and dearest. It is the greatest of the losses we have suffered.	Bennet and McDowell (2012)
Loss or threat to cultural identity	In common with involuntary resettlement more generally, communities were not resettled together, so extended families were separated and the old social order and relationships were disrupted.	Bennet and McDowell (2012)
Loss or threat to cultural identity	Some of the higher land inundated was in more remote tribal areas, with particular patterns of land ownership and governance.	Bennet and McDowell (2012)
Loss or threat to cultural identity	We had our own culture and customs. We had a set way of life. All that has been disturbed . . . I still dream of those orchards, streets, and fields. We are living in this township for more than 27 years now but we never dream about this area. We will always remember that place.	Bennet and McDowell (2012)

Loss or threat to cultural identity	The dam drowned the treasured Bujagali Falls – a spectacular series of cascading rapids that Ugandans considered a national treasure. The dam submerged a place with great cultural and spiritual importance of the Basoga people, indigenous dwellers in the dam's project area	International Rivers (n.d)
Loss or threat to cultural identity	Burial sites and protection of indigenous culture and tradition are addressed by a complaint fi led in July 2001	NAPE (2014)
Loss or threat to cultural identity	The Bujagali dam affected the Basoga people's cultural and spiritual institution. The spiritual shrine which was constructed adjacent to the Bujagali Falls has been submerged.	NAPE (2014)
Loss or threat to cultural identity	Por otro lado, AES Chivor presta el servicio de transporte fluvial solo entre las cinco de la mañana y las seis de la tarde. Eso se ha convertido, para muchos de los habitantes de esta población, en un elemento desintegrador que contribuye a la pérdida del tejido social y las costumbres.<br>"Los habitantes de las cinco veredas ubicadas al margen derecho del embalse no pueden quedarse hasta tarde a disfrutar de las fiestas tradicionales o de eventos como el Festival del Agua,	SEMANA (2019)
Loss or threat to cultural identity	As elsewhere in Guatemala, the Rio Negro massacres aimed at depriving the ethnical and cultural groups of their mechanisms of continuity: all elderly people were killed, and they were those who knew and handed down basic religious and cultural practices. Today the survivors find it hard to remember and rebuild their traditions: playing the marimba; dancing the "baile costeño" and the religious syncretism like the candle ritual; furthermore, they haven't got enough money to prepare these rituals with the help of people outside their community. They have also lost all their internal capacities of conflict resolution. All medicinal plants growing along the river were lost due to the dam construction and the two Maya priests of the community as well as those who knew traditional healing systems were killed.	Colajacomo (1999)
Loss or threat to cultural identity	It also provoked the disintegration of their complex and delicate system of traditional and cultural internal regulation (loss of traditional law, of the role of the chief and of access to places of symbolic and religious importance).	Colajacomo (1999)
Loss or threat to cultural identity	loss of traditional economies, lands and housing	Colajacomo (1999)
Loss or threat to cultural identity	degenerative effect on the culture, economy and health of both displaced and still resident communities	Johnston (2010)
Loss or threat to cultural identity	Ethnographic research confirmed that Chixoy river basin communities had ancestral ties to the ceremonial complexes (Douzant Rosenfeld, 1988, 2003). The archaeological team contracted by INDE submitted proposals on three occasions (1980-1983) to modify the dam at a projected cost of $220,000 to rescue the Cauinal site and to minimise social impacts by allowing the	Johnston (2010)

	development of archaeo-tourism. These proposals were rejected. Cauinal remains in a deteriorated state, partially submerged for part of the year and totally submerged at other times.	
Loss or threat to cultural identity	During early construction, a French firm was hired to excavate Ancient Mayan objects, desecrating the sacred land in the eyes of the local communities.	GHRC (2011)
Loss or threat to cultural identity	Y allí hay mucho hallazgo arqueológico , porque una zona arqueológicamente rica tiene se habían registrado más 78 lugares muy importante y se encontraron un cementerio indígena	Interview
Loss or threat to cultural identity	threats to their culture and identities.	The Oakland Institute (2019)
Loss or threat to cultural identity	Today, rising tensions result often in violent confrontations between the Hamar and the army at Dimeka, where the community is resisting the villagization program, which they believe will eventually undermine their culture and traditional livelihoods.	Franchi and Manes (2016)
Loss or threat to cultural identity	pondrán en riesgo las practicas y conocimientos locales que han soportados sus medios de subsistencia y que los han mantenido en el territorio.	Roa Avendaño and Duarte Abadía (2012)
Loss or threat to cultural identity	the project owners original ly ignored the consequences the project would have on the 5,000 to 6,000 Cree and 3,500 Inuit whose fishing, hunting and trapping traditions would be disrupted.	Wall (2017)
Loss or threat to cultural identity	Impact on Setibeni Sheela, a religious site.	Thanju (2008)
Loss or threat to cultural identity	50 schools were closed. In Tete Province 25 schools were reported as completely destroyed with another 50 inundated.	Scudder (2005)
Loss or threat to cultural identity	people told her how they saw being “removed from their homes and forced to live in hostile environments” as an attack on their humanity (buntu) and their vital force (buumi)	Scudder (2005)
Loss or threat to cultural identity	Socio-cultural stress was primarily caused by loss of influence by individuals and loss of familiar cultural inventory which could have eased adjustment immediately after removal.	Scudder (2005)
Loss or threat to cultural identity	d'un sentiment réel de dépendance. Cette sensat entraîne vis-à-vis de tout et de tous une mentalité de sinistrés, d'assis et d'étrangers	PROWIZUR (1976)
Loss or threat to cultural identity	Ils s'y sentent donc toujours étrangers	PROWIZUR (1976)
Loss or threat to cultural identity	tous les sacrifices religieux relatifs au déménagement et aucun des arbres « Assyé » des villages déménagés n'a été planté	PROWIZUR (1976)
Loss or threat to cultural identity	Détérioration des relations entre les communautés villageoises	Raphaël et al (2019)

Loss or threat to cultural identity	the new reservoir is itself a physical disruption to community life: individual families and even whole villages have been disrupted and whole livelihoods have evaporated, while communities that used to share social ties are now cut off from each other by the reservoir.	International Rivers (2005)
Loss or threat to cultural identity	According to UNESCO, the Svan language is recognised as endangered and should be protected;	Green Alternative (2012)
Migration	There are no working opportunities in the area and men are forced to migrate to work in large plantations. Sometimes whole families migrate to the South for seasonal work, with all the problems that seasonal migration involves - a change of climate, working conditions, family disintegration.	Colajacomo (1999)
Migration	La gente se fue a la ciudad. Tuvo que partir hacia las ciudades cercanas: Bucaramanga o Barrancabermeja, donde viven sin mayores oportunidades laborales.	Roa Avendaño (2016)
Migration	otros han migrado a las ciudades en busca de empleo	Roa Avendaño and Duarte Abadía (2012)
Migration	Comme les terres vivrières produisent trop peu, les jeunes accueillis sont obligés de chercher du travail à l'extérieur	PROWIZUR (1976)
Migration	ces derniers sont victimes de l'exode rural ou se contentent des travaux manuels souv a ent mal rémunérés dans les plantations des utres, ce qui les rend très vulnérables vis à vis de leurs moyens d'existence.	Pittalunga (2002)
Migration	The adverse effects of the Kpong Dam were reported to have contributed to out-migration.	Owusu et al (2016)
Migration	Interviewees stated that they rely on remittances to meet their daily needs,	Human Rights Watch (2014)
Labour rights violation and safety issues		
Accidents & deaths at construction site	more than 100 workers were killed during the construction of the dam	Lang et al. (2000)
Accidents & deaths at construction site	Over 300 people were employed by the dam project but during the course of their work suffered occupational injuries	NAPE (2014)
Accidents & deaths at construction site	The complainants raised concerns on unpaid and poor compensations to the former workers who were discontinued from work upon sustaining serious accidents and injuries during their course of work at the Bujagali dam construction site. The victims were dismissed by Salini,	NAPE (2014)
Accidents & deaths at construction site	"Intanto sette persone sono morte negli ultimi tre anni durante i lavori di costruzione della diga" ha denunciato Narayan Gurung, leader del sindacato nazionale.	Medellin Mazzeo (2001)

Accidents & deaths at construction site	The dam was built in three years, largely by workers from China. Today, a stone memorial on top of the dam commemorates nineteen Chinese nationals who died during its construction; the memorial, inscribed in English and Chinese but not in Arabic, does not give the cause of their deaths. Alwash, the Iraqi-American hydrological engineer, told me that, in Iraq, when laborers fell into wet cement during large infrastructure projects, it was common for the work to carry on. “When you’re laying that much cement on a dam, you can’t stop,” Alwash said.	Filkins (2016)
Accidents & deaths at construction site	'missing' workmen who returned on duty after March 6 with injuries on their persons.	Tribune India (1999)
Accidents & deaths at construction site	a former Salini, SHE officer has alleged that an employee, a certain Mr Mungunda, broke several bones after falling several metres down the canyon.	Namibian Sun (2015)
Low or inadequate wages	Impregilo is accused by local and international trade unions of not respecting a joint agreement on wage and working conditions.	Lang et al. (2000)
Low or inadequate wages	Impregilo is accused by local and international trade unions of not respecting a joint agreement on wage and working conditions. In 1998, the Brussels-based International Confederation of Free Trade Unions reported that Impregilo, with the help of local police, locked out 5,000 workers. In addition, management and security forces physically abused the workers, large numbers of whom were arrested, and union leaders’ relatives were detained and some even tortured. On 22 December 1998, at Impregilo’s request, the Pakistani president implemented a Presidential Ordinance that deprived WAPDA workers of their rights to freedom of association and collective bargaining. The suspension of trade union rights of WAPDA’s 130,000 employees puts the company under the direct control of the armed forces.	Lang et al. (2000)
Low or inadequate wages	The complainants raised concerns on unpaid and poor compensations to the former workers who were discontinued from work upon sustaining serious accidents and injuries during their course of work at the Bujagali dam construction site. The victims were dismissed by Salini	NAPE (2014)
Low or inadequate wages	A portion of the civil servants’ salaries is deducted annually without their consent, a clear violation of workers’ rights.	International Rivers (2017)
Low or inadequate wages	Two inhabitants of the District of Nyangatom also suffered harassment when they had asked for a salary increase from the company Sisay Tesfaye Agroindustry in Kangaten	Franchi and Manes (2016)
Low or inadequate wages	The rally was part of a nation-wide protest call for which was given by the Construction Workers Federation of India against the police and administrative "repression let loose" between March 12 and March 19, 1999 on the striking Nathpa-Jhakri workers who were demanding equal wages for equal work.	Tribune India (1999)
Low or inadequate wages	The shop-stewards claimed that the company has failed to pay workers living at the campsite living allowances	Cloete (2016)

Low or inadequate wages	Local authorities in pre- and post-resettlement communities told Human Rights Watch that residents can find work on building projects at resettlement sites, and some men do obtain short-term work building their neighbors' houses. However, such work is by its nature temporary and low-paid.	Human Rights Watch (2014)
Poor or unsafe working conditions	Impregilo is accused by local and international trade unions of not respecting a joint agreement on wage and working conditions	Lang et al. (2000)
Poor or unsafe working conditions	las ofertas de empleo que hizo la empresa chocaron con las expectativas de la gente, pues era imposible cumplir con los requisitos de contratación exigidos: se les pedía una experiencia específica y larga en trabajos relacionados con la represa, debían pasar pruebas psicológicas, pagar por enviar sus hojas de vida y cumplir otras condiciones a las que no estaban acostumbradas las personas de la comunidad. De acuerdo a testimonios de la gente local, la respuesta de Isagén a esta situación fue contratar gente de otros lugares, la mayoría proveniente del departamento de Antioquia.	Roa Avendaño and Duarte Abadía (2012)
Poor or unsafe working conditions	Although the Karahnjukar Hydropower Project and the Alcoa smelting plant were both constructed and are operational at the time of this writing, they are still sharply criticized by environmentalists as detrimental to the highlands as well as being criticized for not fulfilling the promise of easing local unemployment by providing jobs	GNAD (2011)
Poor or unsafe working conditions	All'Impregilo, nello specifico, le organizzazioni sindacali imputano oltre che la responsabilità nell'adozione di insufficienti misure di sicurezza nel cantiere, il non riconoscimento dei minimi salVerdana, sans serifi a favore della manodopera impiegata	Medellin Mazzeo (2001)
Poor or unsafe working conditions	At the beginning Impregilo hired Icelanders to some extent but most-<br>ly Portuguese migrant workers through a Portuguese temporary staffing agency along with other migrant workers. The share of foreign workers at Kárahnjúkar steadily increased in the period, starting at 65% in the spring of 2003 and going up to 92% on estimate in July 2007 when the project was nearing its end.	Thorarins (2013)
Poor or unsafe working conditions	Road construction and other preparation work conducted by Icelandic contractors with Icelandic workers begun in late 2002 and Impregilo started their work in April 2003 stating the company planned to hire several hundred Icelandic workers in due course (Morgunblaðið 2003d). In reality only a fraction of Impregilo's workers were Icelandic, all the others were foreign migrants. Most plans made for the construction of the Kárahnjúkar power plant and the aluminum smelter in Reyðarfjörður had seriously underestimated the need of foreign labor predicting it to be from less than twenty percent and up to thirty percent at the highest. The reality turned out to be very different as most of the workers hired for the projects were	Thorarins (2013)

	foreign laborers who came to work temporarily and exclusively at these two worksites (Jóhannesson 2008).	
Poor or unsafe working conditions	The disputed construction project of Kárahnjúkar and the concomitant massive arrival of migrant workers recruited by temporary staffing agencies, figures highly into our analysis as it was critical for subsequent development of temporary staffing agencies in the country.	Thorarins (2013)
Poor or unsafe working conditions	The laud and bitter criticism relating to environmental concerns set aside, the abuse of foreign workers was no less fierce. Allegations of social dumping and other forms of workers abuse, appeared first among temporary workers at the Kárahnjúkar plant later to be detected elsewhere.	Thorarins (2013)
Poor or unsafe working conditions	Within the construction sector the state sponsored construction of a hydroelectric power plant in eastern Iceland called Kárahnjúkar proved to be a catalyst for economic expansion in the country. Moreover, this project played a key role in the development of TSA and the conflict over social dumping.	Thorarins (2013)
Poor or unsafe working conditions	failing to adhere to safety measures	Namibian Sun (2015)
Poor or unsafe working conditions	people working in the Fish River Canyon have no access to clean water or toilets. “People urinate in the river, while others drink that contaminated water, because it is too far to climb up to get water or a toilet,”	Namibian Sun (2015)
Poor or unsafe working conditions	Responding to workers’ allegations of poor working conditions in the blazing sun, and a lack of sanitation,	Namibian Sun (2015)
Poor or unsafe working conditions	She said workers have to climb the canyon walls without any safety aids. She also alleged that the workers receive no protective clothing and can hardly see a thing when the dust rises during work.	Namibian Sun (2015)
Poor or unsafe working conditions	12-hour shift	Lela Mobile Online (2016)
Poor or unsafe working conditions	Salini workers at the Neckartal Dam went on strike on Monday demanding better health and safety standards. The workers requested improved shade structures for lunch breaks, cool drinkable water and improved toilet facilities.	NND (2015)
Poor or unsafe working conditions	During May this year the workers on site threatened to down tools, accusing the company of unfair labour practices and poor working conditions.	New Era Live (2014)
Poor or unsafe working conditions	foreign workers employed without relevant work permits	New Era Live (2014)
Poor or unsafe working conditions	“The Italians have their own toilets that are clean and in good condition but workers are not permitted to use them,”	Tjihenuna (2014)

Poor or unsafe working conditions	their safety at the construction site is also compromised because no safety measures are followed, while their employers use derogatory racial remarks, labelling them “hungry Namibians”.	Tjihenuna (2014)
Poor or unsafe working conditions	Workers say the situation is worsened by the fact that they work on empty stomachs with no safe drinking water at the construction site despite the long extended working hours. Concerns of health hazards have also been raised, with workers saying there is only one mobile toilet shared by both men and women.	Tjihenuna (2014)
Threats and Violation of constitutional workers' rights	El gerente de Isagén dijo que éramos unos terroristas por haber secuestrado a los trabajadores de la obra el día del paro. Lo único que hicimos fue no dejarlos salir a trabajar ese día. Es la única manera que le pongan cuidado a uno	Roa Avendaño and Duarte Abadía (2012)
Threats and Violation of constitutional workers' rights	Un trabajador de la obra, habitante de la Playa, que Isagén había contratado hacía poco, tomó fotos de la muerte masiva de peces; esto condujo a que fuera despedido por la empresa.	Roa Avendaño and Duarte Abadía (2012)
Threats and Violation of constitutional workers' rights	The strike was in protest against the "reign of repression" let loose by administration on agitating workers at Nathpa Jhakri project.	Tribune India (1999)
Threats and Violation of constitutional workers' rights	He said the supervisors constantly threaten to ~re the workers for no good reason, and on top of that they use abusive language when speaking to them	Neckartal Dam workers claim victimisation by supervisors - New Era Live
Threats and Violation of constitutional workers' rights	Keetmanshoop Henima Investment (HI) employees deployed at the multi-billion-dollar Neckartal Dam claim they are constantly victimised and threatened by their Italian supervisors.	New Era Live (2016)
Threats and Violation of constitutional workers' rights	In their petition, the workers demanded the immediate removal of Industrial Relations Officer Michael Kadikwa, Velo and several other Italian supervisors they accuse of verbally abusing workers.	Lela Mobile Online (2016)
Threats and Violation of constitutional workers' rights	Metal and Allied Namibian Workers Union (Manwu) branch organiser Emmarentia Riekert charged that the company unlawfully fires workers on a daily basis	Lela Mobile Online (2016)
Threats and Violation of constitutional workers' rights	failure to respect the workers' constitutional rights	Cloete (2016)

Threats and Violation of constitutional workers' rights	“We are facing these each day of our lives. We can't speak to the Italians because they are rude, they shout at us, and never listen to what we have to say,” a disgruntled worker, who spoke on condition of anonymity for fear of victimisation, told The Namibian	Tjihenuna (2014)
Threats and Violation of constitutional workers' rights	WORKERS at Salini Impregilo, the Italian company contracted to the Neckartal Dam project near Keetmanshoop, are claiming abuse at the hands of their Italian employers, citing nepotism	Tjihenuna (2014)
Threats and Violation of constitutional workers' rights	workers expressed grievances over allegations of discrimination, human rights violations, victimisation, racism, and unfair dismissal, among a host of other things.	Tjihenuna (2014)
Repression of dissent		
Assassinations of env defenders	The intimidation campaign against the Maya Achì Indians began in 1980, following the community's refusal to move to the new settlements provided by INDE. In March 1980 seven people were killed during a meeting and in July 1980 the same fate befell two representatives who were going to claim titles to their land at INDE offices.	Colajacomo (1999)
Assassinations of env defenders	dictatorships deployed a policy of state-sponsored violence against a Mayan citizenry	Johnston (2010)
Assassinations of env defenders	aunque Isagen se ha comprometido a atender las inquietudes de las comunidades, la respuesta que han recibido estas últimas ante sus reclamos han sido frecuentes amenazas, incluso, después del asesinato de 6 integrantes del Movimiento Social en defensa del Río Sogamoso, muchos se intimidaron y desistieron de seguir abogando por sus derechos.	CENSAT-AguaViva (n.d.)
Criminalization of dissent	In 1998, the Brussels-based International Confederation of Free Trade Unions reported that Impregilo, with the help of local police, locked out 5,000 workers. In addition, management and security forces physically abused the workers, large numbers of whom were arrested, and union leaders’ relatives were detained and some even tortured. On 22 December 1998, at Impregilo’s request, the Pakistani president implemented a Presidential Ordinance that deprived WAPDA workers of their rights to freedom of association and collective bargaining. The suspension of trade union rights of WAPDA’s 130,000 employees puts the company under the direct control of the armed forces.	Lang et al. (2000)
Criminalization of dissent	Failure to implement a viable resettlement and remediation programme at the time of dam construction contributed to violence in the area.13 Communities that attempted to negotiate fair compensation were declared guerrilla-supporting communities, and military and civil patrols were used to remove people forcibly from the reservoir site. Guerrilla activity did occur	Johnston (2010)

	in this area, but it did not begin until well after dam construction had begun (Dill, 2004; Douzant Rosenfeld, 1988, 2003; Museo Comunita Río Rabinal Achi, 2003)	
Criminalization of dissent	Por todas estas denuncias que he hecho , yo me he encargado de hacer las denuncias , pues he sido objeto de demandas y entre ellas tenido cinco demandas , cuatro penales y una disciplinaria , con una modalidad que no existía casi en Colombia . Es que las empresas persuade a los militares para que me manden , para que haya mayor amedrentamiento entre ellos , dicen . De pronto , si es un militar el que lo demanda , pues la situación es mucho más grave y me han demandado dos militares , un coronel de la República y uno más importante , general de la República . Me puso una segunda demanda penal y clara por delitos , por ejemplo , como retención indebida de miembros de las fuerzas militares y lesiones agravadas contra dos miembros . Son de 38 años de presidio . Una segunda que era autor intelectual de invasión de tierras de campesinos , ya que son campesinos que salieron de esas tierras precisamente desalojado . Otra , que es obstrucción de vías que afectan la vía en las vías públicas , es otra , otra demanda y otra es autor intelectual de promover huelgas ilegales que afectan la seguridad del Estado , de las Fuerzas Militares y de la justicia colombiana . Bueno , en alguna oportunidad me preguntaba la prensa Usted cómo ve su viaje ? Yo pienso que el máximo guerrillero , este país que le dicen Timochenko pareciera que no es terrorista	Interview
Criminalization of dissent	The Ethiopian regime represses its citizens and has violated many human rights in its quest to construct the Grand Renaissance Dam. Citizens have not only been manipulated into buying bonds to support the project, they have also reported incidences of human rights abuses. The government has denied the public, including the affected people, information on the development (Nazret, 2015) and denied freedom of expression regarding GERD. A journalist was detained on May 4, 2013 for reporting on the return of thousands of farmers who had been forced from their lands in the Benishangul-Gumuz region – the same region where Ethiopia is constructing the multibillion dam. Another journalist was tried for terrorism and sentenced to two years jail for reporting on alleged coercion to force government employees to contribute to the construction of the dam (Opride, 2013). Citizens and civil society have no space to advocate for the respect of human rights or oppose government development without facing imprisonment or even death	International Rivers (2017)
Criminalization of dissent	The new law heavily restricts the thematic areas where civil society organizations can operate and places funding restrictions on local NGOs by international NGOs. The areas that are compromised include governance, civil society, and human rights issues.	International Rivers (2009)

Criminalization of dissent	Con l’obiettivo di limitare al minimo il dibattito civile sulle politiche controverse e censurare il dissenso, nel febbraio 2009 il governo etiope ha varato il decreto 621/2009. Il provvedimento impedisce a qualsiasi associazione o Ong locale che riceva più del 10% dei suoi finanziamenti da fondi esteri (quindi virtualmente tutte le associazioni esistenti nel paese) di lavorare in settori cruciali per la società civile tra cui quello dei diritti umani e della partecipazione democratica.	Survival International (2019)
Criminalization of dissent	Nel luglio 2009, l’ufficio giudiziario della regione meridionale ha revocato il riconoscimento a 41 “associazioni comunitarie”	Survival International (2019)
Criminalization of dissent	De la misma manera la criminalización indebida del líder sindical y ambiental Héctor Sánchez Gómez y el académico activista Miller Dussán	BHRRC (2017)
Criminalization of dissent	“El gerente de Isagén dijo que éramos unos terroristas por haber secuestrado a los trabajadores de la obra el día del paro. Lo único que hicimos fue no dejarlos salir a trabajar ese día. Es la única manera que le pongan cuidado a uno	Roa Avendaño and Duarte Abadía (2012)
Criminalization of dissent	un sacerdote de San Vicente de Chucurí, que tuvo siempre una posición clara frente a los daños sociales y ambientales que generaría la represa, fue enviado a Roma.	Roa Avendaño and Duarte Abadía (2012)
Criminalization of dissent	Opposition increased in June when one headman and his villagers refused outright to move to the Lusitu. Attempts to arrest him caused a riot during which PA staff and members of the Local Authority were forced from the village	Scudder (2005)
Criminalization of dissent	It is alleged that in the past few days 96 activists of the NJJU Workers Union had been summoned by the police at Jeori and Jhakri with directions to stop raising slogans regarding the missing workers.	Tribune India (1999)
Criminalization of dissent	The employees have been on suspension for their alleged involvement in the wildcat strike,	New Era Live (2017)
Criminalization of dissent	Eight activists arrested in Nenskra power plant protests	DFWatch (2020)
Criminalization of dissent	Leaders of the Chingwizi community who are in hiding said they were living in fear after families members told them that police wanted to arrest them all on charges of leading a rebellion against government resettlement plans	Human Rights Watch (2015)
Criminalization of dissent	On August 3, 2014, over 200 anti-riot police indiscriminately beat and arrested close to 300 people, mostly men, according to four eyewitnesses who were interviewed separately by Human Rights Watch. Two police Most of those arrested were later released, except for 29 who were charged with public violence. On August 4, government officials relocated the	Human Rights Watch (2015)

	camp clinic equipment and staff to Bongo and Nyoni sections. Lawyers representing those arrested alleged that police assaulted many of those whom they arrested—including chairperson of the Chingwizi camp committee, Mike Mudyanembwa. The government appears to have over-reacted to the protests and cracked down on some potentially innocent people. On August 8, a magistrate granted bail to 25, but kept the remaining four, including Mudyanembwa, in custody pending trial. While 25 were later freed by the court, the remaining four were convicted and sentenced to five years in prison on January 27, 2015.	
Violent repressive measures and militarization	In addition, management and security forces physically abused the workers, large numbers of whom were arrested, and union leaders' relatives were detained and some even tortured.	Lang et al. (2000)
Violent repressive measures and militarization	El movimiento mapuche es el ejemplo más claro de esta manipulación en donde el Estado –presionado por el capital privado- ha militarizado las comunidades llegando a invocar la Ley de Seguridad del Estado para justi~car el uso de la fuerza en los allanamientos a la propiedad privada.<br>El día de ayer en la visita de la presidenta a la inauguración de la Central Hidroeléctrica Angostura hubo situaciones de represión excesiva que no se condice con el lenguaje de los que habitamos este territorio. La sensación que quedó al ~nal del día fue un proceder –de las fuerzas de orden que se desplegaron"que violentó y redujo a los ciudadanos que quisieron proponer otra mirada al modelo actual del desarrollo, anulando cualquier voz que no fuese la del capital privado que ayer se dejó ver en todo su esplendor. El análisis en este sentido amerita a~nar el oído para entender el mensaje que el día de ayer la presidenta junto al capital privado entregó en el puel mapu	OLCA (2014)
Violent repressive measures and militarization	Tensions and suspicion also spread among the owners of lands and homes situated along the route where the high tension pylons had been built, connecting the power station with the capital. In 2009, for example, word was spread about some 176 homes being demolished along this route. To motivate this operation, the authorities explained that there was a high health risk. But a local satirical newspaper questioned the scientific grounds for this explanation, describing it as an urban myth. Since the owners of the houses in question affected by the demolition had obtained permission to build from the Ministry of Lands – the journalist wondered – who was responsible for this problem? Hence, the doubt was that the medical reason for demolishing the house might only be an excuse to 'disturb, harry and terrorize other people'	D'Angelo (2014)

Violent repressive measures and militarization	The construction company's directors therefore decided to hire mercenary troops to protect their staff and prevent further theft and destruction of equipment and machinery	D’Angelo (2014)
Violent repressive measures and militarization	another 84 Rio Negro people and were tortured and killed in Los Encuentros as were, subsequently, a further 35 orphans children in Agua Fria, another village in the vicinity, where they had found refuge after the Rio Negro massacres […] and nobody still knows what has happened to them. Fifteen women were forced to board a helicopter Thus, prior to dam completion and the resettlement of local residents, between February and September 1982, the death squads and the army killed about 400 men, women and children from Rio Negro, during mass or individual massacres. The attacks were officially declared by the government as being counterinsurgency activities. Between September 1981 and August 1983, about 4-5 thousand people were killed in the Rabinal department	Colajacomo (1999)
Violent repressive measures and militarization	hree women and some children succeeded to escape whereas 18 children in working age were kidnapped made slaves for many years, until they were freed (with the help of the catholic dioceses) and could relate what had happened.	Colajacomo (1999)
Violent repressive measures and militarization	In March, 70 women and 107 children were brought to the mountains a one-hour walk from Rio Negro in a place traditionally known as Pacoxon and there they were raped and killed	Colajacomo (1999)
Violent repressive measures and militarization	Community consultations occurred at a late stage, in the presence of armed soldiers,	Johnston (2010)
Violent repressive measures and militarization	Efforts to pursue justice through the court of public opinion, advocacy and media attention to that advocacy resulted (and still result in) in death threats and occasional acts of violence	Johnston (2010)
Violent repressive measures and militarization	Similar actions in a third village resulted in the detainment and torture of a community leader, as well as seizure of land title and compensation documents	Johnston (2010)
Violent repressive measures and militarization	l’unico torto della comunità locale di origine maya consisteva nell’opporsi alla realizzazione di una mastodontica diga sul fiume Chixoy.	Manes (2012)
Violent repressive measures and militarization	.. the military operation in question caused the death of more than 45 pastors Mursi and 39 Bodi while their flocks were grazing. Many people have been driven away and their animals stolen by soldiers. From February 2015, more than 120 people were killed and 265 arrested and held in the Jinka prison. Three young, educated Mursi, who had played the	Franchi and Manes (2016)

	role of representing their communities within the woreda, were fired on charges of having stirred up protests against resettlement.	
Violent repressive measures and militarization	According to the stories of the inhabitants of the Hamar woreda, in mid-July 2015 the federal police massacred more than a thousand local herders and many others were wounded. Twelve other people allegedly died in clashes between police officers and civil servants employed in Hamar villages.	Franchi and Manes (2016)
Violent repressive measures and militarization	In recent years, many have expressed about serious violations of human rights carried out in the area by the Ethiopian Executive branch to repress all forms of dissent	Franchi and Manes (2016)
Violent repressive measures and militarization	Local residents are forced to leave their homes and their lands by force, without receiving any compensation. Those who resist risk being imprisoned or tortured. This was what happened to four inhabitants in the area of Kara, who were held for two months because they had refused to leave their lands to the Turkish enterprise that operates in Kara Korcho.	Franchi and Manes (2016)
Violent repressive measures and militarization	violentó el derecho fundamental a la protesta social y a la libertad de expresión, en tanto que, mediante actos amenazantes y por la fuerza retiraron a los protestantes,	BHRRC (2017)
Violent repressive measures and militarization	cuando un funcionario de la empresa italiana Impregilo, contratista de Isagén, ordenó a la policía el desalojo violento de las personas que se encontraban protestando en la vía, de acuerdo a las denuncias del Movimiento Social en Defensa del Río Sogamoso, aunque la protesta se realizaba de manera pacífica frente a las instalaciones de los campamentos, los manifestantes fueron atacados por la policía con gases lacrimogenos y sus equipos y pancartas fueron destruidos por la fuerza pública.	Roa Avendaño and Duarte Abadía (2012)
Violent repressive measures and militarization	un profesor del colegio recibió amenazas de ser destituido de la escuela sí continuaba dando mensajes a la comunidad en contra de la empresa	Roa Avendaño and Duarte Abadía (2012)
Violent repressive measures and militarization	Small groups of protesters entered construction sites and stopped work for several hours throughout the summer of 2005 by chaining themselves to machinery and erecting a human blockade to prevent trucks from entering and exiting the site. There are some reports of police action against these protesters although it was not possible to substantiate statements about violence and intimidation being used to remove the protesters.	GNAD (2011)
Violent repressive measures and militarization	While on tour before the slaughter, the district office in charge asked an old man why he was armed with spears. The reply was “I am going to the war.” When asked who he was going to fight, the old man is said to given the district officer a friendly grin before replying “Why, you,	Scudder (2005)

	of course!" Subsequently the old man was killed after being shot in the chest by the mobile police (1959: 145).	
Violent repressive measures and militarization	While the police paraded along the floodplains of the Zambezi, the villagers held their own maneuvers nearby. They had spears, clubs and utilitarian and ceremonial axes. The police had guns. Failing to negotiate a solution to the impasse, the governor ordered the people into the lorries. The Gwembe men charged the mobile police who, believing their lives under threat, fired. Eight Gwembe Tonga were reported to have died and at least 32 were wounded.	Scudder (2005)
Violent repressive measures and militarization	The speakers criticised the role of management in beating up the missing workers who were then on strike.	Tribune India (1999)
Violent repressive measures and militarization	"You are looking for ways to fire rubber bullets at us. Police are interfering in the strike,"	Cloete (2016a)
Violent repressive measures and militarization	A 65-year-old man hiding in the mountains told Human Rights Watch that he had been separated from his wife and children as they fled police violence	Human Rights Watch (2015)
Violent repressive measures and militarization	about 30 police officers stationed at the camp attempted to quell the protests and then fled when violence broke out.	Human Rights Watch (2015)
Violent repressive measures and militarization	police forced all the women, including the sick and disabled, to sit in the sun without access to toilets for two consecutive days from 8 a.m. until 6 p.m. as punishment for being "rebellious" against government plans and for allegedly allowing some camp residents to burn the two police vehicles.	Human Rights Watch (2015)
Lack of transparency		
Lack or poor Information or involvement of local communities in the decision-making	"There was no participation," said Ejaz, and the lack of consultation fueled anxiety.	Bennet and McDowell (2012)
Lack or poor Information or involvement of local communities in the decision-making	Among the failings one official identified was the lack of involvement of those affected:	Bennet and McDowell (2012)

Lack or poor Information or involvement of local communities in the decision-making	and that the negotiations and discussions excluded women and the landless. Also severely disadvantaged were those whose livelihoods were directly tied to the river: fishermen, boatmen, and zarkash (gold panners).	Bennet and McDowell (2012)
Lack or poor Information or involvement of local communities in the decision-making	As well as lacking clear information, many villagers—whose communities had hardly changed in decades—said they found the concept of such a massive dam and loss of land almost unimaginable.	Bennet and McDowell (2012)
Lack or poor Information or involvement of local communities in the decision-making	Excluded from WAPDA's resettlement negotiations	Bennet and McDowell (2012)
Lack or poor Information or involvement of local communities in the decision-making	The authorities had deliberately not informed the people, because they were not giving them their full rights.	Bennet and McDowell (2012)
Lack or poor Information or involvement of local communities in the decision-making	We didn't have this in mind. We had ignored women . . . 50 percent of our population."	Bennet and McDowell (2012)
Lack or poor Information or involvement of local communities in the decision-making	problems with the consultative committee	NAPE (2014)
Lack or poor Information or involvement of local	The capacity of the Government of Sierra Leone to communicate was determined to be extremely limited. The information on development. The Role of Communication in Large Infrastructure was perceived as being incomplete, project-driven, and restricted to the President's and the government's political agenda	MAZZEI AND SCUPPA (2006)

communities in the decision-making	The overall level of credibility of public news was considered low. A majority of people did not trust the sporadic information provided by government because of the many broken promises of the past, and inflated official statements about the progress of projects.	
Lack or poor Information or involvement of local communities in the decision-making	when the dam and its related quarry were built, the local communities were not consulted, and remained uncompensated for land lost.	MAZZEI AND SCUPPA (2006)
Lack or poor Information or involvement of local communities in the decision-making	Y si no, la expropiaban. Nunca se dio chance de negociar.	SEMANA (2019)
Lack or poor Information or involvement of local communities in the decision-making	consultations took place only in 1976, after the dam construction had started. INDE representatives flew over Rio Negro by helicopter to inform people that a dam would be built and that the resulting reservoir would submerge their lands. Further contacts between INDE and local indigenous peoples took place in a climate of terror and intimidation. Rio Negro people were repeatedly threatened by INDE governmental representatives.	Colajacomo (1999)
Lack or poor Information or involvement of local communities in the decision-making	no consultation with local indigenous peoples was envisaged or undertaken in the planning of the Chixoy dam	Colajacomo (1999)
Lack or poor Information or involvement of local communities in the decision-making	No meaningful information was given to them	Colajacomo (1999)
Lack or poor Information or involvement of local communities in the decision-making	Construction proceeded without a comprehensive census of affected peoples or a plan to address compensation, resettlement and alternative livelihoods. Community consultations occurred at a late stage, in the presence of armed soldiers	Johnston (2010)

Lack or poor Information or involvement of local communities in the decision-making	A la mayoría faltaban los títulos de propiedad de las tierras e información sobre como obtener los resarcimientos.	Gerebizza (2009)
Lack or poor Information or involvement of local communities in the decision-making	usencia total de monitoreo y participación popular en las decisiones que eran tomadas precisamente por el Ministerio de la Economía y las Finanzas, en contacto directo con los funcionarios de Washington, pero no con las comunidades locales, campesinos y pueblos indígenas que habrían sufrido por decenas de años, los impactos económicos, ambientales y sociales de las grandes infraestructuras y de las importantes medidas político-económicas que el gobierno se comprometía a implementar con el Banco Mundial y la comunidad internacional de donadores.	Gerebizza (2009)
Lack or poor Information or involvement of local communities in the decision-making	sin la consulta e información previa a los afectados, así	Galindo Vanegas (2018)
Lack or poor Information or involvement of local communities in the decision-making	involuntary resettlement situation.	International Rivers (2017)
Lack or poor Information or involvement of local communities in the decision-making	yet its planning process has been top-down and unilateral. The public and dam-affected people have not been given a meaningful opportunity to critique the project or process.	International Rivers (2014)
Lack or poor Information or involvement of local communities in the decision-making	indigenous groups, who have been largely excluded from decision-making processes	Hodbod et al (2019)

Lack or poor Information or involvement of local communities in the decision-making	Obfuscation and distortion, if not outright fabrication – of the 'Public Consultation' process allegedly implemented by the EIA network of researchers, consultants and government-controlled local officials.	ARWG (2009)
Lack or poor Information or involvement of local communities in the decision-making	Awareness of the dam's impacts and development process is extremely low.	Allibhai (2015)
Lack or poor Information or involvement of local communities in the decision-making	We don't accept this. We disagree with whoever is planning this. We will never agree to it.	Allibhai (2015)
Lack or poor Information or involvement of local communities in the decision-making	Consultations with project-affected people in Kenya have never taken place.	International Rivers (2009)
Lack or poor Information or involvement of local communities in the decision-making	The majority of project-affected people (500,000) are located downstream of the dam site in the Lower Omo Valley and around Kenya's Lake Turkana. According to the project's Public Consultation and Disclosure Plan, only 93 members from four downstream indigenous communities were consulted. All downstream consultations occurred in 2007, after construction had already commenced.	International Rivers (2009)
Lack or poor Information or involvement of local communities in the decision-making	Anche se la costituzione etiope garantisce ai popoli indigeni il diritto alla "piena consultazione" e alla "espressione del proprio punto di vista nella pianificazione e attuazione di politiche e progetti ambientali che li riguardano", di fatto le comunità indigene vengono raramente consultate in modo appropriato.	Survival International (2019)
Lack or poor Information or involvement of local	L'accesso all'informazione pubblica è pressoché nulla perché pochi parlano l'amarico [la lingua nazionale] e il livello di alfabetizzazione è tra i più bassi d'Etiopia.<br>I funzionari di USAID che hanno visitato la bassa valle dell'Omo nel gennaio 2009 per valutare	Survival International (2019)

communities in the decision-making	l'impatto della diga Gibe III hanno reso noto che le comunità indigene locali non sapevano nulla o praticamente nulla del progetto.	
Lack or poor Information or involvement of local communities in the decision-making	The complaint criticises Salini for failing to seek the free, prior and informed consent of local people before building the dam	OECD Watch (2017)
Lack or poor Information or involvement of local communities in the decision-making	De acuerdo a testimonios de lideres de estas comunidades, se les ignora y a veces se les maltrata en los procesos de negociación.	Roa Avendaño and Duarte Abadía (2012)
Lack or poor Information or involvement of local communities in the decision-making	Los pequeños campesinos, las mujeres vendedoras de pescado y los mineros artesanales tienen condiciones económicas que les dan pocas posibilidades de inclusión en los proyectos de la empresa, lo que sugiere que el panorama de negociación para la población afectada continua siendo incierto y riesgoso.	Roa Avendaño and Duarte Abadía (2012)
Lack or poor Information or involvement of local communities in the decision-making	The Cree first heard about Bourassa's plan in day-old newspapers or over the radio.	Wall (2017)
Lack or poor Information or involvement of local communities in the decision-making	The work started in 1971 was done neither with regard to nor in consultation with the Cree communities in the region.	CNW (n.d.)
Lack or poor Information or involvement of local communities in the decision-making	Local people complain that they were given very little information…	Scudder (2005)

Lack or poor Information or involvement of local communities in the decision-making	Resettlers included whole villages or village sections that were involuntarily moved because of dam construction and reservoir formation as well as individual households required to move for such reasons as the construction of road and power lines.	Lenka Thamae and Pottinger (2006)
Lack or poor Information or involvement of local communities in the decision-making	Nor did the government provide people with an opportunity to meaningfully participate in the crafting of the proposed resettlement plan or facilitate legal, technical and other advice to people about their rights and options.	Human Rights Watch (2014)
Lack or poor Information or involvement of local communities in the decision-making	The government has not taken sufficient steps to provide families awaiting resettlement and those who have already resettled with timely, accurate, and specific information about certain aspects of the resettlement process such as compensation procedures, complaint mechanisms, availability of land for farms and pastures, employment opportunities, and other assistance.	Human Rights Watch (2014)
Lack or poor Information or involvement of local communities in the decision-making	The participation of the population in the project benefits was not achieved to the desired extent.	KFW (2006)
Lack or poor Information or involvement of local communities in the decision-making	The Masvingo Zanu PF leadership is however said to have engaged Triangle Sugar Limited to form a joint venture company to grow cane on the land meant for the displaced.<br>"The people are just being given half a hectare of dry land plots to pacify them. We are concerned that lack of consultation with DTZ could be evidence that the current developments constitute land invasions disguised as land compensation for people from Tokwe Murkosi," said a senior DTZ official.	Mambo (2014)
Lack or poor Information or involvement of local communities in the decision-making	Flood victims do not want, and say they never agreed, to venture into commercial sugar cane farming.	Human Rights Watch (2015)
Lack or poor Information or involvement of local	Local residents were not adequately consulted on their needs or on the effect that the dam would have on their lives	Human Rights Watch (2015)

communities in the decision-making		
Lack or poor Information or involvement of local communities in the decision-making	There was little consultation even prior to the flood about whether it was beneficial to undertake the development and uproot people this distance.	Human Rights Watch (2015)
Corruption - alleged or proven	The project hit the business newspaper headlines in August 1999 after the Lesotho government accused Masupha Sole, the former CEO of LHDA, of taking nearly $2 million in bribes from ten companies and two consortia.	Lang et al. (2000)
Corruption - alleged or proven	Swiss newspaper Sonntags Zeitung reported that a court there found evidence that the 12 companies had paid money into Sole's bank accounts — payments that Sole could not adequately explain. In November 1999, the Lesotho authorities announced that the companies were to be prosecuted for having • wrongfully, unlawfully and corruptly made payments/transfers• to Sole.	Lang et al. (2000)
Corruption - alleged or proven	The Yacyreta dam was famously described by Argentinian president Carlos Menem as a • monument to corruption	Lang et al. (2000)
Corruption - alleged or proven	Grievances included corruption	Bennet and McDowell (2012)
Corruption - alleged or proven	When they were evaluating the houses they favored those who had bribed them. In case of those who did not bribe, they gave a very low price for their houses	Bennet and McDowell (2012)
Corruption - alleged or proven	It is also interesting to note that the local population was convinced that the dam belonged to the same construction company. As Mazzei and Scuppa observe (2006), the latter had sought to build up over the years a relationship of 'good neighbourliness' with the inhabitants of the villages near the dam, making gifts of various kinds. In this way a paternalistic relationship in which the local communities 'were begging the constructor instead of asking for their rights, of which they were not fully aware' (Mazzei and Scuppa 2006: 16) was, however, set up.	D'Angelo (2014)
Corruption - alleged or proven	there was a widespread opinion among the inhabitants of the Tonkolili District that the works to complete the power station had been deliberately slowed down by the Italian constructors themselves (Mazzei and Scuppa 2006). In this way they would have had more time to secretly mine and smuggle out the gold and diamonds found during the construction of the dam.	D'Angelo (2014)
Corruption - alleged or proven	Another factor was widespread mistrust of "corrupt politicians." Because of its long and controversial history, Bumbuna Hydro was held up as an example of mismanagement, corruption, and the government's failure to deliver on its promises.	MAZZEI AND SCUPPA (2006)

Corruption - alleged or proven	Many believed that the Italian contractors (Salcost), with the agreement of some corrupt members of the government, would have voluntarily delayed the completion of the dam to be able to mine the site and smuggle gold and diamonds	MAZZEI AND SCUPPA (2006)
Corruption - alleged or proven	Salcost made "presents" to local communities, but with two negative effects.10<br>First, this developed a paternalist relationship between the contractor and local communities, which were begging the constructor instead of asking for their rights, of which they were not even fully aware. Second, it created a misperception that Salcost was the "owner" of the BHP, and that the government had no responsibilities toward the affected communities.<br>Rumors.	MAZZEI AND SCUPPA (2006)
Corruption - alleged or proven	corruption that played a crucial role. Various sources estimate that between US$300 and US$500 million dollars were lost to corruption on this project. "The dam was the biggest gold mine the crooked generals ever had," according to Rafael Bolaños, dean of the School of Civil Engineering at Guatemala's San Carlos University.	Colajacomo (1999)
Corruption - alleged or proven	también se descubrió algo muy grave y es que se utilizaron abogados muy poderosos en Colombia que están vinculados con las altas cortes . Fueron contratados por la misma compañía y esos abogados fueron los que intervenían en las mismas cortes y persuadía a los jueces para que me	Interview
Corruption - alleged or proven	According to Transparency International, large public works projects are one of the world's most corrupt sectors, and no-bid contracts are an open invitation to corruption.	International Rivers (2009)
Corruption - alleged or proven	Nello specifico alcuni ufficiali statali sono stati accusati di gravi atti di corruzione: "con l'avvio dei lavori di realizzazione della diga di Kaligandaki essi si sono rapidamente arricchiti, divenendo proprietari di ville con piscine e di alcune aziende". "Nulla è stato speso invece – prosegue la denuncia delle organizzazioni in lotta - per creare misure di protezione ambientale, nonostante sia stato provato che i progettisti non si sono attenuti agli standard minimi richiesti dalle normative"	Medellin Mazzeo (2001)
Corruption - alleged or proven	the premier's special adviser had reportedly asked a union boss to help with a byelection on the same day he offered a monopoly over work at James Bay. The inquiry concluded that union corruption could not have been so deeply entrenched unless it had been actively supported by the government. "A devastating document," proclaimed a Gazette editorial. "For some four years, the Bourassa government worked hand in glove with gangster union leadership in the province's construction industry."<br>There were tales of nepotism, bribery, sabotage, blackmail and intimidation; charges of union organizers with criminal records	Curran (2012)

Corruption - alleged or proven	Impregilo is accused of having given bribes amounting to at least US$250,000 to the former CEO of the Lesotho Highlands Development Authority.	International Rivers (1999)
Corruption - alleged or proven	Over the past week, the companies were served with summons to appear before the Maseru Magistrate's Court on November 29 on charges of corruption.	International Rivers (1999)
Corruption - alleged or proven	corruption and mismanagement of the funds allocated for the dam's conservation turned the dam into a time bomb.	Bibbo (2016)
Corruption - alleged or proven	assenza di una gara d'appalto (immaginiamo cosa questo può voler dire con un governo, come quello iracheno, tra i più corrotti del pianeta)	InfoAut (2016)
Corruption - alleged or proven	A worker, who did not want his name published, accused the company's Human Resources Manager, Hendrina Mukwiilongo, of receiving bribes from workers to double their salaries and employ their relatives at the site.	Namibian Sun (2015)
Corruption - alleged or proven	The congress condemned the fact that a number of pro-dam activists were being paid salaries by Salini Impregilo, the Italian company which is responsible for the project's implementation	DFW (n.d.)
Corruption - alleged or proven	En bancos suizos se ha detectado el tránsito de al menos 235 millones de dólares, en su mayoría de coimas vinculadas al proyecto hidroeléctrico Tocoma	Poliszuk et al (2018)
Corruption - alleged or proven	Se registraron cerca de 180 millones USD entre 2010 y 2015, provenientes de una cuenta del Consorcio OIV-Tocoma en el banco Banesco, Panamá, con la referencia de 'purchase material' [materiales de compra]".	Poliszuk et al (2018)
Corruption - alleged or proven	Según una Comisión Mixta formada por la Asamblea Nacional en 2017, el mayor peso sobre el caso Tocoma recae en el Consorcio OIV, del cual un 50% de sus acciones corresponde a Odebrecht. Dicha comisión determinó que se había producido un desfalco de más de 3.000 millones de dólares	El Pitazo (2019)
Corruption - alleged or proven	During his presidential campaign, Argentina's Carlos Menem called Yacyretá "a monument to corruption." But despite well–documented allegations implicating engineering and construction companies and politicians in siphoning off public funds in the building of Yacyretá, no one has ever been brought to justice	International Rivers (n.d./a)
Corruption - alleged or proven	evidence of corruption	NAPE (2014)
Corruption - alleged or proven	The Lesotho government has taken four British business groups to court, charging them with paying bribes to the chief executive, Masupha Sole, of the Lesotho Highlands Water Authority. The companies, Balfour Beatty, Sir Alexander Gibb and Co, Stirling Intemational Civil Engineering and Kier Intemational are charged alongside the Swiss-Swedish group, ABB, Impregilo of Italy, Acres Intemational of Canada and Sogreah, Dumez and Cegelec of France	Lesotho_Bracking

Corruption - alleged or proven	Following the trial of Lahmeyer, Jacobus Michiel Du Plooy, a 'middle man' responsible for the flow of corrupt payments to corporations working on the LHWP, pleaded guilty. His evidence assisted the prosecution in its pursuit of the Italian corporation Impregilo, the lead corporation in the Highlands Water Venture Consortium (HWV). Impregilo had restructured itself, arguably, in an attempt to avoid prosecution. The company sought unsuccessfully to avoid trial by a number of artful arguments about the serving of the summons, the personal liability of employees for actions taken during the course of their employment and the jurisdiction of the court.6 In September 2006 Impregilo acknowledged the failure of its evasive tactics and pleaded guilty to 'attempting to defeat the course of justice'. The company was fined M15 million (over US $2 million).	LesothoHWP_2007_GCR_EN
Corruption - alleged or proven	The corporate defendants in these proceedings did everything within their powers to avoid justice taking its course. Some met privately together in 1999 to discuss the tactics they would employ to fend off conviction.	LesothoHWP_2007_GCR_EN
Corruption - alleged or proven	When they were evaluating the houses they favored those who had bribed them. In case of those who did not bribe, they gave a very low price for their houses . . .	Bennet and McDowell (2012)
Corruption - alleged or proven	It is also interesting to note that the local population was convinced that the dam belonged to the same construction company. As Mazzei and Scuppa observe (2006), the latter had sought to build up over the years a relationship of 'good neighbourliness' with the inhabitants of the villages near the dam, making gifts of various kinds. In this way a paternalistic relationship in which the local communities 'were begging the constructor instead of asking for their rights, of which they were not fully aware' (Mazzei and Scuppa 2006: 16) was, however, set up.	D'Angelo (2014)
Corruption - alleged or proven	there was a widespread opinion among the inhabitants of the Tonkolili District that the works to complete the power station had been deliberately slowed down by the Italian constructors themselves (Mazzei and Scuppa 2006). In this way they would have had more time to secretly mine and smuggle out the gold and diamonds found during the construction of the dam.	D'Angelo (2014)
Corruption - alleged or proven	Another factor was widespread mistrust of "corrupt politicians." Because of its long and controversial history, Bumbuna Hydro was held up as an example of mismanagement, corruption, and the government's failure to deliver on its promises.	MAZZEI AND SCUPPA (2006)
Corruption - alleged or proven	Many believed that the Italian contractors (Salcost), with the agreement of some corrupt members of the government, would have voluntarily delayed the completion of the dam to be able to mine the site and smuggle gold and diamonds	MAZZEI AND SCUPPA (2006)
Corruption - alleged or proven	Salcost made "presents" to local communities, but with two negative effects. First, this developed a paternalist relationship between the contractor and local communities, which were begging the constructor instead of asking for their rights, of which they were not even	MAZZEI AND SCUPPA (2006)

	fully aware. Second, it created a misperception that Salcost was the “owner” of the BHP, and that the government had no responsibilities toward the affected communities.<br>Rumors.	
Corruption - alleged or proven	corruption that played a crucial role. Various sources estimate that between US$300 and US$500 million dollars were lost to corruption on this project. "The dam was the biggest gold mine the crooked generals ever had," according to Rafael Bolaños, dean of the School of Civil Engineering at Guatemala's San Carlos University.	Colajacomo (1999)
Corruption - alleged or proven	también se descubrió algo muy grave y es que se utilizaron abogados muy poderosos en Colombia que están vinculados con las altas cortes . Fueron contratados por la misma compañía y esos abogados fueron los que intervenían en las mismas cortes y persuadía a los jueces para que me	Interview
Corruption - alleged or proven	According to Transparency International, large public works projects are one of the world’s most corrupt sectors, and no-bid contracts are an open invitation to corruption.	International Rivers (2009)
Corruption - alleged or proven	Nello specifico alcuni ufficiali statali sono stati accusati di gravi atti di corruzione: “con l’avvio dei lavori di realizzazione della diga di Kaligandaki essi si sono rapidamente arricchiti, divenendo proprietari di ville con piscine e di alcune aziende”. “Nulla è stato speso invece – prosegue la denuncia delle organizzazioni in lotta - per creare misure di protezione ambientale, nonostante sia stato provato che i progettisti non si sono attenuti agli standard minimi richiesti dalle normative”	Medellin Mazzeo (2001)
Corruption - alleged or proven	the premier’s special adviser had reportedly asked a union boss to help with a byelection on the same day he o~ered a monopoly over work at James Bay. The inquiry concluded that union corruption could not have been so deeply entrenched unless it had been actively supported by the government. “A devastating document,” proclaimed a Gazette editorial. “For some four years, the Bourassa government worked hand in glove with gangster union leadership in the province’s construction industry.”<br>There were tales of nepotism, bribery, sabotage, blackmail and intimidation; charges of union organizers with criminal records	Curran (2012)
Corruption - alleged or proven	Impregilo is accused of having given bribes amounting to at least US$250,000 to the former CEO of the Lesotho Highlands Development Authority.	International Rivers (1999)
Corruption - alleged or proven	Over the past week, the companies were served with summons to appear before the Maseru Magistrate’s Court on November 29 on charges of corruption.	International Rivers (1999)
Corruption - alleged or proven	corruption and mismanagement of the funds allocated for the dam's conservation turned the dam into a time bomb.	Bibbo (2016)

Corruption - alleged or proven	assenza di una gara d'appalto (immaginiamo cosa questo può voler dire con un governo, come quello iracheno, tra i più corrotti del pianeta)	InfoAut (2016)
Corruption - alleged or proven	A worker, who did not want his name published, accused the company's Human Resources Manager, Hendrina Mukwiilongo, of receiving bribes from workers to double their salaries and employ their relatives at the site.	Namibian Sun (2015)
Corruption - alleged or proven	En bancos suizos se ha detectado el tránsito de al menos 235 millones de dólares, en su mayoría de coimas vinculadas al proyecto hidroeléctrico Tocoma	Poliszuk et al (2018)
Corruption - alleged or proven	During his presidential campaign, Argentina's Carlos Menem called Yacyretá "a monument to corruption." But despite well–documented allegations implicating engineering and construction companies and politicians in siphoning off public funds in the building of Yacyretá, no one has ever been brought to justice	International Rivers (n.d./a)
Contract- and bidding related issues	By a special clause in the contract with the GOE, Salini also had no financial liability for the project.	Carr (2017)
Contract- and bidding related issues	EEPCO repeated its turnkey contracting— disregard for bidding process and project oversight—in successive Gibe dam contracts with Salini.	Carr (2017)
Contract- and bidding related issues	The EEPCO issued an Engineering Procurement Construction (EPC) contract to the Italian-based Salini Costruttori—a 'turnkey' contract for a completed project, eliminating any oversight during the construction period.	Carr (2017)
Contract- and bidding related issues	La obra es la primera que se vende bajo el esquema de subasta por cargo de confiabilidad, que significa que a Emgesa se le garantiza "un ingreso fijo independiente de su participación diaria en el mercado mayorista asignado por un período de 20 años, reduciendo así el riesgo de su inversión", pago que se suma a la factura de los usuarios.	Dussán Calderón (2016)
Contract- and bidding related issues	ellos solo se dedican a construir las obras , que fue aprobada la licencia , pero no repararon en absoluto en la situación de la zona .	Interview
Contract- and bidding related issues	incumplimiento de la normatividad relacionada con los procesos de adjudicación del proyecto	Galindo Vanegas (2018)
Contract- and bidding related issues	La adjudicación del proyecto a Emgesa mediante una subasta realizada con una única empresa proponente -Emgesa-	Galindo Vanegas (2018)
Contract- and bidding related issues	In a flawed bidding process (http://countryoutreach.realityofaid.org/roareports/download/file/Q2dLNWhkWFU2dEd0ZDZ5bEpkeU5sZ0dZbGFVc2NVTmhtS3QveS9xTGN1eXlje Ethiopia granted the project to a Milan-based engineering company, Salini Costruttori, circumventing its own contract procedures and international standards on procurement.	Hussein (2014)

Contract- and bidding related issues	In July 2006, the government of Ethiopia directly awarded a no-bid Engineering, Procurement & Construction (EPC) contract for Gibe 3 to Italian construction company Salini	International Rivers (2009)
Contract- and bidding related issues	Il contratto è stato concluso senza gara d’appalto in violazione delle leggi etiopi	Survival International (2019)
Contract- and bidding related issues	The owners of the dam have consistently worked to maximize power output and minimize mitigation efforts. In spite of extensive research concerning the project’s impact on endangered and threatened species existing within the Gorge, project authorities and donors refuse to implement necessary mitigation measures. In fact, TANESCO is operating the dam without rights to the Kihansi River’s water, so it does not even have permission to divert any water from the Gorge. The Government of Tanzania is not forcing TANESCO to comply with existing water rights because of demands for power.	International Rivers (2001)
Contract- and bidding related issues	assenza di una gara d'appalto (immaginiamo cosa questo può voler dire con un governo, come quello iracheno, tra i più corrotti del pianeta)	InfoAut (2016)
Contract- and bidding related issues	The key contractual terms of the project remain unclear as the agreement between the government and the investor is confidential. The public is uninformed about the government’s guaranteed power purchase agreement, tariffs, taxes, land ownership and other terms that have significant consequences for the individual consumer	Green Alternative (2012)
Poor or no E(S)IA	In the revised 1993 project environmental summary, the emphasis is on studying the impacts of the Caruachi dam solely in the area between the Guri and Tocoma reservoirs. From a hydrological and ecological point of view, this analysis is both unreasonable and illogical. As the Caruachi project is part of a larger scheme, the Norwegian NGO FIVAS, is calling for the cumulative effects of the entire development to be evaluated.	Lang et al. (2000)
Poor or no E(S)IA	No comprehensive environmental impact assessment was ever made, however, nor were erosion or sedimentation studies conducted for Phase 1A. The downstream impacts also appear to have been overlooked	Lang et al. (2000)
Poor or no E(S)IA	t is known that fish migrate up and down the river several times during the course of their lives. • This aspect was not considered,• according to an internal World Bank evaluation of Yacyreta. So the Yacyreta’s fish elevators only carry fish up-river. Those fish that do manage to negotiate the elevator and reach the reservoir face an unpromising future — when the first turbine was opened in 1994, more than 120,000 dead fish were found downstream, thought to have been killed by the lack of oxygen in the reservoir caused by rotting vegetation	Lang et al. (2000)

Poor or no E(S)IA	Varios años han pasado desde que este proyecto ingresara al sistema de evaluación ambiental, y pese a la inadmisibilidad que el estudio de impacto ambiental presentado contenía, este fue acogido por la legislación de esa época sin reparo alguno.	OLCA (2014)
Poor or no E(S)IA	An analysis of the risks of climate change on Uganda's energy sector and its economy has not been undertaken	NAPE (2014)
Poor or no E(S)IA	BEL's Power Purchase Agreement (PPA 2007) economic analysis does not adequately address the economic viability in relation to hydrological risks, social and environmental impacts. The report only highlights the benefits and not the costs associated with change in water flows and disruption of people's livelihoods of lake-side dwellers and businesses.	NAPE (2014)
Poor or no E(S)IA	BEL's Social and Environmental Assessment (SEA) does not adequately address the questions about hydrological changes on power generation at the Nalubaale, Kiira and the Bujagali dam	NAPE (2014)
Poor or no E(S)IA	The lack of up-to-date and adequate information on hydrology, climate change, cumulative impacts assessments and commitment on Kalagala "Off Set" in BEL's SEA violates the African Development Bank Policies on Environmental Assessment	NAPE (2014)
Poor or no E(S)IA	The CRBM pointed out, for example, that the Ethiopian EPA did not produce an environmental or socioeconomic impact report (EIA) prior to the development	Carr (2017)
Poor or no E(S)IA	During the feasibility study, "no consideration was given to the possible use of other types of energy such as solar or geothermal"	Colajacomo (1999)
Poor or no E(S)IA	Neither the World Bank nor INDE have ever carried out environmental impact assessments or protection plans.	Colajacomo (1999)
Poor or no E(S)IA	a Via non contiene uno studio sui processi di sedimentazione del bacino e proposte adeguate per la gestione dei sedimenti. Manca una valutazione dettagliata degli impatti del progetto sull'ecosistema nella zona del bacino, inclusa la produzione di gas serra, gli effetti sulla biodiversità biologica in particolare riguardo le specie minacciate o in pericolo.	Manes (2012)
Poor or no E(S)IA	Le informazioni presentate sono insufficienti e poco rappresentative al fine di prevenire i potenziali impatti sulla qualità dell'acqua. Lo studio omette una valutazione dettagliata e non offre una strategia adeguata di mitigazione.	Manes (2012)
Poor or no E(S)IA	Lo studio non presenta un'analisi dettagliata del possibile impatto del progetto sulla salute e il benessere della popolazione nel breve e nel lungo periodo.	Manes (2012)
Poor or no E(S)IA	Lo studio non presenta un'analisi dettagliata delle alternative.	Manes (2012)
Poor or no E(S)IA	Lo studio presenta errori metodologici, in particolare l'omissione della scala e della misura-zione degli impatti.	Manes (2012)
Poor or no E(S)IA	Non è stato menzionato nessun riferimento né al piano di gestione ambientale, né alle misure di mitigazione.	Manes (2012)

Poor or no E(S)IA	en la fase de planeación los beneficios del proyecto habían sido tal vez exagerados. Por ejemplo, en los contratos de crédito se estableció el objetivo de “poner en producción bajo riego de 100.000 hectáreas, de las cuales 50.000 en ambas márgenes del rio Daule”. El Informe subraya en cambio que “en la realidad solo se ejecutaron 17.000 hectáreas” y “tampoco se ocurrieron los beneficios de navegación, recreación y turismo, que fueron previstos. De hecho, occurrió todo lo contrario”	Gerebizza (2009)
Poor or no E(S)IA	La construcción de la represa ha bloqueado casi toda la afluencia de las aguas de los dos ríos, causando por tanto, impactos importantes aguas abajo, los cuales nunca han sido considerados.	Gerebizza (2009)
Poor or no E(S)IA	Emgesa, con el aval de la ANLA, procedió al llenado del embalse sin tener en cuenta las recomendaciones anteriores y cuando aún no había concluido el estudio que contrató. Pos	Dussán Calderón (2016)
Poor or no E(S)IA	encontramos varias falencias graves en el documento que sirvió de base para la aprobación de la licencia ambiental para su construcción, la evaluación de impacto ambiental (EIA).	Dussán Calderón (2016)
Poor or no E(S)IA	La Asociación de Afectados por el Proyecto Hidroeléctrica El Quimbo -Asoquimbo- ha demostrado que el problema se originó en el desconocimiento por parte del Gobierno del presidente Alvaro Uribe del Auto No. 517 de 31 de julio de 1997 mediante el cual El Ministerio de Ambiente declaró no viable el Proyecto Hidroeléctrico El Quimbo, sin embargo, en el 2007 subastó el Proyecto a Emgesa y declaró mediante Auto No 515 del 22 de febrero de 2008 que El Quimbo “No requería de la presentación de Diagnóstico Ambiental de Alternativas (DAA) y otorgó Licencia Ambiental Mediante Resolución 0899 del 15 de mayo de 2009.	Dussán Calderón (2016)
Poor or no E(S)IA	falta de planificación del proyecto y en consecuencia por los efectos no esperados de la construcción de la hidroeléctrica -aumento de la criminalidad, aumento de la demandad de instalaciones penitenciarias, inundación de la infraestructura vial, impactos sanitarios, desempleo, falta de capacitación para el aprovechamiento turístico del embalse por parte de los habitantes de Hobo y Yaguará-, que llevaron a los municipios a vivir situaciones que pudieron haberse evitado con un buen ejercicio de planificación.	Galindo Vanegas (2018)
Poor or no E(S)IA	Impactos “No Previstos” en los Estudios de Impacto Ambiental como por ejemplo "el desconocimiento por parte de EMGESA de las actividades económicas de los apicultores, lecheros, comerciantes, tenderos y transportadores”	Galindo Vanegas (2018)
Poor or no E(S)IA	Riesgos de la hidroeléctrica no identificados en los estudios de Impacto Ambiental:	Galindo Vanegas (2018)
Poor or no E(S)IA	Ethiopia disputes any harm will be caused, but to date, the government has made public no Environmental and Social Assessment study.	International Rivers (2017)

Poor or no E(S)IA	The panel found numerous important gaps in the project documentation, and noted: "The (hydrological study) is very basic, and not yet at a level of detail, sophistication and reliability that would befit a development of this magnitude, importance and with such regional impact as GERD."	International Rivers (2014)
Poor or no E(S)IA	After thorough evaluation of the 'Downstream EIA', there is consensus among ARWG members that the document rests on a series of faulty premises and that it is further compromised by pervasive omissions, distortions and obfuscation.	ARWG (2009)
Poor or no E(S)IA	The quantitative (and qualitative) data included in virtually all major sections of the report were clearly selected for their consistence with the predetermined objective of validating the completion of the Gibe III hydrodam.	ARWG (2009)
Poor or no E(S)IA	The ESIA is largely based on insufficient scientific analysis and a lack of evidence. It quickly concludes that numerous impacts, including to local communities and protected areas, are negligible. Risks to health and livelihoods of affected communities are particularly poorly addressed. Mitigation measures are inadequate, unrealistic and do not acknowledge the failure of similar mitigation measures at other dams in Ethiopia. The January 2009 ESIA includes new sections on project alternatives and basin-wide cumulative impacts, which further demonstrate simplistic analysis and an attempt to provide supporting evidence for the dam.	International Rivers (2009)
Poor or no E(S)IA	The GOE discounts the seismic danger to the planned Gibe III dam, ignoring key geological information. The international development banks and bilateral agencies engaged with feasibility and impact studies as well as funding of the project—directly and indirectly—also ignore available data pointing to major seismic risk.	Carr (2017)
Poor or no E(S)IA	Survival International maintains that Salini knew or should have known about the dams impacts on downstream communities if had conducted due diligence and had carried out proper impact assessments.	OECD Watch (2017)
Poor or no E(S)IA	incumplimiento de la licencia ambiental	CENSAT-AguaViva (n.d.)
Poor or no E(S)IA	nada de esto se está considerando durante las evaluaciones de impacto ambiental, otorgándose, finalmente, las licencias ambientales	Roa Avendaño and Duarte Abadía (2012)
Poor or no E(S)IA	Information officer at Landsvirkjun Magnús Þór Gylfason pointed out that the report isn't finished. He also stated that the environmental impact assessment (EIA) of the dam had warned before its construction that conditions of the lake's biosphere would worsen with increased flow of water.	IRO (2013)

Poor or no E(S)IA	the only reference to resettlement in Federal Power Board and World Bank documents was budgeting for the removal of 29,000 people as opposed to the 57,000 eventually resettled	Scudder (2005)
Poor or no E(S)IA	The engineers ignored the cities of Trabzon, Kars, Erzurum, Van, and Mardin, despite the parliamentary commission report asserting that the Keban Dam was "the key project" for the development of that part of the country. Of the fourteen provinces making up the Eastern Anatolia region, only two were part of the study area, while just half of the Southeastern Anatolia region was included. Moreover, the study area included only five of the seventeen provinces considered to form Northern Kurdistan (Culcasi, 2006)	Stahl (2019)
Poor or no E(S)IA	the report does not discuss the social and environmental effects of huge reservoirs that would flood hundreds of square kilometers or the myriad health effects of irrigation, already well-known by the mid-1960s.	Stahl (2019)
Poor or no E(S)IA	No EIA was carried out before the project began. The World Bank conducted an environmental assessment as part of the project feasibility study in the early 1990s. When NORAD joined the project in 1994, it found the World Bank's environmental assessment so deficient that it financed an EIA in 1995. This EIA was also found to be of very poor quality because it contained inadequate data and failed to include adequate water– discharge and dam management plans. The Kihansi spray toad and two rare plants were discovered in 1996, one year after dam construction began. For unknown reasons, TANESCO was not informed of this development until 1998.	International Rivers (2001)
Poor or no E(S)IA	The pending extinctions are a direct result of the World Bank's failure to require a thorough Environmental Impact Assessment before it financed the LKHP.	International Rivers (2001)
Poor or no E(S)IA	The LHWP began without an environmental impact assessment (EIA) for the overall project.	International Rivers (2005)
Poor or no E(S)IA	The ESIA concludes that only 80 families in both gorges would be directly impacted by the project, therefore the social impacts are not that significant. According to estimates from Green Alternative and Bankwatch, the project's impacts will be much broader and encompass segments of population for which social consequences have been unaccounted for, such as women, the elderly and internally displaced people. The ESIA also fails to recognize these groups as vulnerable, despite they are to suffer most profoundly from the loss of land and changes to their traditional livelihoods.	Green Alternative (2012)
Poor or no E(S)IA	The ESIA does not recognize Svans as indigenous peoples, on the grounds that they do not meet all five criteria of indigenous peoples as set in the policies of the prospective international financiers. Green Alternative and Bankwatch find this judgement arbitrary and contrary to the United Nation's definition of indigenous peoples.	Green Alternative (2012)

Poor or no E(S)IA	the ESIA does not specify how the permanent loss of customary land for people who have no formal land titles or recognisable claims will be handled.	Green Alternative (2012)
Poor or no E(S)IA	while the draft Environmental and Social Impact Assessment importantly considers international environmental treaties and international water laws, it does not consider relevant international human rights instruments regarding resettlement.	Human Rights Watch (2014)
Poor or no E(S)IA	All necessary environmental impact analyses have to be concluded and evaluated before the start of project implementation. Moreover, it has to be ensured that the recommendations given can actually be translated into practice. In the case of the project "Lower Kihansi Hydropower Station" the environmental impact studies were obviously conducted too late	KFW (2006)
Poor or no E(S)IA	Because of the lack of studies on the project's impacts, some of the damage caused by the dam is only now becoming apparent, including the possibility that water from the reservoir may be seeping into Argentina's Iberá wetlands, destroying the region's rich biodiversity.	International Rivers (n.d./a)

References

Preliminary note: those documents that have no hyperlink or DOI were accessed through our institution's library

Africa Resources Working Group (2009). A Commentary on the Environmental, Socioeconomic and Human Rights Impacts of the Proposed Gibe III Dam in the Lower Omo River Basin of Ethiopia. Retrieved 30 March 2021 from http://www.forestpeoples.org/sites/fpp/files/publication/2010/08/ethiopiahydroelecimpactsarwgjan09eng.pdf

ABC News (2013). Egyptian politicians caught discussing plan to sabotage Ethiopian dam. Retrieved 20 October 2021 from https://www.abc.net.au/news/2013-06-05/egyptian-politicians-caught-in-on-air-ethiopia-gaffe/4733544

Al-Ansari , N., Adamo, N., Issa, E., Sissakian, V.K., Knutsson, S. (2015). Mystery of Mosul Dam the Most Dangerous Dam in the World: Dam Failure and its Consequences. Journal of Earth Sciences and Geotechnical Engineering 5(3) 95-111. Retrieved 15 August 2021 from https://www.researchgate.net/publication/275949388_Mystery_of_Mosul_Dam_the_Most_Dangerous_Dam_in_the_World_Dam_Failure_and_its_Consequences

Allibhai, N. (2015). "Come and count our bones" – Community voices from the Lake Turkana on the impacts of the Gibe III dam. International RiversAnsar, A., Flyvbjerg, B., Budzier, A. and Lunn, D. (2014). Should we build more large dams? The actual costs of hydropower megaproject development. Energy Policy 69: 43–56. https://doi.org/10.1016/j.enpol.2013.10.069

Annunziato, A., Andreadakis, I., Probst, P. (2016). Impact of flood by a possible failure of the Mosul dam. JRC Publications Repository. Retrieved 20 October 2021 from https://publications.jrc.ec.europa.eu/repository/handle/JRC101555

Balazote, O. A. and Radovich, J. C. (2003). Grandes represas hidroeléctricas: efectos sociales sobre poblaciones Mapuches en la Región del Comahue, Argentina [in English: *Big hydroelectric dams: social effects on Mapuche peope in Comahue Region, Argentina*]. In Silvio Coelho dos Santos y Aneliese Nacke (Orgs.) Hidrelétricas e povos indígenas [in English: *Hydroelectric facilities and indigenous people*]. Ed. Letras Contemporáneas, Florianópolis, Brazil. Retrieved 20 August 2021 from https://www.researchgate.net/publication/263542596_Grandes_represas_hidroelectricas_efectos_sociales_sobre_poblaciones_Mapuches_en_la_Region_del_Comahue_Argentina

BBC News (2009). Species' extinction threat grows. Retrieved 20 August 2021 from http://news.bbc.co.uk/2/hi/science/nature/8338880.stm#:~:text=The%20Kihansi%20Spray%20Toad%20(Nectophyrnoides,of%20an%20estimated%2017%2C000%20individuals.

BBC News (2020). River Nile dam: Sudan blasts 'unilateral' move as Ethiopia dam fills. Retrieved 20 October 2021 from https://www.bbc.com/news/world-africa-53429014

Bender, J. (2014). ISIS Has Seized Iraq's Largest Dam, And What Happens Next Is Critical. Retrieved 24 December 2020 from https://www.businessinsider.com.au/isis-has-seized-the-mosul-dam-2014-8/

Bennet, O. and McDowell, C. (2012). Displaced – The Human Cost of Development and Resettlement. Palgrave Macmillan, New York, US.

Bibbo, B. (2016). Mosul Dam collapse ‘will be worse than a nuclear bomb’. Retrieved 20 October 2021 from https://www.aljazeera.com/features/2016/12/11/mosul-dam-collapse-will-be-worse-than-a-nuclear-bomb

Borger, J. (2016). Mosul dam engineers warn it could fail at any time, killing 1m people. The Guardian. Retrieved 10 November 2020 from https://www.theguardian.com/world/2016/mar/02/mosul-dam-engineers-warn-it-could-fail-at-any-time-killing-1m-people

Business and Human Rights Resource Center (2017). Colombia: Comunidades de Santander son reprimidas en protestas por impactos negativos de Hidrosogamoso [in English: *Colombia: Santander communities are repressed in protest for negative impacts from Hidrosogamoso*].

Retrieved 24 July 2020 from https://www.business-humanrights.org/es/colombia-comunidades-de-santander-son-reprimidas-en-protestas-por-impactos-negativos-de-hidrosog%E2%80%A6

Carr, C. J. (2017). River Basin Development and Human Rights in Eastern Africa – A Policy Crossroads. Springer

CENSAT-AguaViva (n.d.). Las deudas de Hidrosogamoso [in English: *Hidrosogamoso's debts*]. Retrieved 24 July 2020 from https://censat.org/es/noticias/las-deudas-de-hidrosogamoso

Centro Documentazione Conflitti Ambientali (n.d.). Diga sul fiume Niger e diga sul fiume Sokoto [in English: *Dam on Niger river and dam on Sokoto river*]. Retrieved 26 June 2020 from http://cdca.it/en/archives/10120

Chenga, N. (2014). Cheap design linked to problems at Tokwe-Mukosi. Nihanda Radio. Retrieved 11 July 2020 from https://nehandaradio.com/2014/02/15/cheap-design-linked-problems-tokwe-mukosi/

Chipashvili, D. (2017). Price tag of Georgia's Nenskra dam goes through the roof. Bankwatch.org. Retrieved 20 October 2021 from https://bankwatch.org/blog/price-tag-of-georgia-s-nenskra-dam-goes-through-the-roof

Cloete, L. (2016). Strike looms at Neckartal Dam. The Namibian. Retrieved 20 August 2021 from https://www.namibian.com.na/152425/archive-read/Strike-looms-at-Neckartal-Dam

Cloete, L. (2016a). Workers accuse police of siding with Salini. The Namibian. Retrieved 20 August 2021 from https://www.namibian.com.na/152600/archive-read/Workers-accuse-police-of-siding-with-Salini

Colajacomo, J. (1999). The Chixoy Dam: The Maya Achi' Genocide. The Story of Forced Resettlement. Working paper of the World Commission on Dams. Cape Town, South Africa. Retrieved 20 August 2021 from http://rio-negro.info/che/doc/ChixoyDam_StoryOfForcedResettlement.pdf

Cree Nation of Waskaganish (n.d.) The James Bay Project. Retrieved 20 July 2021 from https://waskaganish.ca/the-james-bay-project/

Curran, P. (2012). Trip back in corruption time machine. Montreal Gazette. Retrieved 10 November 2020 from http://www.montrealgazette.com/news/trip+back+corruption+time+machine/7328187/story.html

D'Angelo, L. (2014). Changing Environments, Occult Protests, and Social Memories in Sierra Leone. Social Evolution & History, 13(2), 22-56. Retrieved 20 August 2021 from https://www.sociostudies.org/journal/files/seh/2014_2/022-056.pdf

Darbourn, K. (2015). Impact of the failure of the Kariba dam. The Institute of Risk Management South Africa. Retrieved 20 July 2021 from https://cdn.ymaws.com/www.irmsa.org.za/resource/resmgr/2016_case_studies/kariba_report.pdf

Dearden, N. (2012). Guatemala's Chixoy dam: where development and terror intersect. The Guardian. Retrieved 24 December 2020 from https://www.theguardian.com/global-development/poverty-matters/2012/dec/10/guatemala-chixoy-dam-development-terror

Democracy and Freedom Watch (n.d.). Svan council convenes to stop hydro power plants. Retrieved 20 July 2021 from https://dfwatch.net/svan-ialkhor-council-convenes-to-stop-hydro-power-plants-43212

Democracy and Freedom Watch (2020). Nenskra. Retreived 24 December 2020 from https://dfwatch.net/?s=nenskra&lang=en

Dussán Calderón, M. (2016). El Quimbo: un desastre anunciado. Retrieved 7 July 2020 from http://millerdussan.blogia.com/2016/011601-el-quimbo-un-desastre-anunciado.php

Ecosistemas (2014). ANGOSTURA: EL INICIO DE LA PRIMERA GRAN HIDROELÉCTRICA EN LA ÚLTIMA DÉCADA [in English: *ANGOSTURA: THE BEGINNING OF THE FIRST BIG HYDROELECTRIC FACILITY IN THE LAST DECADE*]. Retrieved 21 August 2021 from https://www.ecosistemas.cl/2014/07/angostura-el-inicio-de-la-primera-gran-hidroelectrica-en-la-ultima-decada/

ElDesconcierto (2014). La hidroeléctrica Angostura y el desastre del Biobío [in English: *the Angostura hydroelectric facility and the BioBio disaster*]. Retrieved 24 December 2020 from https://www.eldesconcierto.cl/bienes-comunes/2014/07/08/la-hidroelectrica-angostura-y-el-desastre-del-biobio.html

Eliécier Quintero, J. (2007). Mortandad de peces en Betania, la más grande del país por verano [in English: *Fish mortality in Betania, the biggest in the country in summer*]. El Tiempo. Retrieved 20 August 2021 from https://www.eltiempo.com/archivo/documento/MAM-2400673

El Pitazo (2019). Un fiscal en el exilio revela detalles de la corrupción de Odebrecht en Tocoma [in English: *Exiled prosecutor reveals details of Odebrecht corruption in Tocoma*]. Retrieved 31 March 2021 from https://elpitazo.net/politica/proyecto-tocoma-la-central-hidroelectrica-que-quedo-en-promesas-tras-12-anos/

Filkins, D. (2016). A Bigger Problem Than ISIS? The New Yorker. Retrieved 31 March 2021 from https://www.newyorker.com/magazine/2017/01/02/a-bigger-problem-than-isis

Forti, M. (2016). L'esercito italiano a Mosul fa da scorta a un'azienda privata? [in English*: Is the Italian army in Mosul escorting a private company*?]. Internazionale. Retrieved 20 August 2021 from https://www.internazionale.it/opinione/marina-forti/2016/10/18/militari-italiani-mosul-diga

Franchi, G. and Manes, L. (2016). What is there to hide in the Omo Valley? The shadowy Italian system in Ethiopia. Re:Common, Rome, Italy. Retrieved 20 August 2021 from https://www.recommon.org/en/hide-omo-valley/

Galindo Vanegas, A. S. (2018). Cambios en los conflictos ambientales generados por la construcción de las Centrales Hidroeléctricas de Betania y El Quimbo [in English: *Changes in the environmental conflicts generated by the construction of the Betania and El Quimbo hydroelectric power plants*]. Master thesis at Universidad Nacional de Colombia. Bogotá, Colombia. Retrieved 14 August 2021 from https://repositorio.unal.edu.co/handle/unal/63714

Gerebizza, E. (2009). El proyecto Daule Peripa Las responsabilidades italianas en la deuda ilegítima de Ecuador [in English: *The Daule Peripa project Italian responsibilities in Ecuador's illegitimate debt*]. Campagna per la Riforma della Banca Mondiale, Rome, Italy. Retrieved 20 July 2021 from http://www.deudaecologica.org/documentos/tipos%20de%20deuda/el_projecto_daule_peripa_crbm.pdf

Global Nonviolent Actions Database (2011). Icelanders protest Karahnjukar Hydropower Project, 2000-2006. Retrieved 20 August 2021 from https://nvdatabase.swarthmore.edu/content/icelanders-protest-karahnjukar-hydropower-project-2000-2006

Green Alternative (2012). Nenskra Dam. Retrieved 24 July 2020 from https://greenalt.org/nenskra/

Guatemala Human Rights Commission (2011). Rio Negro Massacres. Retrieved 10 November 2020 from https://www.ghrc-usa.org/our-work/important-cases/rio-negro/

Hilton, T. E. (1966). Akosombo dam and the Volta river project. Geography 51(3), 251-254. Retrieved 22 August 2021 from https://www.jstor.org/stable/40567076

Himdara (2015). Kinnaur residents rise-up! Demand immediate action on pathetic condition of NH-5. Retrieved 20 July 2020 from www.himdhara.org/2015/06/16/press-note-15th-june-2015-kinnaur-residents-rise-up-demand-immediate-action-on-pathetic-condition-of-nh-5/

Hodbod, J., Stevenson, E.G.J., Akall, G. et al. (2019). Social-ecological change in the Omo-Turkana basin: A synthesis of current developments. Ambio 48, 1099–1115. https://doi.org/10.1007/s13280-018-1139-3

Human Rights Watch (2014). “We Suffered When We Came Here” Rights Violations Linked to Resettlements for Tajikistan’s Rogun Dam. Retrieved 31 March 2021 from https://www.hrw.org/report/2014/06/25/we-suffered-when-we-came-here/rights-violations-linked-resettlements-tajikistans

Human Rights Watch (2015). Homeless, Landless, and Destitute The Plight of Zimbabwe’s Tokwe-Mukorsi Flood Victims. Retrieved 31 March 2021 from https://www.hrw.org/sites/default/files/reports/zimbabwe0215_ForUpload.pdf

Hussein, H. (2014). Egypt and Ethiopia spare over the Nile. Al Jazeera America. Retrieved 24 December 2020 from http://america.aljazeera.com/opinions/2014/2/egypt-disputes-ethiopiarenaissancedam.html

Iceland Review Online (2013). Impacts of Iceland's Karahnjukar Dam More Serious Than Expected. Retrieved 20 August 2021 from https://damsandalternatives.blogspot.com/2013/03/impacts-of-icelands-karahnjukar-dam.html

INFOAut (2016). Mosul: cosa nasconde la diga di Renzi [in English: *Mosul: what Renzi's dam hides*]. Retrieved 20 August 2021 from https://www.infoaut.org/prima-pagina/mosul-cosa-nasconde-la-diga-di-renzi

International Rivers (n.d.) Bujagali Dam, Uganda. Retrieved 11 July 2020 from https://www.internationalrivers.org/campaigns/bujagali-dam-uganda

International Rivers (n.d./a). Yacyretá Dam. Retrieved 18 June 2020 from https://www.internationalrivers.org/campaigns/yacyretá-dam

International Rivers (1999). Lesotho Highlands Water Project, World Bank's Responsibilities and Impregilo Involvement in Corruption Court Case. Retrieved 11 July 2020 from https://www.internationalrivers.org/resources/lesotho-highlands-water-project-world-bank's-responsibilities-and-impregilo-involvement-in

International Rivers (2001). Lower Kihansi Hydropower Project: An Evaluation of the Project Against World Commission on Dams Guidelines. Retrieved 26 June 2020 from https://www.internationalrivers.org/resources/lower-kihansi-hydropower-project-an-evaluation-of-the-project-against-world-commission-on

International Rivers (2005). A Brief History of Africa's Largest Water Project. Retrieved 11 July 2020 from https://www.internationalrivers.org/resources/a-brief-history-of-africa's-largest-water-project-3664

International Rivers (2009). Ethiopia's Gibe 3 Dam: Sowing Hunger and Conflict. International Rivers. Retrieved 27 July 2020 from https://archive.internationalrivers.org/resources/ethiopia-s-gibe-iii-dam-sowing-hunger-and-conflict-2643

International Rivers (2009a). Kariba Dam Safety Concerns. Retrieved 11 July 2020 from https://www.internationalrivers.org/resources/kariba-dam-safety-concerns-3560

International Rivers (2013). Ethiopia's biggest dam oversized, experts say. Retrieved 13 July 2020 from https://www.internationalrivers.org/resources/ethiopia's-biggest-dam-oversized-experts-say-8082

International Rivers (2014). The Grand Ethiopian Renaissance Dam Fact Sheet. Retrieved 13 July 2020 from https://www.internationalrivers.org/resources/the-grand-ethiopian-renaissance-dam-fact-sheet-8213

International Rivers (2017). 5 Myths Surround the Grand Ethiopian Renaissance Dam (GERD). Retrieved 13 July 2020 from https://www.internationalrivers.org/blogs/not-yet-assigned/5-myths-surround-the-grand-ethiopian-renaissance-dam-gerd

IUCN (2018). Lake Turkana listed as 'in danger' due to impacts from dam, as advised by IUCN. Retrieved 20 August 2021 from https://www.iucn.org/news/iucn-42whc/201806/lake-turkana-listed-danger-due-impacts-dam-advised-iucn

Johnston, B. R. (2010). Chixoy dam legacies: The struggle to secure reparation and the right to remedy in Guatemala. Water Alternatives 3(2): 341-361. Retrieved 20 July 2021 from https://biblioteca.cejamericas.org/bitstream/handle/2015/1498/Chixoy-Dam-Legacies-The-Struggle-to-Secure-Reparation-and-the-Right-to-Remedy-in-Guatemala.pdf?sequence=1&isAllowed=y

KFW (2006). Tanzania: Lower Kihansi Hydropower Station. Retrieved 21 August 2021 from https://www.kfw-entwicklungsbank.de/migration/Entwicklungsbank-Startseite/Development-Finance/Evaluation/Results-and-Publications/PDF-Dokumente-R-Z/KFW (2006).pdf

Khadka, N. S. (2003). Big hydro, big hanky-panky? The Nepali Times. Retrieved 10 November 2020 from archive.nepalitimes.com/issue/166/Development/4864#.XvXzC5MzZo5

Kourouma, D. L., Lamah, O. O. (n.d.). Analyse de l'impact sr la santé des populations locales du barrage hydroélectrique de Garafiri en Guinée [in English : *Analysis of the impact on the health of local populations of the Garafiri hydroelectric dam in Guinea*]. Retrieved 20 August 2021 from https://www.sifee.org/static/uploaded/Files/ressources/actes-des-colloques/bamako/pleniere-2/A_Kourouma_etal_comm.pdf

Lang, C., Hildyard, N., Geary, K. and Grainger, M. (2000). Dams Incorporated – The Record of Twelve European Dam Building Companies. The CornerHouse. The Swedish Society for Nature Conservation. Retrieved 31 March 2021 from http://www.thecornerhouse.org.uk/resource/dams-incorporated

Lela Mobile Online (2016). Salini workers strike, lock gate. 27-06-2016. Retrieved 22 June 2020 from https://www.nampa.org/index.php?model=feature&function=display&id=126319

Lenka Thamae, M. and Pottinger, L. (2006). On the Wrong Side of Development - Lessons Learned from the Lesotho Highlands Water Project. Transformation Resource Center, Maseru, Lesotho. Retrieved 20 August 2021 from https://archive.internationalrivers.org/sites/default/files/attached-files/wrongside2006.pdf

Mambo, E. (2014). Mujuru sucked in messy land row. Zimbabwe Independent. Retrieved 14 July 2020 from https://www.theindependent.co.zw/2014/04/25/mujuru-sucked-messy-land-row/

Manes, L. (2012). Il caso Palo Viejo in Guatemala – La diga della discordia nelle terre dei Maya. Re:Common, Rome, Italy. Retrieved 20 July 2021 from https://www.recommon.org/il-caso-palo-viejo-in-guatemala/

Mazzei, L. and Scuppa, G. (2006). The Role of Communication in Large Infrastructure - The Bumbuna Hydroelectric Project in Post-Conflict Sierra Leone. World Bank Working Paper, Washington D.C., US. Retrieved 22 August 2021 from https://openknowledge.worldbank.org/bitstream/handle/10986/7110/368190CE0Large1C0disclosed0JULY0191.pdf?sequence=1&isAllowed=y

McCully, P. (2001). Silenced rivers: the ecology and politics of large dams. Zed Books, London, UK.

Medellin Mazzeo, A. (2001). Impregilo – I crimini del capitalismo italiano nel mondo [in English: *Impregilo – the crimes of Italian capitalism in the world*]. Retrieved 21 August 2021 from http://www.autprol.org/public/news/doc000401605072009.htm

Moreno Socha, J. M. (2019). Energías del despojo: Desplazamiento forzado por megaproyectos, memoria y resistencia en torno a Hidrosogamoso [in English: *Energies of dispossession: Forced displacement by megaprojects, memory and resistance around Hidrosogamoso*]. Proceedings at the X Latino-American Congress in Political Science. Monterrey, Mexico. Retrieved 20 July 2021 from https://repositorio.unal.edu.co/handle/unal/76873

Namibia News Digest (2015). Salini and workers reach agreement. Retrieved 22 June 2020 from https://www.namibianewsdigest.com/%EF%BB%BFsalini-and-workers-reach-agreement/

Namibian Sun (2015). Dam builders to get shade, water and toilets. Retreived 25 July 2020 from https://www.namibiansun.com/main/print_post/dam-builders-to-get-shade-water-and-toilets/

National Association of Professional Environmentalists Uganda (2014). Unsettling Business - Social consequences of the Bujagali hydropower project. FIVAS, Oslo, Norway. Retrieved 10 August 2021 from http://fivas.org/wp-content/uploads/2016/04/fivas_unsettlingbusiness_skjerm.pdf

New Era Live (2014). Salini in bid to save face. Retrieved 11 June 2020 from https://neweralive.na/posts/salini-bid-save-face

New Era Live (2016). Neckartal Dam workers claim victimisation by supervisors. Retrieved 15 July 2021 from https://neweralive.na/posts/neckartal-dam-workers-claim-victimisation-supervisors

New Era Live (2017). Salini to reimbourse suspended workers. Retrieved 20 June 2020 from https://neweralive.na/posts/salini-to-reimburse-suspended-workers

OECD Watch (2017). Survival International vs. Salini Impregilo. Retrieved 19 December 2020 from https://complaints.oecdwatch.org/cases/Case_459

OLCA (2014). Central Hidroeléctrica Angostura: La influencia del poder económico por sobre el Estado [in English: *Angostura Hydroelectric Power Plant: The influence of economic power over the State*]. Retrieved 20 August 2021 from https://olca.cl/articulo/nota.php?id=104601

Osses, S. (2014). Vecinos de Quilaco, Alto Bío Bío y Santa Bárbara protestan contra centrales hidroeléctricas [in English: *Neighbours of Quilaco, Alto Bío Bío and Santa Bárbara protest against hydroelectric power plants*]. Retrieved 20 August 2021 from https://www.biobiochile.cl/noticias/2014/01/10/vecinos-de-quilaco-alto-bio-bio-y-santa-barbara-protestan-contra-centrales-hidroelectricas.shtml

Owusu, K., Bilson Obur, P., Asiwah Nkansah, M. (2016). Downstream effects of dams on livelihoods of river-dependent communities: the case of Ghana’s Kpong Dam, Geografisk Tidsskrift-Danish Journal of Geography, DOI:10.1080/00167223.2016.1258318

Pittalunga, F., Oumarou, N., Juliette, A., Alain, K., Youssouf, N., Salvati, N. and Seghieri, C. (2002). Profil de pauvreté des communautés riveraines du lac de Kossou en Côte d'Ivoire [in English : *Poverty profile of the communities bordering Lake Kossou in Côte d'Ivoire*]. FAO program PMEDP/SFLP, Cotonou, Benin.

Poliszuk, J., Marcano, P. and Castro, M. (2018). La obra maestra de la corrupción de Odebrecht fue digna de un galería de arte [in English: *Odebrecht's corruption masterpiece was worthy of an art gallery*]. In Armando.info. Retrieved 31 March 2021 from https://armando.info/Reportajes/Details/2478

Prowizur, E. (1976). Les effets humains du barrage Kossou sur le Bandama blanc (Rép. Côte d'Ivoire) [in English: *Kossou dam on the Bandama river: its effects on the population (central Ivory Coast)*]. Civilisations, 26(3/4), 232-258. Retrieved 21 July 2020 from https://www.jstor.org/stable/41803047

Quinn, C. H., Ndanglasi, H. J., Gerstle, J. and Lovett, J. C. (2005). Effect of the Lower Kihansi Hydropower Project and post-project mitigation measures on wetland vegetation in Kihansi Gorge, Tanzania. Biodiversity and Conservation 14, 297-308. https://doi.org/10.1007/s10531-004-5048-3

Raphaël, O. K., Sylverstre, K. K. and Yao K. S. (2019). Deplacement Involontaire De Populations Et Conflits Fonciers A Ayaou-Sokpa (Sous-Prefecture D'ayaouSran, Centre De La Cote d'Ivoire) [in English : *Involuntary Displacement of Populations and Land Conflicts in Ayaou-Sokpa (Sub-Prefecture of Ayaou-Sran, Central Cote d'Ivoire)*]. European Scientific Journal, 15(2), 85-102. https://doi.org/10.19044/esj.2019.v15n2p85

Roa Avendaño, T. and Duarte Abadía, B. (2012). Aguas Represadas - El caso del proyecto Hidrosogamoso en Colombia [in English: *Dammed Waters - The Case of the Hidrosogamoso Project in Colombia*]. Ediciones Antropos, Bogotá, Colombia.

Roa Avendaño, T. and Duarte Abadía, B. (2013). DESARROLLO HIDROELÉCTRICO, DESPOJO Y TRANSFORMACIÓN TERRITORIAL: El caso de Hidrosogamoso, Santander, Colombia [in English: *HYDROELECTRIC DEVELOPMENT, DISPLACEMENT AND TERRITORIAL TRANSFORMATION: The Case of Hidrosogamoso, Santander, Colombia*]. In: Aguas Robadas. Despojo Hídrico y Movilización Social [in English: *Stolen Waters. Water Dispossession and Social Mobilisation*]. Editors: Aline Arroyo y Rutgerd Boelens. Quito, Ecuador. Retrieved 21 July 2021 from https://totumasymaracas.files.wordpress.com/2013/10/doc_tati-bibi_art-hidrosogamoso_aguas-robadas_2013_rfinal.pdf

Roa Avendaño, T. (2016). Hidrosogamoso: Conflicto y resistencia. Revista Semillas. Retrieved 20 August 2021 from https://semillas.org.co/es/hidrosogamoso-conflicto-y-resistencia

Roussi, A. (2020). Rowover Africa's largest dam in danger of escalating, warn scientists. Nature. Retrieved 20 August 2021 from https://www.nature.com/articles/d41586-020-02124-8

Saving Iceland (2005). A Statement from Kárahnjúkar Protest Camp. Retrieved 20 August 2021 from https://www.savingiceland.org/2005/07/a-statement-from-karahnjukar-protest-camp/

Saving Iceland (2007). Rising Ecocide: Nests Swallowed by Water at Kárahnjúkar. Retrieved 20 August 2021 from https://www.savingiceland.org/2007/05/rising-ecocide-nests-swallowed-by-water-at-karahnjukar/

Salini Impregilo (2013). Sustainability Report 2013. Retrieved 30 March 2021 from https://media.webuildgroup.com/sites/default/files/2019-10/salini-impregilo-sustainability-report-2013.pdf

Salini Impregilo (2014). Sustainability Report 2014. Retrieved 31 March 2021 from https://media.webuildgroup.com/sites/default/files/2019-10/2014%20Salini%20Impregilo%20Sustainability%20Report.pdf

Salini Impregilo (2015a). Sustainability Report 2015. Retrieved 31 March 2021 from https://media.webuildgroup.com/sites/default/files/2019-10/report_sostenibilita-_salini-web_compressed.pdf

Salini Impregilo (2016b). Sustainability Report 2016. Retrieved 30 March 2021 from https://media.webuildgroup.com/sites/default/files/2019-10/2016%20Salini%20Impregilo%20Sustainability%20Reports.pdf

Salini Impregilo (2017a). Annual Report 2017. Retrieved 31 March 2021 from https://media.webuildgroup.com/sites/default/files/2019-10/2017%20Salini%20Impregilo%20Sustainability%20Reports%20EN.pdf

Scudder, T. (2005). The Kariba Case Study. Working Paper 1227, California Institute of Technology, California, US. Retrieved 22 July 2021 from https://authors.library.caltech.edu/79966/

Semana (2019). La Esmeralda - Lo que el embalse dejó [in English: *La Esmeralda - What the reservoir left behind*]. Retrieved 20 December 2020 from https://sostenibilidad.semana.com/medio-ambiente/articulo/la-esmeralda--lo-que-el-embalse-dejo/43397

Showstack, R. (2012). Mesopotamian fertile crescent nearly gone, new study indicates. Eos 82(24) 262-267. https://doi.org/10.1029/EO082i024p00262

Skoba, L. (2013). Transboundary water management - The Rogun Dam in Tajikistan. Briefing from the Library of the European Parliament, 130621REV1. Retrieved 24 December 2020 from https://www.europarl.europa.eu/RegData/bibliotheque/briefing/2013/130621/LDM_BRI(2013)130621_REV1_EN.pdf

Stahl, D. J. (2019). A technopolitical frontier-The Keban Dam project and southeastern Anatolia. Routlege. ISBN 9780429429699

Survival International (2019). Popoli della Valle dell'Omo. Retrieved 20 August 2021 from https://www.survival.it/popoli/valleomo

Terol, G. and Reid, J. (2004). Beneficios y Costos de Elevar la Cota del Proyecto Hidroeléctrico de Yacyretá. Retrieved 20 August 2021 from https://www.conservation-strategy.org/sites/default/files/field-file/0_15_Terol and Reid (2004).pdf

Thanju, R. P. (2008). Kali Gandaki "A" Hydroelectric Project in Environmental Perspectives. Hydro Nepal: Journal of Water, Energy and Environment, 1(0). https://doi.org/10.3126/hn.v1i0.880

The Oakland Institute (2019). “How They Tricked Us” Living With the Gibe III Dam And Sugarcane Plantations In Southwest Ethiopia. Oakland, CA, US. Retrieved 20 August 2021 from https://www.oaklandinstitute.org/sites/oaklandinstitute.org/files/ethiopia-tricked-gibe-dam-sugarcane-plantations.pdf

Thorarins, F. (2013). The rise and fall of temporary staffing agencies in Iceland. In Horgen Friberg, J. and Eldring L. (eds.), Thorarins (2013) Patterns of migration, working conditions and recruitment practices, 301-327, Nordic Council of Ministers, Copenhagen, Denmark.

Tjihenuna, T. (2014). Neckartal workers claim abuse by Impregilo. Namibian.com. Retrieved 31 March 2021 from https://www.namibian.com.na/123603/archive-read/Neckartal-workers-claim-abuse-by-Impregilo

Transparencia Venezuela (2018). 11 años después del inicio de su construcción Tocoma sigue sin dar luz [in English: *11 years after the start of its construction, Tocoma still does not provide electricity*]. Retrieved 10 November 2020 from https://transparencia.org.ve/11-anos-despues-del-inicio-de-su-construccion-tocoma-sigue-sin-dar-luz/

Tribune India (1999). Nathpa-Jhakri project workers trike work. Retrieved 21 August 2021 from https://www.tribuneindia.com/1999/99apr23/himachal.htm#1

Velpuri, N. M. And Senay, G.B. (2012). Assessing the potential hydrological impact of the Gibe III Dam on Lake Turkana water level using multi-source satellite data. Hydrol. Earch Syst. Sci., 16, 3561-3578. doi:10.5194/hess-16-3561-2012

Wall, D. (2017). The battle for James Bay. Daily Commercial News. ConstructConnect Canada. Retrieved 10 November 2020 from https://canada.constructconnect.com/dcn/news/infrastructure/2017/11/the-battle-for-james-bay-1028910w

Zane, D. (2020). Nile Dam row: Egypt and Ethiopia generate heat but no power. Retrieved 20 August 2021 from https://www.bbc.com/news/world-africa-53327668

Zelalem, Z. (2020). An Egyptian cyber attack on Ethiopia by hackers is the latest strike over the Grand Dam. Quartz Africa. Retrieved 20 October 2020 from https://qz.com/africa/1874343/egypt-cyber-attack-on-ethiopia-is-strike-over-the-grand-dam/
